# Identification and expression analysis of an olfactory receptor gene family in green plant bug *Apolygus lucorum* (Meyer-Dür)

**DOI:** 10.1038/srep37870

**Published:** 2016-11-28

**Authors:** Xing-Kui An, Liang Sun, Hang-Wei Liu, Dan-Feng Liu, Yu-Xiao Ding, Le-Mei Li, Yong-Jun Zhang, Yu-Yuan Guo

**Affiliations:** 1 State Key Laboratory for Biology of Plant Diseases and Insect Pests, Institute of Plant Protection, Chinese Academy of Agricultural Sciences, Beijing100193, 100193, China; 2 Key Laboratory of Tea Biology and Resources Utilization, Ministry of Agriculture, Tea Research Institute, Chinese Academy of Agricultural Sciences, Hangzhou, 310008, China; 3 College of Agronomy, Jilin Agricultural University, Changchun, Jilin 130118, China

## Abstract

Olfactory receptors are believed to play a central role in insects host-seeking, mating, and ovipositing. On the basis of male and female antennal transcriptome of adult *Apolygus lucorum*, a total of 110 candidate *A. lucorum* odorant receptors (*AlucOR*) were identified in this study including five previously annotated *AlucORs*. All the sequences were validated by cloning and sequencing. Tissue expression profiles analysis by RT-PCR indicated most *AlucORs* were antennal highly expressed genes. The qPCR measurements further revealed 40 *AlucORs* were significantly higher in the antennae. One *AlucOR* was primarily expressed in the female antennae, while nine *AlucORs* exhibited male-biased expression patterns. Additionally, both the RPKM value and RT-qPCR analysis showed *AlucOR83* and *AlucOR21* were much higher abundant in male antennae than in female antennae, suggesting their different roles in chemoreception of gender. Phylogenetic analysis of ORs from several Hemipteran species demonstrated that most *AlucORs* had orthologous genes, and five AlucOR-specific clades were defined. In addition, a sub-clade of potential male-based sex pheromone receptors were also identified in the phylogenetic tree of *AlucORs*. Our results will facilitate the functional studies of *AlucORs*, and thereby provide a foundation for novel pest management approaches based on these genes.

The detection and discrimination of semiochemicals in the environment by specialized sensilla plays an important role in insect survival and reproduction^1^,^2^. For insects, chemosensory sensilla distribute over the surface of chemosensory tissues including antennae, palps, mouth parts, tarsi, and many other organs^3^, which mediate many key behaviors, such as host-seeking, mate choice, oviposition site selection, and predator avoidance^2^. The antenna is a specialized organ for insect sensing, which is the most significant organs of olfaction, housing thousands of olfactory sensory neurons (OSNs) that extend their dendrites up into the sensilla and project their axons towards the brain^4^,^5^. Volatile chemicals can be transformed into electrical signals by these OSNs and then these signals were preliminarily integrated and sent to higher brain centers by antennal lobe (AL) to finally generate a behavioral response^6^. Diverse olfactory proteins are evolved in this olfactory sensation process, including odorant-binding proteins (OBP), chemosensory proteins (CSPs), sensory neuron membrane proteins (SNMPs), odorant-degrading enzymes (ODEs), ionotropic receptors (IRs), and odorant receptors (ORs)^2^,^3^,^7^,^8^. OBPs are thought to be the first proteins that selectively bind to liposoluble odor molecules, and acted as a carrier to transport odorants through water-soluble lymph within sensilla to the ORs in the membrane of ORN dendrites^9^. After activating the ORs, distinct ODEs will degrade odorants and maintain the sensitivity of ORNs^2^,^7^,^10^. Thus far, information on this peripheral olfactory process is very limited, especially in the odorants inactivation step.

Insects mainly rely on ORs to perform the long distance detection of volatile molecules^11^. ORs, which span the dendritic membrane with seven alpha helices, and present an inverted topology (intracellular N-terminus) compared with mammalian ORs, are responsible for the conversion of chemical message to an electrical signal^12^. A typical OR unit functions as a dimer complex with the odorant receptor co-receptor (Orco), which is highly conserved among insect species^13^. Orco is believed to interact with each of the divergent ORs forming ligand-gated ion channels and to enhance odorant responsiveness^12^,^14^. Since the first insect ORs discovered in the fruit fly *Drosophila melanogaster*^5^,^15^, multiple OR repertoires have been identified in a variety of insect species through whole-genome sequencing, including Diptera, Hymenoptera, Lepidoptera, Coleoptera, Hemiptera, and Blattodea. The number of OR genes varies significantly from 62 in *Drosophila melanogaster* to 259 in *Tribolium castaneum* and up to 350 in *Camponotus floridanus*^15^,^16^,^17^, reflecting a various evolution of insect OR genes. Silencing of the olfactory co-receptor gene in *Apolygus lucorum, Lymantria dispar* and *Dendroctonus armandi* leads to electroantennographic (EAG) response declining to major semiochemicals^18^,^19^,^20^. In recent years, many Lepidoptera insect pheromone receptors were explored by using the *Xenopus* oocyte expression system^21^,^22^,^23^. However, to date, the exact functions of insect OR genes are largely unknown.

The green plant bug *A. lucorum* is one of the most destructive agricultural insects in China, feeding on over 150 recorded host plants^24^, including cotton, fruits, and vegetables^25^,^26^. With successful promotion and cultivation of transgenic insect resistant cotton since 1997, the population of this non-target pest has been increasing gradually in the past two decades, causing large economic losses^27^. Not surprisingly, the widespread planting of *Bacillus thuringiensis* (Bt) cotton have effectively controlled Lepidopteran pests and reduced the use of chemical pesticides, and as a side effect causing secondary non-target insects becoming major pests in the cotton field^27^. It was reported that *A. lucorum* was a typical representative that emerged as the key pest after the wide adoption of Bt cotton. To target and exploit simple and effective coping strategies, lots of studies on *A. lucorum* have been performed, including its physiology, chemoecology, and insecticide resistance in order to develop novel control methods^28^,^29^. However, the molecular components and mechanisms comprising *A. lucorum* olfactory system that could be potential novel targets for controlling this mirid bug have not been fully elucidated. In field experiments, six electrophysiological active compounds including m-xylene, butyl acrylate, butyl propionate, butyl butyrate, (*Z*)-3-hexen-1-ol, and (*Z*)-3-hexenyl acetate from flowering *Artemisia* plants were considered to be the effective substance to attract *A. lucorum* adults^30^. Moreover, an antenna highly expressed olfactory receptor gene, *AlucOR28*, was identified to sensitively tune to (*Z*)-3-hexenyl acetate and several flowering compounds^31^. Further study on the molecular mechanisms underlying the detections of these compounds is needed.

The identification of genes encoding OR families is a key step toward understanding the characteristics of *A. lucorum* olfactory systems. However, till now, our understandings for the OR genes of *A. lucorum* at the molecular level is very limited. To elucidate the molecular mechanism of *A. lucorum* olfactory system and to design novel coping strategies against this green plant bug, we performed a transcriptome analysis of both female and male antennae, and a total of 110 OR genes from *A. lucorum* were identified successfully. The expression patterns of these candidate ORs in different tissues were also examined by using reverse transcription PCR (RT-PCR) and quantitative real-time PCR (qPCR) in this study.

## Results

### Analysis of *A. lucorum* antennae transcriptome

**Figure 1:**
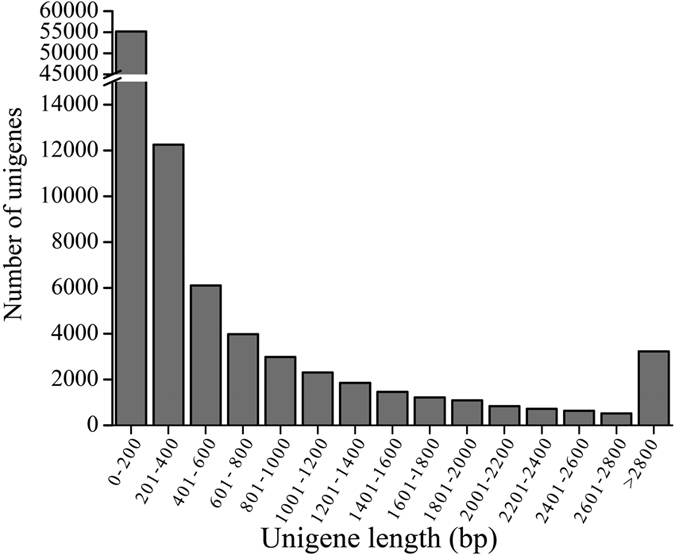
The length distribution of the assembled unigenes from *A. lucorum* male and female antennal transcriptome.

To identify candidate OR genes from *A. lucorum*, two transcriptomes of the male and female antennae were generated by HiSeq 2500 platform. A total of 88,020,104 (length between 150 to 200 bp) and 84,676,900 raw reads (length between 150 to 200 bp) were produced from the female and male antennae samples, and after filtering, 85,592,106 and 82,394,110 clean reads were assembled into 73,247 (mean length 829 bp) and 75,881 (mean length 808 bp) unigenes, respectively. The assembly of all clean reads together led to the generation of 133,447 transcripts with a mean length of 842 bp. After merging and clustering, 94,321 unigenes with a mean length of 698 bp and N50 of 1288 bp were acquired (Table 1), and 16,821 unigenes were larger than 1,000 bp in length, which comprised 17.80% of all unigenes (Fig. 1).

**Figure 2:**
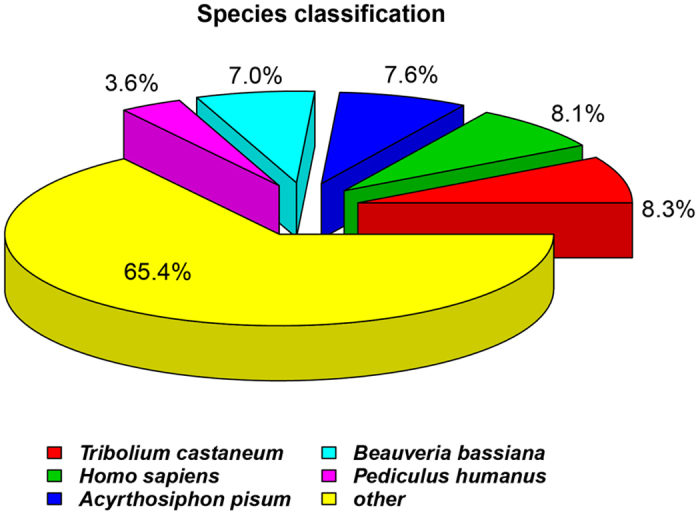
Species distribution of unigenes’ best-hit annotation term in nr database.

BLASTx and BLASTn homology searches of all 94,321 unigenes with an E-value <1.0E-5 showed that 33,076 unigenes (35.06%) had BLASTx hits in the Nr databases and 12,319 (13.06%) had BLASTn hits in the Nt databases. Among the annotated unigenes, the highest number of hits included 2,730 unigenes that were homologous to *Tribolium castaneum* sequences, and the distribution of the other best match species is shown in Fig. 2.

**Figure 3:**
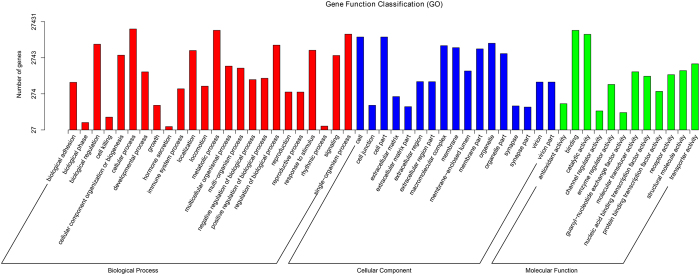
Gene ontology classifications of the *A. lucorum* unigenes.

GO assignments were used to functionally classify the predicted proteins. Of all the unigenes, 27,431 (29.08%) could be classified into three functional categories: molecular function, biological process, and cellular component (Fig. 3). In molecular function category, the genes expressed in the antennae were mostly linked to binding (15,728/44.89% unigenes) and catalytic activity (12,187/34.78% unigenes). In terms of the biological process, the most represented biological processes were cellular processes (17,197/21.23% unigenes), metabolic processes (15,800/19.51% unigenes), and single-organism process (12,343/15.24% unigenes). In the cellular component terms, cell (10,304/20.06% unigenes) and cell part (10,304/20.06% unigenes) constitute the most abundant categories (Fig. 3).

### Identification of candidate ORs

A total of 110 candidate OR genes with amino acid sequences homology to known insect ORs were identified based on the antennal transcriptome data analysis of *A. lucorum*, among which 46 sequences encoded a full-length ORF with length ranging from 374 to 471 amino acids (Table 2). Five previously described ORs (*AlucOR30, AlucOR18, AlucOR12, AlucOR28*, and *AlucOrco*) with completed ORF were identified again in our dataset with a high level of identity (95–100%)^18^,^31^, and the remaining OR genes were named as “AlucORx” (x = 1–11, 13–17, 19–27, 29, 30–109), which was consistent with the general naming of OR genes. All the full length OR genes showed 3–8 predicted transmembrane domains and the majority of incomplete OR genes also showed multiple transmembrane domains (Table 2), which is a typical characteristic of insect OR genes, indicating that these proteins were located in the membrane of the neuron cells. Except for *AlucOR25* and *AlucOR88* exhibiting a high degree of similarity (67.9%), all the other candidate *AlucOR*s were highly divergent sharing relatively low amino acid identities (19–48%) (Supplementary Table 1). In addition, all the candidate *AlucOR*s also had relative low amino acid identities (19–63%) with the homologous ORs in other species according to the BLASTx results of NCBI (Table 2).

### Phylogenetic analysis

**Figure 4:**
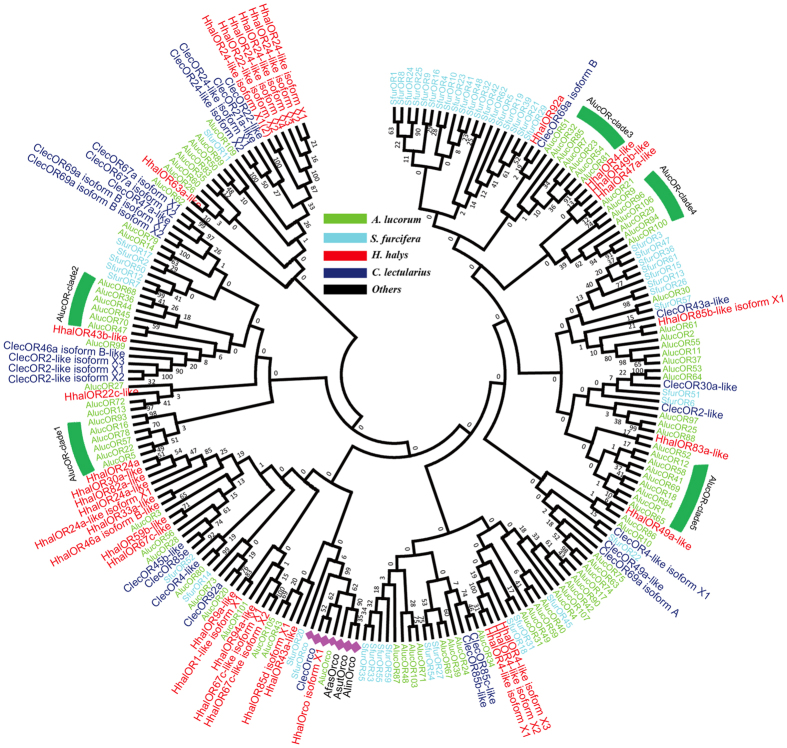
Neighbor-joining tree of candidate OR proteins (>200 aa) from Hemiptera species. The evolutionary distances were computed using the p-distance method. Afas: *Adelphocoris fasciaticollis*; Alin: *Adelphocoris lineolatus*; Asut: *Adelphocoris suturalis*; Aluc: *Apolygus lucorum*; Hhal: *Halyomorpha halys*; Clec: *Cimex lectularius*; Sfur: *Sogatella furcifera*.

In phylogenetic tree, Orco from seven Hemipteran species were easily assigned to one branch because of sharing high similarity (Fig. 4). By contrast, the other ORs are relatively divergent and formed several monophyletic clades (Fig. 4). Several species-specific subgroups were formed such as AlucOR-clade 1 to AlucOR-clade 5, indicating their closely orthologous relationship and specie-specific functions. In addition, several other AlucORs did not cluster in species-specific clades, like AlucOR30 clustered with SfurORs, AlucOR101 and AlucOR3 clustered with HhalORs, and AlucOR109 clustered with ClecORs, suggesting that some Hemipteran ORs may have common basic functions.

### Transcript expressions of *AlucORs*

Based on RPKM value analysis of the 110 *AlucORs*, we found that *AlucOrco* was the most abundant expressed gene (RPKM >170) in antennae, followed by *AlucOR104* (RPKM >53), *AlucOR18* (RPKM >37), and *AlucOR41* (RPKM >35) (Supplementary Table 2).

**Figure 5:**
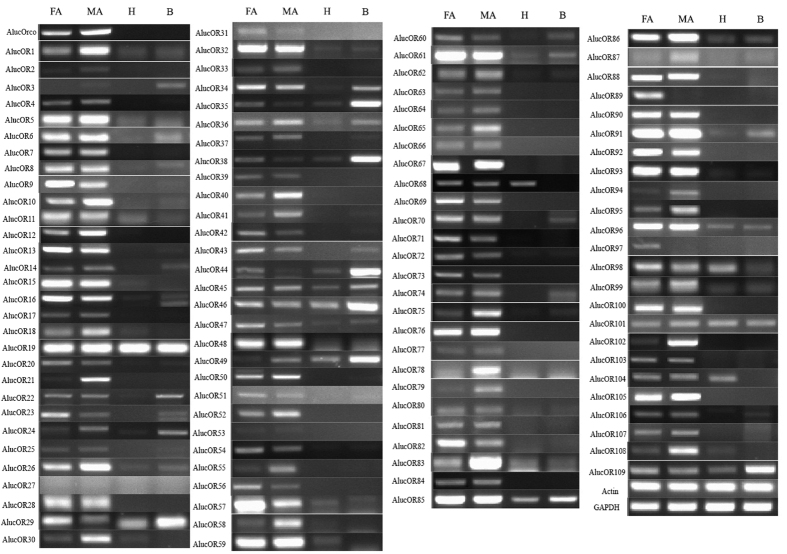
Tissues expression profiles of candidate *AlucORs* evaluated by RT-PCR. FA: female antennae; MA: male antennae; H: heads; B: body parts without heads including body parts including thoraxes, abdomens, legs, and wings.

The expression profiles of *AlucORs* in four different tissues (female antennae, male antennae, head without antennae, and body parts without heads) were evaluated by using RT-PCR. The results revealed that *AlucORs* had distinct expression profiles. Four (*AlucOR2, AlucOR3, AlucOR27*, and *AlucOR53*) of the 109 *AlucOR* genes showed very weak or undetectable expression levels in both male and female antennae (Fig. 5). *AlucOR89* and *AlucOR97* were uniquely expressed in the female antennae, while *AlucOR21* and *AlucOR78* were uniquely expressed in the male antennae (Fig. 5). Ten *AlucORs (AlucOR23, AlucOR29, AlucOR31, AlucOR35, AlucOR38, AlucOR42, AlucOR44, AlucOR60, AlucOR82*, and *AlucOR95*) were higher expressed in the female antennae than in male antennae. Eighteen *AlucORs (AlucOR1, AlucOR12, AlucOR18, AlucOR24, AlucOR30, AlucOR40, AlucOR41, AlucOR49, AlucOR55, AlucOR58, AlucOR65, AlucOR75, AlucOR79, AlucOR83, AlucOR87, AlucOR94, AlucOR102*, and *AlucOR108*) were higher expressed in the male antennae than in female antennae. Thirteen *AlucORs (AlucOR19, AlucOR29, AlucOR44, AlucOR45, AlucOR46, AlucOR49, AlucOR68, AlucOR85, AlucOR96, AlucOR98, AlucOR101, AlucOR104*, and *AlucOR109*) could be detected highly expressed in the head and 12 *AlucORs (AlucOR19, AlucOR22, AlucOR24, AlucOR29, AlucOR34, AlucOR35, AlucOR38, AlucOR44, AlucOR45, AlucOR46, AlucOR85*, and *AlucOR101*) were abundant in the tissue of body parts. However, the remaining *AlucORs* appeared to be predominantly expressed in both the female and male antennae with similar expression levels (Fig. 5).

In order to further investigate the *AlucORs* transcript profile in detail, qPCR analysis was performed to measure relative expression levels of the 110 *AlucOR* genes in seven different tissue samples (female antennae, male antennae, heads without antennae, thoraxes, abdomens, legs and wings). The results revealed the expression levels of 40 *AlucOR* genes were significantly higher abundant (more than five times) in the antennae than in other body parts (Fig. S3), among which five OR genes (*AlucOR2, AlucOR53, AlucOR96, AlucOR24, AlucOR100*) showed similar expressions between the sexes. The expression level of *AlucOR91* in female antennae was 7.4 times that of in other tissues, and nine *AlucORs (AlucOR4, AlucOR13, AlucOR14, AlucOR21, AlucOR65, AlucOR71, AlucOR81, AlucOR83*, and *AlucOR102*) in the male antennae were 5.5 to 38.1 fold higher expressed than in other tissues (Fig. S3). Comparative analysis of expression level of *AlucOR* genes between male and female antennae revealed that the expression level of 25 *AlucOR* genes in male antennae were 3 times higher than that in female antennae and seven *AlucOR* genes in female antennae were 3 times higher than that in male antennae. In addition to these antennal highly expressed *AlucOR* genes, we also identified some other tissues highly expressed OR genes, including two head highly expressed, two abdomen highly expressed and four wing highly expressed OR genes (Supplementary Table 3).

## Discussion

In this work, the repertoire of ORs in *A. lucorum* was determined by using RNA-Seq method. After extensive sequencing, assembly, and bioinformatic analysis, a total of candidate 110 OR genes were identified, including five previously annotated OR genes (*AlucOR12, AlucOR18, AlucOR28, AlucOR30*, and *AlucOrco*). Subsequent cloning and sequencing of these OR sequences with specific primers showed that our transcriptome data was highly credible. Compared to other Hemipteran transcriptomes of *Sogatella furcifera* with 63ORs^32^, *Aphis gossypii* with 45ORs^33^, and *Acyrthosiphon pisum* with 73ORs^34^, our OR dataset of 110 sequences showed an expansion of *AlucOR* family, which could provide the diversity of odorant receptors that allowed *A. lucorum* to recognize diverse odors. Indeed, *A. lucorum* feed on a wide range of plants that emit complex and species specific volatiles^26^. Nevertheless, because the ORF of some ORs is incomplete, we cannot exclude the possibility that some of these ORs might be pseudogenes. The sequence number is much lower compared with species including *Apis mellifera* (163 ORs)^35^, *Tribolium castaneum* (341ORs)^36^, and *Locusta migratoria* (142 ORs)^37^. This may be caused by adaptation of distinct species to their hosts during evolution^38^.

For a better understanding the function of these *AlucOR* genes, tissue-specific expressions were evaluated by using RT-PCR and qPCR methods. Results showed that *AlucOR* genes exhibited diverse expression patterns, which could be briefly classified into five types: antennal highly expressed ORs, head highly expressed ORs, abdomen highly expressed ORs, wing highly expressed ORs, and broadly expressed ORs (Supplementary Table 2). It was also reported that some ORs could be expressed in a variety of tissues apart from the olfactory organs^37^,^39^. Antennae are important sensory organs for insect, so the majority of *AlucOR* genes displayed high expressions in antennae, including female and male antennal highly expressed genes. Five *AlucORs (AlucOR2, AlucOR5, AlucOR24, AlucOR96*, and *AlucOR100*) were predominantly expressed in the antennae of males and females with similar expression levels, which suggested that these ORs could play important roles in the detection of general odorants, such as host plant volatiles. In particular, we found that *AlucOR91* were highly expressed in the female antennae (7.4 times higher than in other tissues) and *AlucOR21* in the male antennae (38.1 times higher than in other tissues), indicating sex-specific functions of *AlucOR91* and *AlucOR21*. According to previous studies of the insect OR functions in moths^40^,^41^,^42^,^43^,^44^, the male-dominant expression of ORs might be involved in the detection and discrimination of the sex pheromone or in other male-specific behaviors, while female-dominant expression of ORs might have the preferential function that is critical to female olfactory behavior, such as oviposition sites selection or male-produced courtship pheromones detection. The sex-specific functions of these ORs need to be further investigated in the future. In addition, we found eight *AlucOR* genes were highly expressed in heads, legs, or wings rather than antennae. The expression of broadly expressed OR genes in non-olfactory tissues suggested that they might have diverse physiological functions in other organs. The co-expression of *LmigOR95* and *LmigOrco* were also observed in the fat body of migratory locust^37^. In locusts and mosquitoes, the testis-enhanced OR genes supposed to participate in the sperm chemotaxis, fertilization, or the activation of spermatozoa^37^,^45^. Further research on the broadly expressed OR genes is worthwhile to elucidate their roles in the non-olfactory tissues.

The phylogenetic analysis of 207 ORs (>200 aa) from seven Hemiptera species demonstrated that these OR genes had undergone functional differentiation due to their scattered distribution. Except for Orco, the sequences of other OR genes were differentiated into several different clades even within the conspecifics (Fig. 4), which is consistent with the previous research^11^,^32^,^33^. In particular, despite the diversity of OR genes, many species-specific sub-clades were clustered, such as AlucOR-clade 1 to AlucOR-clade 5 (Fig. 4), suggesting a relatively conservative of these ORs within the conspecifics. The phylogenetic tree of *AlucORs* (>300 aa) were also constructed, and three large lineage-specific clades were generated, including clade 1 (33 ORs), clade 2 (32 ORs), and clade 3 (21 ORs) (Fig. S1). In particular, four male antennae highly expressed ORs (*AlucOR4, AlucOR21, AlucOR65, and AlucOR83*) were clustered into sub-clade 1 (Fig. S1). Based on previous study on sex pheromone receptor of Lepidoptera insects^42^,^43^,^44^,^45^,^46^, we speculated that the sub-clade 1 could be a cluster of potential sex pheromone receptors of *A. lucorum*.

In conclusion, based on the transcriptome analysis of male and female antennae from *A. lucorum*, an extensive set of 110 candidate *AlucOR* genes that may be related to odorant perception were identified in our laboratory. As a crucial first step toward understanding their functions, a comprehensive examination of the expression patterns of these *AlucOR* genes in different tissue samples were prefromed by using RT-PCR and qPCR. Forty ORs were found to be significantly higher abundant in antenna. One female antennae specific and nine male antennae specific *AlucOR* genes were identified successfully. The phylogenetic relationships between *AlucORs* and other Hemipteran ORs were also evaluated. The results of this study will provide a valuable foundation for further elucidating the mechanisms of olfaction in *A. lucorum*, which also could help us use odorant receptors as targets to regulate insect olfactory behavior and broaden the applications of available tools for effective control of insect pests.

## Materials and Methods

### Insect rearing

*A. lucorum* nymphs and adults were originally collected from a cotton fields at the Langfang Experimental Station of Chinese Academy of Agricultural Sciences, Hebei Province (Latitude 39.53°N, Longitude 116.70°E), China. A laboratory colony feeding on green bean pods (*Phaseolus Vulgaris* L.) was cultivated in climatic chambers under a condition of 29 ± 1 °C, relative humidity (RH) 60 ± 5% and 14:10 light: dark (L:D) photoperiod^47^.

### Sample collection

For transcriptome analysis, approximately 500 pairs of adult antennae from each sex were individually dissected and immediately immersed in liquid nitrogen, then stored at −80 °C till to the RNA isolation. For RT-PCR and qPCR analysis, different tissue samples including 500 pairs of female antennae, 500 pairs of male antennae, 200 heads without antennae, 100 thoraxes, 50 abdomens, 500 legs, 500 wings, and 50 body parts without heads were collected separately and immediately frozen in liquid nitrogen, then stored at −80 °C. Unless stated, 3–4 d old adult bugs were used in this work. All the tissue samples used for RT-PCR and qPCR were prepared in triplicate.

### RAN isolation, cDNA library construction and Illumina sequencing

Total RNA was isolated from male and female antennae by using TRIzol reagent (Invitrogen, Carlsbad, CA, USA) following the manufacturer’s instructions. The concentration, quality, and quantity of RNA samples were determined with NanoDrop ND-2000 Spectrophotometer (Nanodrop Technologies, Wilmington, DE, USA) and Qubit^®^ RNA Assay Kit in Qubit^®^ 2.0 Flurometer (Life Technologies, CA, USA). RNA integrity was assessed using the RNA Nano 6000 Assay Kit of the Bioanalyzer 2100 system (Agilent Technologies, CA, USA). The cDNA library construction and Illumina sequencing of the RNA samples were performed by Novogene Bioinformatics Technology Co. Ltd, Beijing, China. Briefly, poly-A RNA was purified from 3 μg of total RNA using oligo (dT) magnetic beads and fragmented into short sequences in the fragmentation buffer. Then, random hexamer primer and M-MuLV Reverse Transcriptase (RNaseH) was used for first-strand cDNA generation, followed by synthesis of the second-strand cDNA using RNaseH and DNA polymerase I. After end repair and adaptor ligation, the library fragments were amplified by PCR and purified using the AMPure XP system (Beckman Coulter, Beverly, USA) to obtain a cDNA library. The clustering of the index-coded samples was performed on a cBot Cluster Generation System using a TruSeq PE Cluster Kit v3-cBot-HS (Illumina) according to the manufacturer’s instructions. After cluster generation, the library preparations were sequenced on an Illumina Hiseq 2500 platform and paired-end reads were generated.

### *De novo* assembly and functional annotation

After removing short or low quality and adaptor sequence, each clean-read dataset of male and female antennae was assembled using the short read assembling program Trinity (r20140413p1) with min_kmer_cov set to 2 and all other parameters set default^48^. The resulting unigenes were further clustered by TGICL to remove redundant fragments and to acquire non-redundant unigenes as long as possible^49^. To annotate these unigenes, BLASTx search was performed against protein database of Nr, SwissProt, GO and COG (e - value < 10^−5^). The blast results were then imported into Blast2GO pipeline for Go annotations^50^.

### Transcript abundance analysis of unigenes

The transcript abundance of these unigenes were calculated based on the reads per kilobase per million mapped reads (RPKM) method^51^, using the formula: RPKM (A) = (10,00,000 × C × 1000)/(N × L), where RPKM (A) is the abundance of gene A, C is the number of reads that uniquely aligned to gene A, N is the total number of reads that uniquely aligned to all genes, and L is the number of bases in gene A. The RPKM method was able to eliminate the influence of different gene lengths and sequencing discrepancies in the calculation of expression abundance.

### Verification of OR sequences by cloning and sequencing

To obtain a more reliable sequence, all the *A. lucorum* OR sequences from transcriptome were further confirmed by gene cloning and sequencing. Gene-specific primers amplifying the intact ORF or partial sequences of each OR gene were designed by using Primer Premier 5 software (PREMIER Biosoft International, CA, USA) based on the transcriptome sequences (Supplementary Table 4). PCR reactions were carried out in a total reaction volume of 30 μl with template cDNA of 200 ng and Takara *LA Taq* Polymerase (TaKaRa, Dalian, China) of 0.3 μl. The PCR cycling profile was: 95 °C for 1 min, followed by 40 cycles of 95 °C for 20 sec, 57 °C for 20 sec, 72 °C for 1 min, and a final extension at 72 °C for 10 min. The PCR products were subsequently gel-purified and cloned into pCloneEZ vector (CloneSmarter Technologies Inc., USA) and then sequenced with standard M13 primers.

### Sequence and phylogenetic analysis of candidate OR genes

Candidate OR gene fragments were determined by searching for homology using Blastx and Blastn tool in NCBI. The longest open reading frame (ORF) of each unigene was identified by ORF Finder (http://www.ncbi.nlm.nih.gov/gorf/gorf.html). Transmembrane domains of OR genes were predicted with the TMHMM Server Version 2.0 (http://www.cbs.dtu.dk/services/TMHMM).

Alignments of amino acid sequences were performed using the program ClustalW and further edited using Jalview 2.7^52^. A neighbor-joining tree was constructed by MEGA 6.06 based on p-distance model^53^. The bootstrap support of tree branches was assessed by re-sampling amino acid positions 1000 times. Phylogenetic analysis was performed with 207 ORs (>200 aa) from seven Hemipteran species. The dataset contains 92 ORs from *Apolygus lucorum*, 36 ORs from *Halyomorpha halys*, 27 ORs from *Cimex lectularius*, 49 ORs from *Sogatella furcifera*^32^, and three Orco genes from *Adelphocoris fasciaticollis, Adelphocoris lineolatus*, and *Adelphocoris suturalis*. The protein sequences of the 207 ORs used in this analysis are listed in Supplementary Table 5. In addition, the phylogenetic tree was constructed with 86 selected *AlucORs* (>300 aa).

### RT-PCR analysis

The expression of *AlucOR* transcripts in different tissues (female antennae, male antennae, heads without antennae, and body parts without heads) were evaluated by RT-PCR. Total RNA was extracted from tissue samples by using Trizol reagent following the manufacturer’s instructions. RNA concentration and quality were checked by Nanodrop ND-2000 spectrophotometer and 1.1% agarose gel electrophoresis. For each sample, 2 μg of total RNA was used for cDNA synthesis in a total reaction volume of 20 μl by using FastQuant RT Kit (with gDNase, Tiangen Biotech, Beijing Co., Ltd.) according to the manufacturer’s protocol. The *β-actin* (GenBank accession number: JN616391.1) and *GAPDH* (GenBank accession number: JX987672.1) of *A. lucorum* were selected as the control genes to assess the cDNA integrity. The specific primers of target and control genes in RT-PCR were designed by using Primer Premier 5 software (Supplementary Table 6). An equal amount of cDNA (200 ng) was added to each reaction mixture (50 μl) under the following cycling conditions: 94 °C for 4 min, followed by 35 cycles of 3-step amplification of 94 °C for 30 s, 55~60 °C for 30 s, 72 °C for 50 s, and a final extension for 10 min at 72 °C. PCR products were checked on a 1% agarose gel and verified by DNA sequencing. Three repeats with three biological samples of each gene were performed in this experiment.

### qPCR measurment

The relative expression levels of target *AlucOR* genes in seven different tissues (female antennae, male antennae, heads without antennae, thoraxes, abdomens, legs, and wings) were further examined by qPCR on an ABI Prism 7500 system (Applied Biosystems, Carlsbad, CA, USA) using a mixture of 10 μl 2 × SuperReal PreMix Plus (Tiangen Biotech, Beijing Co., Ltd.), 0.8 μl of each primer (10 μM), 200 ng of sample cDNA, 0.4 μl of 50 × ROX Reference Dye and 6 μl of sterilized ultrapure water. The reaction program was composed of 95 °C for 15 min, 40 cycles of 95 °C for 10 s and 60 °C for 32 s. All the primers used in qPCR were designed with Beacon Designer 7.9 software (PREMIER Biosoft International, CA, SA) and listed in Supplementary Table 7. A discrete amplification peak and a subsequent melting curve was checked to ensure the primer specificity. A high amplification efficiency of each primer pair was calculated by a five-fold cDNA dilution series. Negative controls without template were run in parallel for each primer pair. Each reaction was performed with three biological replicates, and each biological replicate was assessed three times. Prior to qPCR, we performed semi-quantitative RT-PCR and confirmed that *β*-actin is expressed at similar level in different tissues (Fig. S2), and the expression level of each *AlucOR* gene relative to *β*-actin were calculated by using the comparative 2^−ΔΔCT^ method^54^. All data were statistically analyzed by using SAS 9.2^®^ Software (SAS Institute Inc., Carey, North Carolina, USA). One-way analysis of variance (ANOVA) followed by a Tukey’s honestly significant difference (HSD) test (*P *< 0.05) were used to compare expression of each target gene among various tissues.

## Additional Information

**How to cite this article**: An, X.-K. *et al*. Identification and expression analysis of an olfactory receptor gene family in green plant bug *Apolygus lucorum* (Meyer-Dür). *Sci. Rep.* **6**, 37870; doi: 10.1038/srep37870 (2016).

**Publisher's note:** Springer Nature remains neutral with regard to jurisdictional claims in published maps and institutional affiliations.

## Supplementary Material

Supplementary Information

## Figures and Tables

**Table 1:** Summary of antennal transcriptome assembley.

Statistics proect	Transcripts	Unigenes
Minimum length	201 bp	201 bp
Mean length	842 bp	698 bp
Median length	404 bp	334 bp
Max length	26,290 bp	26,290 bp
N50	1,579 bp	1,288 bp
N90	297 bp	256 bp
Total Nucleotides	112,413,388	65,824,946
total number	133,447	94,321

**Table 2:** List of odorant receptor genes in *A. lucorum* antennal transcriptome.

Gene name	Accesion number	ORF (aa)	Completeness	TM (No.)	Accesion number	Discription	Species	Identity (%)	E-value	Score
AlucOR1	KU958180	285	5′ lost	4	gb|AKS44360.1	olfactory receptor 12	*Apolygus lucorum*	37	9.53E-53	189
AlucOR2	KU958181	403	Complete	6	ref|XP_014293859.1	odorant receptor 85b-like isoform X1	*Halyomorpha halys*	23	6.91E-25	114
AlucOR3	KU958182	426	Complete	3	gb|AKS44362.1	olfactory receptor 28	*Apolygus lucorum*	32	2.07E-64	224
AlucOR4	KU958183	440	Complete	6	ref|XP_014271039.1	odorant receptor 83a-like	*Halyomorpha halys*	26	2.65E-23	110
AlucOR5	KU958184	429	5′ lost	5	gb|AKI29040.1	odorant receptor 49b-2	*Bactrocera dorsalis*	29	2.63E-05	57
AlucOR6	KU958185	113	5′ and 3′ lost	2	gb|ALD51489.1	odorant receptor 121	*Locusta migratoria*	33	1.9	37
AlucOR7	KU958186	367	5′ lost	5	ref|XP_014287492.1	odorant receptor 4-like	*Halyomorpha halys*	22	8.17E-10	71
AlucOR8	KU958187	99	5′ and 3′ lost	0	gb|AKS44362.1	olfactory receptor 28	*Apolygus lucorum*	63	1.23E-32	128
AlucOR9	KU958188	397	Complete	6	ref|XP_014274444.1	odorant receptor 47a-like	*Halyomorpha halys*	24	2.63E-10	72
AlucOR10	KU958189	378	Complete	3	ref|XP_003689890.2|	odorant receptor Or1-like	*Apis florea*	24	0.025	47
AlucOR11	KU958190	398	5′ lost	6	ref|XP_014250665.1	uncharacterized protein LOC106667308	*Cimex lectularius*	26	1.07E-26	120
AlucOR13	KU958192	438	Complete	6	gb|AKS44360.1	olfactory receptor 12	*Apolygus lucorum*	30	2.67E-47	179
AlucOR14	KU958193	375	Complete	4	ref|XP_014258533.1	odorant receptor 47a-like	*Cimex lectularius*	31	2.68E-20	100
AlucOR15	KU958194	107	5′ lost	1	ref|XP_014249919.1	putative odorant receptor 69a, isoform A	*Cimex lectularius*	43	1.97E-20	94
AlucOR16	KU958195	390	5′ lost	5	ref|XP_014290317.1	odorant receptor 4-like	*Halyomorpha halys*	22	1.09E-14	85
AlucOR17	KU958196	424	5′ lost	5	ref|XP_014287936.1	uncharacterized protein LOC106688133 isoform X1	*Halyomorpha halys*	30	1.13E-23	110
AlucOR19	KU958198	69	5′ lost	1	ref|XP_014278373.1	gustatory and pheromone receptor 32a-like	*Halyomorpha halys*	58	6.09E-16	79
AlucOR20	KU958199	402	5′ lost	6	ref|XP_014258779.1	putative odorant receptor 92a	*Cimex lectularius*	43	1.4E-104	327
AlucOR21	KU958200	352	5′ lost	4	ref|XP_014294765.1	odorant receptor 4-like	*Halyomorpha halys*	26	1.57E-15	87
AlucOR22	KU958201	403	Complete	6	ref|XP_001810505.1	odorant receptor Or1	*Tribolium castaneum*	25	0.000836	52
AlucOR23	KU958202	426	Complete	6	ref|XP_014287492.1	odorant receptor 4-like	*Halyomorpha halys*	26	8.84E-27	120
AlucOR24	KU958203	324	3′ lost	5	ref|XP_014256416.1	odorant receptor 46a, isoform B-like	*Cimex lectularius*	45	1.43E-84	272
AlucOR25	KU958204	430	5′ lost	6	gb|AKS44360.1	olfactory receptor 12	*Apolygus lucorum*	30	1.1E-45	175
AlucOR26	KU958205	281	3′ lost	4	ref|NP_001177519.1	odorant receptor 100	*Nasonia vitripennis*	38	2	40
AlucOR27	KU958206	392	Complete	6	ref|XP_014287044.1	uncharacterized protein LOC106687584	*Halyomorpha halys*	31	9.15E-17	91
AlucOR29	KU958207	403	Complete	5	gb|ALD51384.1	odorant receptor 61	*Locusta migratoria*	21	0.002	51
AlucOR31	KU958208	464	5′ lost	4	ref|XP_014270188.1	odorant receptor 4-like isoform X1	*Halyomorpha halys*	24	5.4E-35	145
AlucOR32	KU958209	431	Complete	6	ref|XP_014287492.1	odorant receptor 4-like	*Halyomorpha halys*	29	2.02E-41	162
AlucOR33	KU958210	96	5′ lost	0	ref|XP_011194820.1	odorant receptor Or2-like	*Bactrocera cucurbitae*	34	0.000896	47
AlucOR34	KU958211	328	5’ lost	3	ref|XP_014261896.1	odorant receptor 85c-like	*Cimex lectularius*	40	1.21E-73	244
AlucOR35	KU958212	374	5′ lost	7	ref|XP_014293240.1	gustatory and odorant receptor 63a-like	*Halyomorpha halys*	22	3.15E-11	75
AlucOR36	KU958213	387	Complete	6	ref|XP_014257038.1	odorant receptor Or2-like isoform X2	*Cimex lectularius*	31	2.48E-26	119
AlucOR37	KU958214	397	5′ lost	6	ref|XP_014293859.1	odorant receptor 85b-like isoform X1	*Halyomorpha halys*	27	7.09E-22	105
AlucOR38	KU958215	57	5′ lost	0	ref|XP_317761.2	AGAP007757-PA	*Anopheles gambiae str. PEST*	31	5.6	35
AlucOR39	KU958216	394	Complete	5	ref|XP_014254258.1	uncharacterized protein LOC106669355 isoform X2	*Cimex lectularius*	45	6.4E-97	305
AlucOR40	KU958217	437	Complete	6	ref|XP_014274444.1	odorant receptor 47a-like	*Halyomorpha halys*	23	2.86E-19	99
AlucOR41	KU958218	415	Complete	6	gb|AKS44360.1	olfactory receptor 12	*Apolygus lucorum*	30	1.84E-39	157
AlucOR42	KU958219	433	5′ lost	7	ref|XP_014289672.1	odorant receptor 49a-like	*Halyomorpha halys*	26	1.99E-17	94
AlucOR43	KU958220	420	5′ lost	7	ref|XP_014249551.1	putative odorant receptor 92a	*Cimex lectularius*	31	8.24E-55	199
AlucOR44	KU958221	393	Complete	8	ref|XP_014287040.1	odorant receptor 4-like	*Halyomorpha halys*	46	9.11E-06	58
AlucOR45	KU958222	391	Complete	6	ref|XP_014287040.1	odorant receptor 4-like	*Halyomorpha halys*	48	8.02E-16	89
AlucOR46	KU958223	95	5′ lost	0	ref|XP_014287040.1	odorant receptor 4-like	*Halyomorpha halys*	47	3.32E-19	91
AlucOR47	KU958224	337	5′ lost	5	ref|XP_014287040.1	odorant receptor 4-like	*Halyomorpha halys*	27	1.24E-26	119
AlucOR48	KU958225	374	Complete	6	ref|XP_014278976.1	uncharacterized protein LOC106682571	*Halyomorpha halys*	26	8.19E-10	71
AlucOR49	KU958226	336	5′ and 3′ lost	6	ref|XP_014258861.1	uncharacterized protein LOC106672172	*Cimex lectularius*	25	9.02E-16	87
AlucOR50	KU958227	413	Complete	6	ref|XP_014256564.1	uncharacterized protein LOC106670594 isoform X1	*Cimex lectularius*	26	3.24E-38	152
AlucOR51	KU958228	406	5′ lost	6	ref|XP_014287492.1	odorant receptor 4-like	*Halyomorpha halys*	29	6.66E-42	162
AlucOR52	KU958229	437	Complete	6	ref|XP_014273407.1	uncharacterized protein LOC106679017 isoform X1	*Halyomorpha halys*	25	1.38E-24	114
AlucOR53	KU958230	434	Complete	6	gb|AKS44361.1	olfactory receptor 18	*Apolygus lucorum*	31	1.81E-49	185
AlucOR54	KU958231	411	Complete	5	ref|XP_014270190.1	odorant receptor 4-like isoform X3	*Halyomorpha halys*	23	2.18E-21	103
AlucOR55	KU958232	408	3′ lost	4	ref|XP_014250665.1	uncharacterized protein LOC106667308	*Cimex lectularius*	27	8.02E-27	120
AlucOR56	KU958233	419	Complete	6	gb|AKS44362.1	olfactory receptor 28	*Apolygus lucorum*	57	6.7E-154	454
AlucOR57	KU958234	362	5′ lost	5	gb|ALR72576.1	odorant receptor OR33	*Colaphellus bowringi*	26	5.39E-11	71
AlucOR58	KU958235	433	Complete	6	gb|AKS44360.1	olfactory receptor 12	*Apolygus lucorum*	40	2.55E-93	300
AlucOR59	KU958236	439	Complete	6	ref|XP_014287936.1	uncharacterized protein LOC106688133 isoform X1	*Halyomorpha halys*	23	7.58E-11	73
AlucOR60	KU958237	315	3′ lost	5	ref|XP_014249551.1	putative odorant receptor 92a	*Cimex lectularius*	33	1.42E-41	160
AlucOR61	KU958238	266	3′ lost	3	ref|XP_014273330.1	odorant receptor 85b-like	*Halyomorpha halys*	33	1.35E-31	131
AlucOR62	KU958239	224	5′ lost	4	gb|AKS44361.1	olfactory receptor 18	*Apolygus lucorum*	25	0.000493	51
AlucOR63	KU958240	380	Complete	5	ref|XP_014249919.1	putative odorant receptor 69a, isoform A	*Cimex lectularius*	31	7.33E-38	151
AlucOR64	KU958241	316	3′ lost	4	gb|AKS44361.1	olfactory receptor 18	*Apolygus lucorum*	23	1.12E-17	93
AlucOR65	KU958242	449	5′ lost	7	ref|XP_014273407.1	uncharacterized protein LOC106679017 isoform X1	*Halyomorpha halys*	28	4.03E-28	125
AlucOR66	KU958243	88	5′ lost	0	gb|AKS44360.1	olfactory receptor 12	*Apolygus lucorum*	41	5.46E-14	76
AlucOR67	KU958244	417	Complete	5	ref|XP_014254257.1	uncharacterized protein LOC106669355 isoform X1	*Cimex lectularius*	42	1.07E-96	307
AlucOR68	KU958245	394	Complete	6	ref|XP_014287040.1	odorant receptor 4-like	*Halyomorpha halys*	46	4.98E-16	89
AlucOR69	KU958246	436	5′ and 3′ lost	6	gb|AKS44360.1	olfactory receptor 12	*Apolygus lucorum*	31	2.99E-44	171
AlucOR70	KU958247	384	Complete	7	ref|XP_014287040.1	odorant receptor 4-like	*Halyomorpha halys*	28	1.84E-42	164
AlucOR71	KU958248	376	Complete	6	ref|XP_014258590.1	odorant receptor 67a isoform X1	*Cimex lectularius*	25	1.11E-18	96
AlucOR72	KU958249	235	5′ lost	3	gb|AKS44360.1	olfactory receptor 12	*Apolygus lucorum*	30	2.77E-21	101
AlucOR73	KU958250	423	Complete	6	ref|XP_014292012.1	odorant receptor 22c-like	*Halyomorpha halys*	26	7.75E-43	166
AlucOR74	KU958251	267	3′ lost	4	ref|XP_014249919.1	putative odorant receptor 69a, isoform A	*Cimex lectularius*	26	5.22E-07	61
AlucOR75	KU958252	383	Complete	3	ref|XP_014249919.1	putative odorant receptor 69a, isoform A	*Cimex lectularius*	29	2.55E-44	169
AlucOR76	KU958253	182	5′ and 3′ lost	3	ref|XP_014250664.1	odorant receptor 43a-like	*Cimex lectularius*	32	3.66E-12	73
AlucOR77	KU958254	98	5′ lost	0	gb|ALR72557.1	odorant receptor OR12	*Colaphellus bowringi*	38	4.54E-06	52
AlucOR78	KU958255	403	5′ lost	6	gb|AJO62227.1	olfactory receptor OR8	*Tenebriomolitor*	25	1.63E-06	61
AlucOR79	KU958256	382	Complete	6	ref|XP_014258533.1	odorant receptor 47a-like	*Cimex lectularius*	19	2.71E-07	63
AlucOR80	KU958257	411	5′ lost	6	ref|XP_014289200.1	odorant receptor 82a-like	*Halyomorpha halys*	55	1.5E-133	401
AlucOR81	KU958258	426	5′ lost	4	ref|XP_014287492.1	odorant receptor 4-like	*Halyomorpha halys*	23	1.07E-24	114
AlucOR82	KU958259	105	5′ and 3′ lost	2	ref|XP_014290637.1	uncharacterized protein LOC106689927 isoform X1	*Halyomorpha halys*	33	1.38E-05	53
AlucOR83	KU958260	372	5′ and 3′ lost	6	ref|XP_014289672.1	odorant receptor 49a-like	*Halyomorpha halys*	28	0.018	47
AlucOR84	KU958261	437	5′ lost	6	gb|AKS44361.1	olfactory receptor 18	*Apolygus lucorum*	35	2.13E-72	246
AlucOR85	KU958262	434	Complete	4	ref|XP_014270188.1	odorant receptor 4-like isoform X1	*Halyomorpha halys*	29	8.44E-38	152
AlucOR86	KU958263	409	5′ lost	6	ref|XP_014250665.1	uncharacterized protein LOC106667308	*Cimex lectularius*	30	5.03E-42	163
AlucOR87	KU958264	407	5′ lost	4	ref|XP_014274899.1	uncharacterized protein LOC106679982	*Halyomorpha halys*	32	3.29E-50	186
AlucOR88	KU958265	430	5′ lost	6	gb|AKS44360.1	olfactory receptor 12	*Apolygus lucorum*	32	1.29E-49	186
AlucOR89	KU958266	406	Complete	6	ref|XP_014256564.1	uncharacterized protein LOC106670594 isoform X1	*Cimex lectularius*	26	6.79E-30	129
AlucOR90	KU958267	444	5′ lost	5	ref|XP_014289672.1	odorant receptor 49a-like	*Halyomorpha halys*	26	7.75E-19	98
AlucOR91	KU958268	74	5′ and 3′ lost	1	ref|NP_001177702.1	odorant receptor 156	*Nasonia vitripennis*	37	0.007	44
AlucOR92	KU958269	136	5′ and 3′ lost	2	gb|AKS44362.1	olfactory receptor 28	*Apolygus lucorum*	58	1.25E-35	137
AlucOR93	KU958270	393	5′ lost	5	ref|XP_014256808.1	odorant receptor 46a, isoform B-like	*Cimex lectularius*	28	2.63E-08	66
AlucOR94	KU958271	420	5′ lost	3	ref|XP_014275211.1	odorant receptor 24a-like	*Halyomorpha halys*	26	0.032	47
AlucOR95	KU958272	115	5′ lost	0	gb|AKS44361.1	olfactory receptor 18	*Apolygus lucorum*	40	6.45E-19	91
AlucOR96	KU958273	386	Complete	6	ref|XP_014294765.1	odorant receptor 4-like	*Halyomorpha halys*	24	0.071	46
AlucOR97	KU958274	432	5′ lost	7	gb|AKS44360.1	olfactory receptor 12	*Apolygus lucorum*	29	1.04E-47	180
AlucOR98	KU958275	122	5′ lost	2	gb|AKS44360.1	olfactory receptor 12	*Apolygus lucorum*	31	1.67E-09	65
AlucOR99	KU958276	390	5′ lost	6	ref|XP_014256808.1	odorant receptor 46a, isoform B-like	*Cimex lectularius*	28	1.09E-22	107
AlucOR100	KU958277	402	Complete	3	gb|ALD51419.1	odorant receptor 136	*Locusta migratoria*	26	0.13	44
AlucOR101	KU958278	378	5′ lost	4	ref|XP_014294439.1	odorant receptor Or1-like isoform X1	*Halyomorpha halys*	36	2.77E-70	237
AlucOR102	KU958279	58	5′ and 3′ lost	1	ref|XP_015594072.1	odorant receptor 13a-like isoform X3	*Cephus cinctus*	32	0.049	41
AlucOR103	KU958280	385	5′ lost	6	ref|XP_014258596.1	odorant receptor 67a isoform X4	*Cimex lectularius*	29	5.76E-16	85
AlucOR104	KU958281	178	5′ lost	1	ref|XP_014290637.1	uncharacterized protein LOC106689927 isoform X1	*Halyomorpha halys*	28	1.58E-10	69
AlucOR105	KU958282	421	5′ lost	6	ref|XP_014249551.1	putative odorant receptor 92a	*Cimex lectularius*	33	4.86E-66	228
AlucOR106	KU958283	396	Complete	5	ref|XP_014294765.1	odorant receptor 4-like	*Halyomorpha halys*	26	1.68E-12	79
AlucOR107	KU958284	435	Complete	6	ref|XP_014273407.1	uncharacterized protein LOC106679017 isoform X1	*Halyomorpha halys*	27	4.99E-26	119
AlucOR108	KU958191	144	5′ lost	2	ref|XP_014293240.1	gustatory and odorant receptor 63a-like	*Halyomorpha halys*	38	1.46E-09	66
AlucOR109	KU958197	418	Complete	7	ref|XP_014244354.1	gustatory and odorant receptor 22-like	*Cimex lectularius*	50	6.5E-126	382
