# Supplementary material for: Identification and expression analysis of an olfactory receptor gene family in green plant bug Apolygus lucorum (Meyer-Dür)
Source: Sci Rep. 2016 Nov 28;6:37870. doi: 10.1038/srep37870 (PMC5124970; doi:10.1038/srep37870)
Supplement: Supplementary Information [file srep37870-s1.pdf]

Supplementary Information for

Identification and Expression Analysis of an Olfactory Receptor Gene Family in green plant bug *Apolygus lucorum* (Meyer-Dur)

Xing-Kui An[1], Liang Sun[1,2], Hang-Wei Liu[1], Dan-Feng Liu[1], Yu-Xiao Ding[1,3], Le-Mei, Li[1], Yong-Jun Zhang[1,*], Yu-Yuan Guo[1]

[1] State Key Laboratory for Biology of Plant Diseases and Insect Pests, Institute of Plant Protection, Chinese Academy of Agricultural Sciences, Beijing, 100193, China.

[2] Key Laboratory of Tea Biology and Resources Utilization, Ministry of Agriculture, Tea Research Institute, Chinese Academy of Agricultural Sciences, Hangzhou, 310008, China.

[3] College of Agronomy, Jilin Agricultural University, Changchun, Jilin 130118, China.

* Corresponding author

Yong-Jun Zhang, PhD. State Key Laboratory for Biology of Plant Diseases and Insect Pests, Institute of Plant Protection, Chinese Academy of Agricultural Sciences, Beijing, 100193, China.

Email: yjzhang@ippcaas.cn; Tel.:+86 10 62815929; Fax: +86 10 62894786.

Supplementary Figures

Supplementary Figure S1. Neighbor-joining tree of candidate OR proteins (> 300 aa) from *A. lucorum*. The evolutionary distances were computed using the *p*-distance method. Genes that are uniquely or primarily expressed in the male antennae were labeled with a black square and sub-clade 1 could be a cluster of potential sex pheromone receptors of *A. lucorum*.

Supplementary Figure S2.

The expression level of *β*-actin in different tissues. The expression level of *β*-actin in different tissues was validated by semi-quantitative RT-PCR. F: Female antennae; M: Male antennae; H: Heads without antennae; T: Thoraxes; Ab: Abdomens; L: Legs; W: Wings; B: body parts without antennae and head.

Supplementary Figure S3.

The qPCR anaysis of candidate *AlucORs*. FA: female antennae; MA: male antennae; H: heads; T: thoraxes; A: abdomens; L: legs; W: wings. The *β*-actin was used as an internal reference gene to normalize transcript levels in each sample. The standard error is represented by the error bar, and the different letters (a, b, c) above each bar denote significant differences ($P < 0.05$).

Supplementary Tables

Supplementary Table 1.

Comparison of *A. lucorum* OR proteins sequences. A percent identity matrix of 110 AlucORs was calculated by BioEdit Sequence Alignment Editor. The percentage of identity of each pair is shown.

Supplementary Table 2.

Rankings of RPKM value of all the *AlucORs* obtained from transcriptome analysis.

Supplementary Table 3.

The relative expression level of *AlucORs* including antennal highly expressed *AlucORs*, antennal highly expressed *AlucORs* with a similar expression level between the sexes, female antennal highly expressed *AlucORs*, male antennal highly expressed *AlucORs*, head highly expressed *AlucORs* genes, abdomen highly expressed *AlucORs*, and wing highly expressed *AlucORs* were listed and ranked.

Supplementary Table 4.

Primers used for intact ORF or partial sequences validation of each *AlucOR*.

Supplementary Table 5.

The protein names and sequences of the 207 Hemipteran ORs used in Figure 4.

Supplementary Table 6.

Primers used for RT-PCR.

Supplementary Table 7.

Primers used in real-time PCR for determination expression level of *AlucORs*.

Supplementary Figure S1.

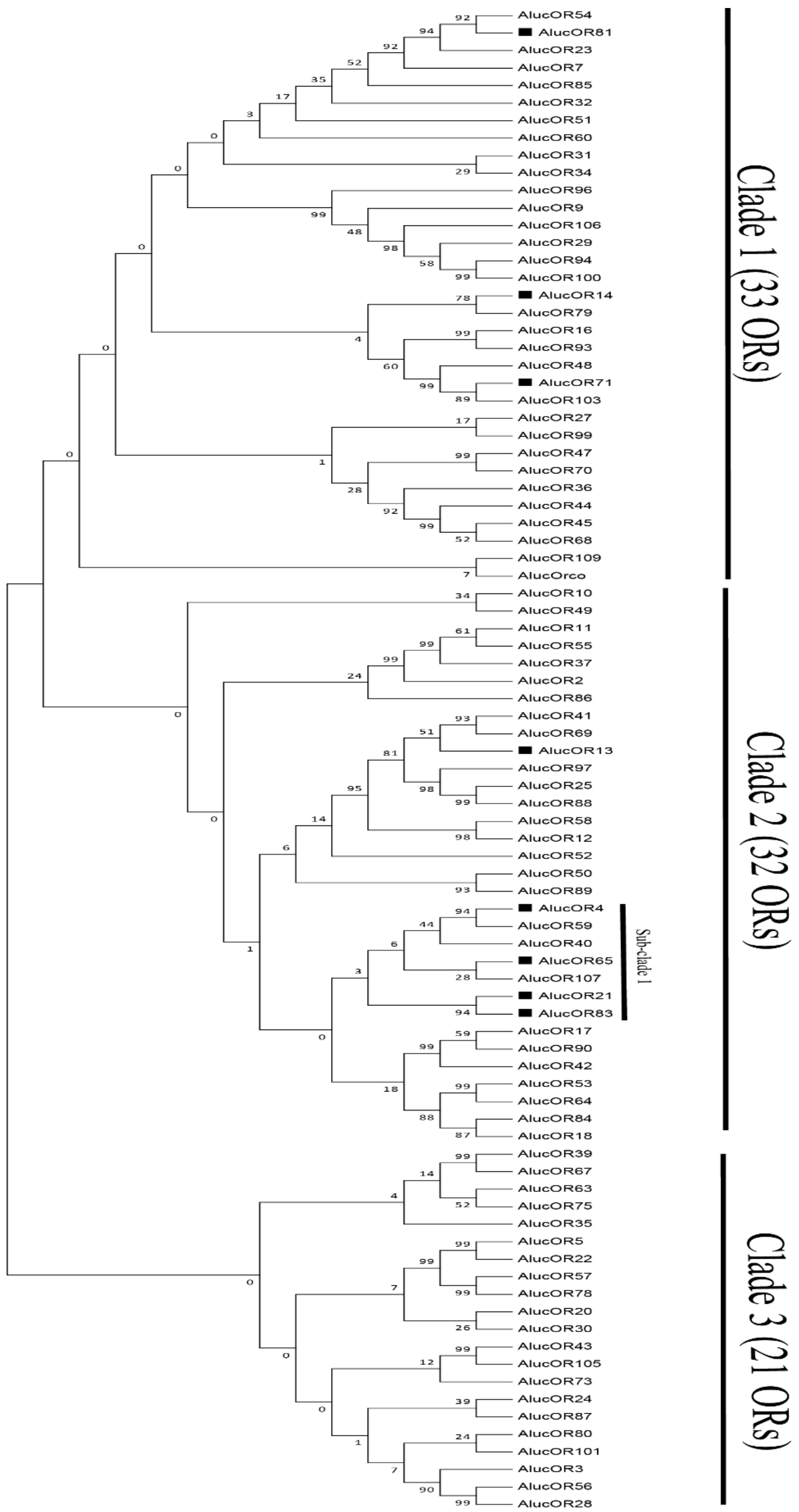

Supplementary Figure S2.

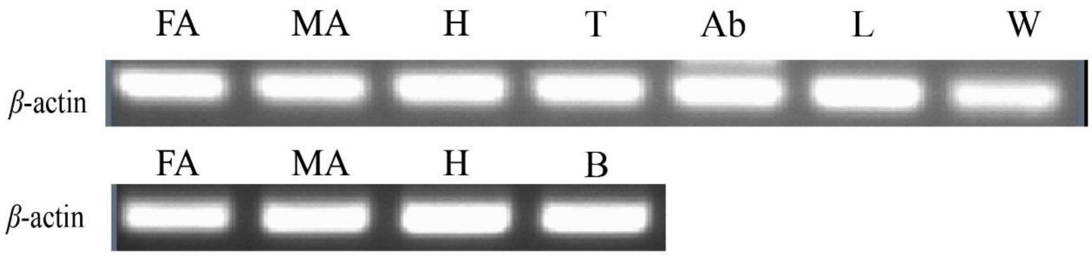
FA
MA
H
T
Ab
L
W
β-actin
FA
MA
H
B
β-actin

Supplementary Figure S3.

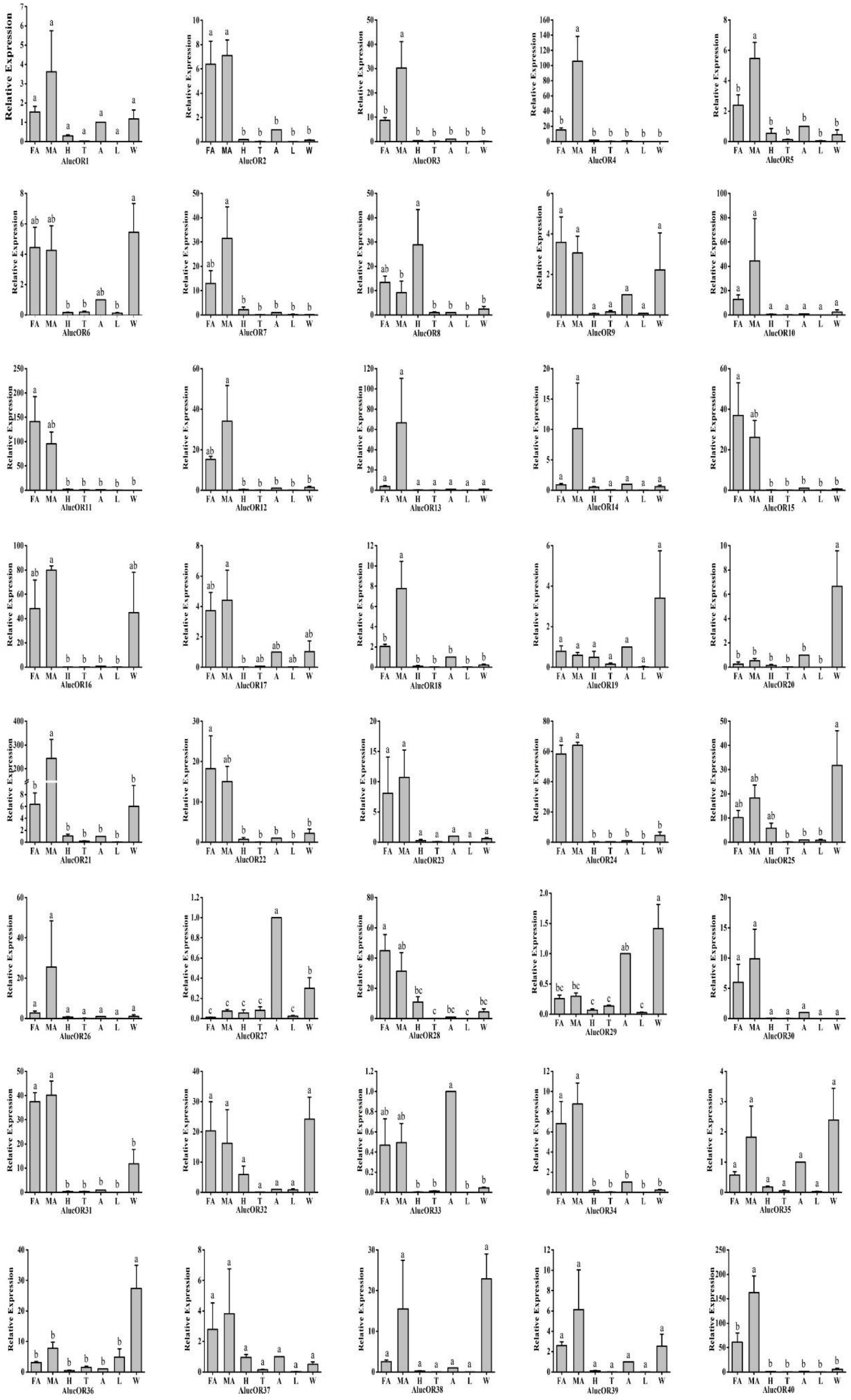

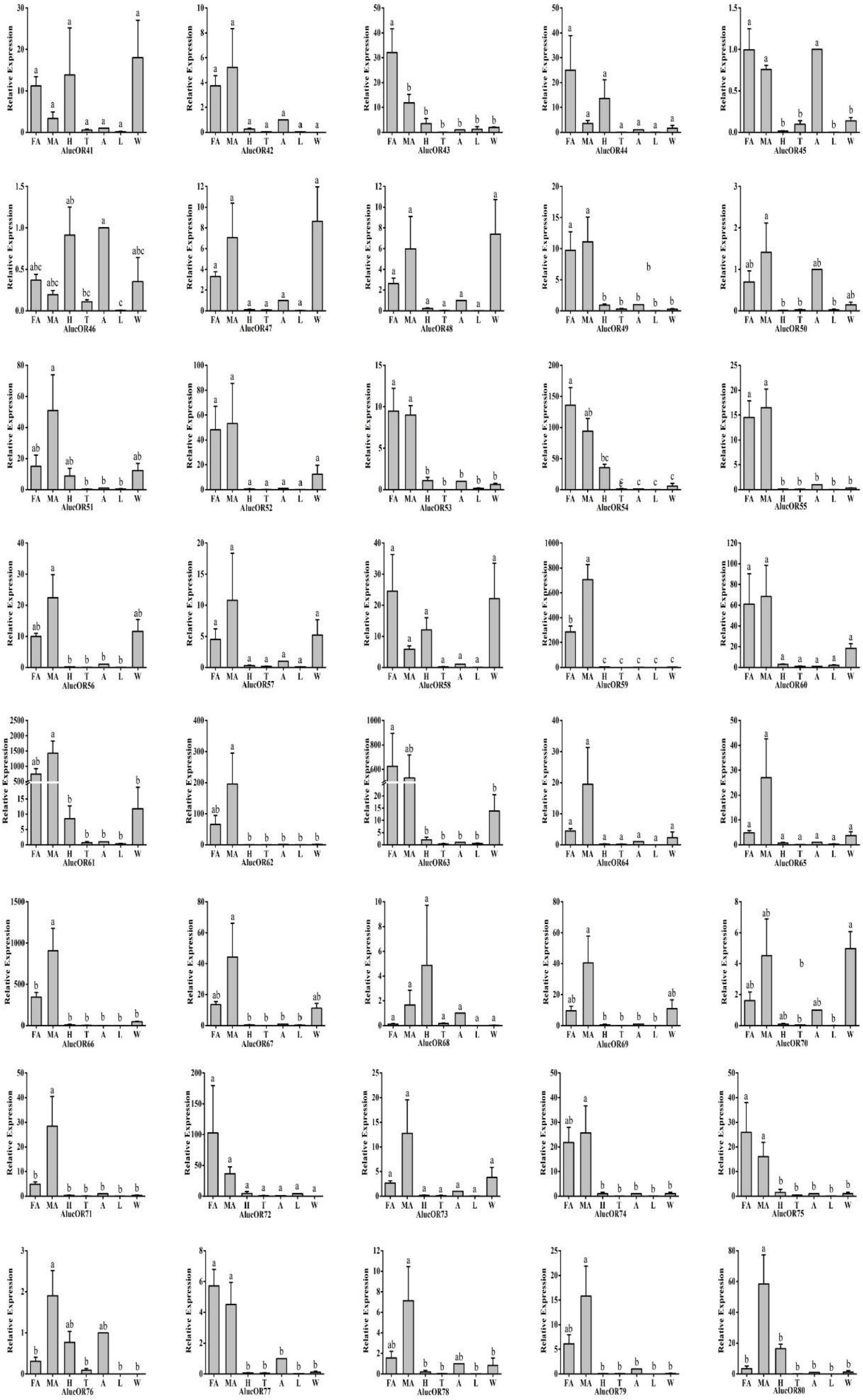
Relative Expression
FA MA H T A L W
AlucOR41
AlucOR42
AlucOR43
AlucOR44
AlucOR45
AlucOR46
AlucOR47
AlucOR48
AlucOR49
AlucOR50
AlucOR51
AlucOR52
AlucOR53
AlucOR54
AlucOR55
AlucOR56
AlucOR57
AlucOR58
AlucOR59
AlucOR60
AlucOR61
AlucOR62
AlucOR63
AlucOR64
AlucOR65
AlucOR66
AlucOR67
AlucOR68
AlucOR69
AlucOR70
AlucOR71
AlucOR72
AlucOR73
AlucOR74
AlucOR75
AlucOR76
AlucOR77
AlucOR78
AlucOR79
AlucOR80

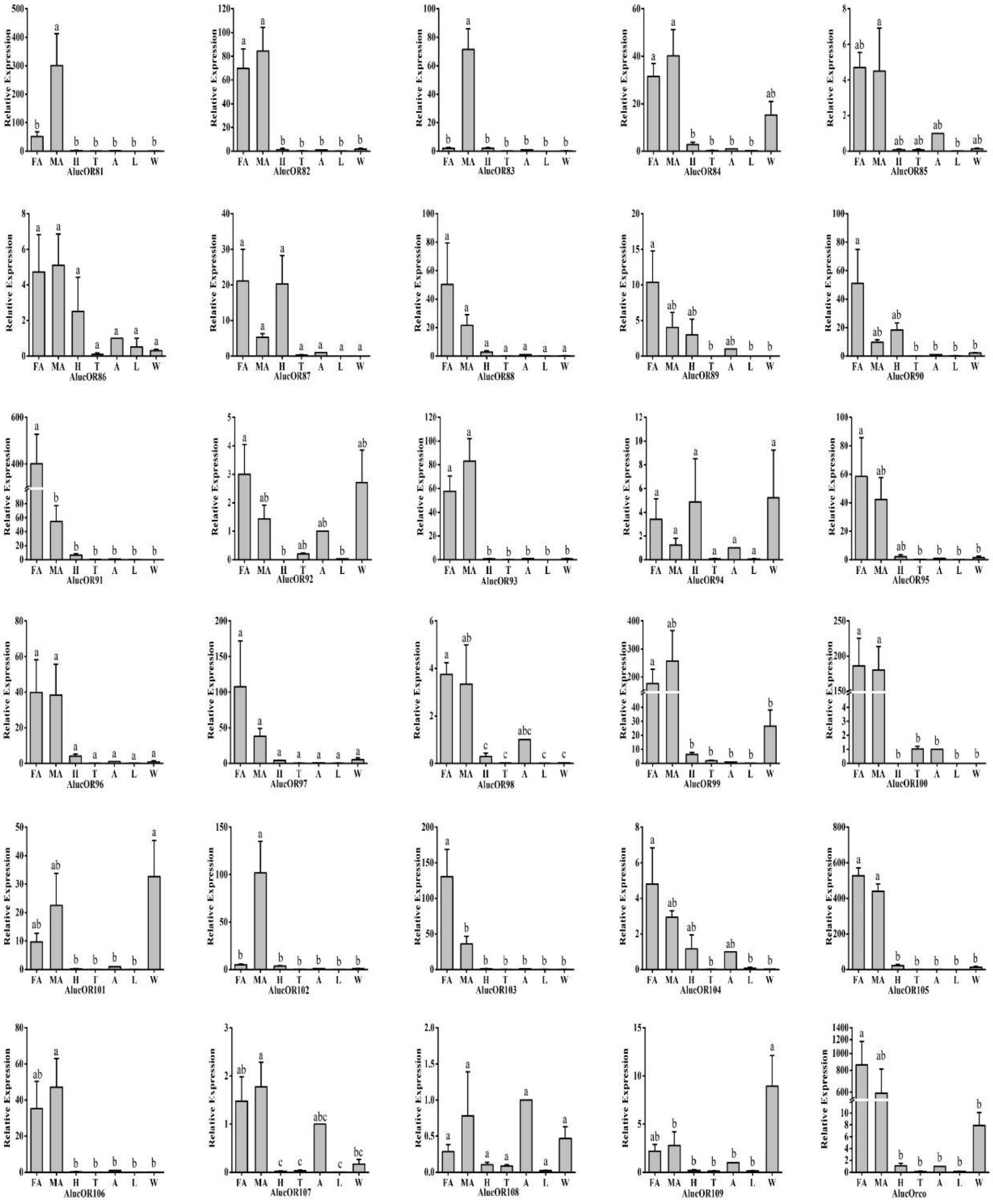
Relative Expression
FA MA H T A L W
AlucOR81
AlucOR82
AlucOR83
AlucOR84
AlucOR85
AlucOR86
AlucOR87
AlucOR88
AlucOR89
AlucOR90
AlucOR91
AlucOR92
AlucOR93
AlucOR94
AlucOR95
AlucOR96
AlucOR97
AlucOR98
AlucOR99
AlucOR100
AlucOR101
AlucOR102
AlucOR103
AlucOR104
AlucOR105
AlucOR106
AlucOR107
AlucOR108
AlucOR109
AlucOrco

Supplementary Table 1.

Sequence	AucOR1	AucOR2	AucOR3	AucOR4	AucOR5	AucOR6	AucOR7	AucOR8	AucOR9	AucOR10	AucOR11	AucOR12	AucOR13	AucOR14	AucOR15	AucOR16	AucOR17	AucOR18	[illegible]
AucOR1	ID	4.40%	4.20%	5.60%	5.80%	2.80%	6.50%	1.70%	5.20%	6.00%	4.70%	2.10%	7.30%	6.90%	2.40%	5.80%	4.90%	4.00%	[illegible]
AucOR2	4.40%	ID	6.50%	5.00%	5.50%	1.20%	6.90%	1.90%	4.90%	4.90%	5.20%	1.90%	6.80%	5.70%	1.70%	5.90%	6.30%	6.90%	[illegible]
AucOR3	4.20%	6.50%	ID	6.30%	4.80%	1.60%	7.50%	0.90%	6.80%	5.80%	3.20%	2.80%	9.50%	4.20%	0.40%	4.90%	6.30%	5.60%	[illegible]
AucOR4	5.60%	5.00%	6.30%	ID	5.20%	1.30%	4.50%	1.10%	7.20%	4.50%	5.90%	2.50%	8.60%	5.20%	1.10%	6.10%	6.80%	7.20%	[illegible]
AucOR5	5.80%	5.50%	4.80%	5.20%	ID	1.60%	5.50%	1.80%	5.10%	5.30%	3.70%	2.70%	5.90%	6.00%	1.60%	4.10%	5.50%	6.90%	[illegible]
AucOR6	2.80%	1.20%	1.60%	1.30%	1.60%	ID	1.00%	4.40%	2.00%	2.10%	3.00%	5.50%	2.00%	2.90%	10.60%	2.30%	2.10%	1.60%	[illegible]
AucOR7	6.50%	6.90%	7.50%	4.50%	5.50%	1.00%	ID	2.70%	6.80%	7.10%	4.70%	4.30%	5.00%	4.50%	1.60%	5.60%	6.10%	5.70%	[illegible]
AucOR8	1.70%	1.90%	0.90%	1.10%	1.80%	4.40%	2.70%	ID	1.70%	2.10%	2.20%	4.80%	1.80%	1.60%	11.20%	0.70%	1.60%	1.90%	[illegible]
AucOR9	5.20%	4.90%	6.80%	7.20%	5.10%	2.00%	6.80%	1.70%	ID	7.30%	8.00%	2.00%	4.30%	7.00%	1.50%	7.00%	5.40%	4.50%	[illegible]
AucOR10	6.00%	4.90%	5.80%	4.50%	5.30%	2.10%	7.10%	2.10%	7.30%	ID	7.00%	2.10%	5.20%	8.70%	1.00%	4.80%	4.90%	3.80%	[illegible]
AucOR11	4.70%	5.20%	3.20%	5.90%	3.70%	3.00%	4.70%	2.20%	8.00%	7.00%	ID	1.50%	7.70%	6.20%	2.00%	6.20%	6.10%	8.30%	[illegible]
AucOR12	2.10%	1.90%	2.80%	2.50%	2.70%	5.50%	4.30%	4.80%	2.00%	2.10%	1.50%	ID	1.50%	1.80%	5.50%	1.20%	3.50%	2.60%	[illegible]
AucOR13	7.30%	6.80%	9.50%	8.60%	5.90%	2.00%	5.00%	1.80%	4.30%	5.20%	7.70%	1.50%	ID	5.40%	0.40%	6.60%	6.80%	4.50%	[illegible]
AucOR14	6.90%	5.70%	4.20%	5.20%	6.00%	2.90%	4.50%	1.60%	7.00%	8.70%	6.20%	1.80%	5.40%	ID	1.30%	7.60%	5.80%	5.90%	[illegible]
AucOR15	2.40%	1.70%	0.40%	1.10%	1.60%	10.60%	1.60%	11.20%	1.50%	1.00%	2.00%	5.50%	0.40%	1.30%	ID	2.00%	1.10%	1.60%	[illegible]
AucOR16	5.80%	5.90%	4.90%	6.10%	4.10%	2.30%	5.60%	0.70%	7.00%	4.80%	6.20%	1.20%	6.60%	7.60%	2.00%	ID	7.00%	8.10%	[illegible]
AucOR17	4.90%	6.30%	6.30%	6.80%	5.50%	2.10%	6.10%	1.60%	5.40%	4.90%	6.10%	3.50%	6.80%	5.80%	1.10%	7.00%	ID	8.00%	[illegible]
AucOR18	4.00%	6.90%	5.60%	7.20%	6.90%	1.60%	5.70%	1.90%	4.50%	3.80%	8.30%	2.60%	4.50%	5.90%	1.60%	8.10%	8.00%	ID	[illegible]
AucOR19	1.70%	2.20%	0.90%	1.10%	1.10%	6.10%	1.30%	6.00%	0.70%	1.50%	2.00%	2.70%	1.30%	0.80%	4.60%	2.00%	2.10%	1.60%	[illegible]
AucOR20	4.90%	8.10%	5.80%	7.00%	6.20%	0.90%	8.40%	1.70%	4.40%	5.70%	8.20%	2.20%	5.20%	7.70%	2.20%	6.90%	7.00%	5.90%	[illegible]
[illegible]	[illegible]	[illegible]	[illegible]	[illegible]	[illegible]	[illegible]	[illegible]	[illegible]	[illegible]	[illegible]	[illegible]	[illegible]	[illegible]	[illegible]	[illegible]	[illegible]	[illegible]	[illegible]	[illegible]

Supplementary Table 2.

Gene name	RPKM (Female antennae)	RPKM (Male antennae)
AlucOrco	170.30	177.23
AlucOR104	59.45	53.26
AlucOR18	47.93	37.01
AlucOR41	47.36	35.01
AlucOR91	45.25	32.38
AlucOR109	33.48	44.74
AlucOR84	31.09	26.81
AlucOR11	30.69	28.33
AlucOR37	30.23	41.49
AlucOR101	28.05	38.96
AlucOR52	27.54	16.55
AlucOR13	26.98	21.35
AlucOR17	24.69	21.34
AlucOR28	24.45	26.19
AlucOR2	24.43	25.86
AlucOR12	24.37	17.20
AlucOR43	22.33	25.31
AlucOR96	21.59	29.95
AlucOR62	21.52	14.21
AlucOR3	21.10	47.08
AlucOR63	20.17	18.86
AlucOR93	19.30	20.24
AlucOR97	19.25	14.69
AlucOR54	18.10	14.07
AlucOR15	17.25	13.48
AlucOR25	17.09	14.36
AlucOR85	16.82	17.93
AlucOR10	16.35	16.35
AlucOR81	16.08	32.38
AlucOR103	16.08	13.71
AlucOR74	16.04	12.82
AlucOR71	16.00	12.93
AlucOR50	15.87	11.37
AlucOR34	15.52	16.40
AlucOR33	15.48	12.62
AlucOR24	14.75	21.10
AlucOR72	14.63	11.65
AlucOR88	14.48	11.74
AlucOR82	13.81	6.33
AlucOR106	13.78	15.39
AlucOR75	13.00	11.84
AlucOR7	12.87	11.82

AlucOR39	12.85	10.31
AlucOR14	12.78	13.55
AlucOR68	12.39	11.29
AlucOR107	12.14	13.78
AlucOR56	11.87	17.77
AlucOR89	11.75	12.92
AlucOR40	11.63	23.35
AlucOR61	11.61	11.56
AlucOR9	11.14	12.52
AlucOR4	10.70	26.14
AlucOR90	10.49	10.03
AlucOR16	10.42	8.87
AlucOR86	10.27	10.75
AlucOR73	10.07	9.96
AlucOR48	9.99	4.95
AlucOR98	9.77	6.48
AlucOR105	9.45	10.98
AlucOR100	9.27	7.37
AlucOR23	9.26	5.53
AlucOR51	9.00	5.89
AlucOR49	8.99	8.13
AlucOR67	8.82	8.38
AlucOR79	7.51	8.51
AlucOR80	7.48	7.17
AlucOR26	7.24	5.99
AlucOR59	7.14	16.58
AlucOR66	7.13	5.22
AlucOR32	7.02	6.02
AlucOR30	7.00	5.49
AlucOR99	6.93	6.19
AlucOR65	5.97	4.33
AlucOR20	5.57	5.81
AlucOR60	5.54	4.00
AlucOR69	5.47	3.67
AlucOR36	5.19	4.63
AlucOR77	5.15	3.09
AlucOR47	5.06	4.43
AlucOR44	4.99	2.92
AlucOR70	4.77	3.97
AlucOR29	4.56	4.07
AlucOR58	4.38	2.62
AlucOR5	4.28	3.88
AlucOR31	3.97	4.38
AlucOR76	3.95	2.18

AlucOR45	3.92	3.04
AlucOR42	3.85	2.94
AlucOR57	3.56	4.13
AlucOR46	3.52	3.42
AlucOR22	3.40	3.39
AlucOR55	3.12	2.98
AlucOR27	2.66	2.65
AlucOR87	2.56	1.39
AlucOR6	2.40	1.24
AlucOR95	2.15	1.33
AlucOR83	2.01	27.50
AlucOR94	1.86	1.25
AlucOR53	1.79	1.23
AlucOR1	1.68	1.24
AlucOR108	1.56	0.99
AlucOR64	1.41	1.10
AlucOR78	0.96	1.03
AlucOR21	0.61	8.04
AlucOR35	0.61	1.33
AlucOR19	0.55	1.72
AlucOR92	0.42	0.72
AlucOR8	0.35	0.73
AlucOR38	0.00	0.00
AlucOR102	0.00	0.53

Supplementary Table 3.

Gene name	Antennal highly expressed OR genes							
	Female antennae	Male antennae	Head	Thorax	Abdomen	Leg	Wing	Fold_change (minimum relative expression level of ORs in antenna/maximum relative expression level in other tissues)
AlucOR10	12.86	44.55	0.53	0.04	1.00	0.00	2.34	5.50
AlucOR7	12.98	31.50	2.21	0.10	1.00	0.23	0.17	5.87
AlucOR30	5.97	9.87	0.05	0.04	1.00	0.00	0.00	5.97
AlucOR79	6.14	15.81	0.08	0.08	1.00	0.00	0.12	6.14
AlucOR2	6.39	7.10	0.18	0.04	1.00	0.00	0.15	6.39
AlucOR99	176.39	257.00	6.34	1.92	1.00	0.01	26.61	6.63
AlucOR34	6.81	8.76	0.18	0.04	1.00	0.01	0.24	6.81
AlucOR22	18.23	15.04	0.74	0.07	1.00	0.02	2.18	6.88
AlucOR66	344.82	905.95	9.82	2.80	1.00	0.93	46.90	7.35
AlucOR97	107.64	38.02	4.08	0.26	1.00	0.19	4.98	7.64
AlucOR72	102.55	36.41	4.60	1.03	1.00	3.94	0.00	7.91
AlucOR88	50.39	21.48	2.71	0.05	1.00	0.02	0.23	7.92
AlucOR23	8.08	10.72	0.27	0.07	1.00	0.02	0.60	8.08
AlucOR53	9.48	8.99	1.08	0.00	1.00	0.15	0.62	8.29

AlucOR91	400.94	54.52	6.46	0.34	1.00	0.08	0.00	8.44
AlucOR3	8.71	30.25	0.38	0.15	1.00	0.01	0.11	8.71
AlucOR4	15.06	105.73	1.66	0.33	1.00	0.00	0.00	9.06
AlucOR49	9.24	11.03	0.90	0.28	1.00	0.02	0.25	9.24
AlucOR96	39.92	38.44	4.08	0.04	1.00	0.02	0.95	9.43
AlucOR75	25.88	16.06	1.49	0.37	1.00	0.03	1.06	10.76
AlucOR40	61.00	162.46	0.66	0.15	1.00	0.20	5.42	11.25
AlucOR12	15.19	34.10	0.35	0.06	1.00	0.03	1.35	11.30
AlucOR24	58.38	64.07	0.32	0.31	1.00	0.02	4.42	13.21
AlucOR55	14.51	16.48	0.11	0.08	1.00	0.01	0.30	14.51
AlucOR74	21.81	25.62	0.99	0.04	1.00	0.02	1.11	19.65
AlucOR105	526.79	439.72	22.37	0.71	1.00	0.05	12.86	19.66
AlucOR95	58.56	42.33	2.14	0.16	1.00	0.08	1.48	19.80
AlucOR81	51.34	300.69	2.25	0.19	1.00	0.01	0.11	22.79
AlucOR15	36.94	26.04	0.13	0.04	1.00	0.02	0.58	26.04
AlucOR106	35.26	47.10	0.22	0.03	1.00	0.02	0.13	35.26
AlucOR103	130.49	35.97	0.98	0.24	1.00	0.17	0.25	35.97
AlucOR63	625.29	528.63	2.07	0.36	1.00	0.50	13.81	38.27
AlucOR82	69.63	84.41	1.34	0.13	1.00	0.04	1.70	41.03
AlucOR11	141.40	95.11	1.73	0.65	1.00	0.06	0.00	55.10
AlucOR62	65.10	195.93	0.15	0.09	1.00	0.18	1.15	56.37
AlucOR93	57.78	83.01	0.73	0.15	1.00	0.01	0.74	57.78
AlucOR61	742.72	1425.64	8.55	0.70	1.00	0.33	11.85	62.70
AlucOrco	859.75	592.47	1.09	0.12	1.00	0.14	7.89	75.05
AlucOR59	284.57	705.30	3.13	0.10	1.00	0.03	1.37	91.03
AlucOR100	185.79	179.94	0.24	1.03	1.00	0.00	0.00	175.40

Gene name	Antennal highly expressed OR genes with a similar expression level between the sexes							
	Female antennae	Male antennae	Head	Thorax	Abdomen	Leg	Wing	Fold_change (relative expression level of ORs in female antenna/in male antenna)
AlucOR2	6.39	7.1	0.18	0.04	1	0	0.15	0.9
AlucOR53	9.48	8.99	1.08	0	1	0.15	0.62	1.05
AlucOR96	39.92	38.44	4.08	0.04	1	0.02	0.95	1.04
AlucOR24	58.38	64.07	0.32	0.31	1	0.02	4.42	0.91
AlucOR100	185.79	179.94	0.24	1.03	1	0	0	1.03

Gene name	Female antennal highly expressed OR genes							
	Female antennae	Male antennae	Head	Thorax	Abdomen	Leg	Wing	Fold_change (relative expression level of ORs in female antenna/maximum relative expression level in other tissues)
AlucOR91	400.94	54.52	6.46	0.34	1	0.08	0	7.35

Gene name	Male antennal highly expressed OR genes							
	Female antennae	Male antennae	Head	Thorax	Abdomen	Leg	Wing	Fold_change (relative expression level of ORs in male antenna/maximum relative expression level in other tissues)
AlucOR21	6.37	243.01	1.03	0.17	1	0.04	6.04	38.14
AlucOR83	1.95	71.47	2.01	0.05	1	0	0.13	35.59
AlucOR102	4.97	101.82	3.63	0.29	1	0.02	0.93	20.47
AlucOR13	3.59	66.64	0.24	0.14	1	0.02	0.96	18.55
AlucOR14	0.91	10.14	0.48	0.05	1	0.01	0.57	10.14
AlucOR4	15.06	105.73	1.66	0.33	1	0	0	7.02
AlucOR81	51.34	300.69	2.25	0.19	1	0.01	0.11	5.86
AlucOR71	4.85	28.39	0.34	0.06	1	0.01	0.34	5.85
AlucOR65	4.84	27.06	0.69	0.09	1	0.27	3.75	5.59

Gene name	Female antennae highly expressed AlucORs compared to that in the male antennae							
	Female antennae	Male antennae	Head	Thorax	Abdomen	Leg	Wing	Fold_change (relative expression level of ORs in female antennae/in male antennae)
AlucOR91	400.94	54.52	6.46	0.34	1	0.08	0	7.35
AlucOR44	24.99	3.55	13.58	0.03	1	0.03	1.59	7.05
AlucOR90	51.04	9.62	18.34	0.11	1	0.23	2.18	5.31
AlucOR58	24.53	5.83	12.04	0.15	1	0.02	22.09	4.21
AlucOR87	21.08	5.28	20.31	0.31	1	0	0	3.99
AlucOR103	130.49	35.97	0.98	0.24	1	0.17	0.25	3.63
AlucOR41	11.17	3.34	13.84	0.55	1	0.17	18.03	3.34

Gene name	Male antennae highly expressed AlucORs compared to that in the female antennae							
	Female antennae	Male antennae	Head	Thorax	Abdomen	Leg	Wing	Fold_change (relative expression level of ORs in male antennae/in female antennae)
AlucOR21	6.37	243.01	1.03	0.17	1	0.04	6.04	38.14
AlucOR83	1.95	71.47	2.01	0.05	1	0	0.13	36.6
AlucOR102	4.97	101.82	3.63	0.29	1	0.02	0.93	20.47
AlucOR13	3.59	66.64	0.24	0.14	1	0.02	0.96	18.55
AlucOR80	3.41	58.47	16.4	0.02	1	0.01	1.27	17.13
AlucOR68	0.12	1.66	4.88	0.16	1	0	0.02	13.94
AlucOR14	0.91	10.14	0.48	0.05	1	0.01	0.57	11.08
AlucOR4	15.06	105.73	1.66	0.33	1	0	0	7.02
AlucOR27	0.01	0.07	0.06	0.08	1	0.02	0.3	6.24
AlucOR76	0.31	1.9	0.77	0.09	1	0	0	6.18
AlucOR38	2.54	15.48	0.24	0.03	1	0.01	22.88	6.09
AlucOR81	51.34	300.69	2.25	0.19	1	0.01	0.11	5.86
AlucOR71	4.85	28.39	0.34	0.06	1	0.01	0.34	5.85
AlucOR65	4.84	27.06	0.69	0.09	1	0.27	3.75	5.59
AlucOR73	2.64	12.77	0.15	0.13	1	0.01	3.78	4.83
AlucOR78	1.57	7.14	0.2	0.03	1	0.01	0.82	4.55
AlucOR64	4.44	19.43	0.23	0.18	1	0	2.34	4.38

AlucOR69	9.57	40.53	0.56	0.03	1	0.02	10.93	4.23
AlucOR18	2.04	7.77	0.13	0.01	1	0	0.21	3.81
AlucOR3	8.71	30.25	0.38	0.15	1	0.01	0.11	3.47
AlucOR10	12.86	44.55	0.53	0.04	1	0	2.34	3.46
AlucOR51	15.02	50.9	8.78	0.26	1	0.39	12.3	3.39
AlucOR67	13.56	44.31	0.36	0.08	1	0.28	11.17	3.27
AlucOR35	0.57	1.82	0.18	0.06	1	0.02	2.39	3.17
AlucOR62	65.1	195.93	0.15	0.09	1	0.18	1.15	3.01

Gene name	Head highly expressed OR genes							
	Female antennae	Male antennae	Head	Thorax	Abdomen	Leg	Wing	Fold_change (relative expression level of ORs in head/maximum relative expression level in other tissues)
AlucOR68	0.12	1.66	4.88	0.16	1	0	0.02	2.93
AlucOR8	13.34	9.2	28.83	0.91	1	0	2.42	2.16

Gene name	Abdomen highly expressed OR genes							
	Female antennae	Male antennae	Head	Thorax	Abdomen	Leg	Wing	Fold_change (relative expression level of ORs in abdomen/maximum relative expression level in other tissues)
AlucOR27	0.01	0.07	0.06	0.08	1	0.02	0.3	3.32
AlucOR33	0.47	0.49	0	0.01	1	0	0.04	2.03

Gene name	Wing highly expressed OR genes							
	Female antennae	Male antennae	Head	Thorax	Abdomen	Leg	Wing	Fold_change (relative expression level of ORs in wing/maximum relative expression level in other tissues)
AlucOR20	0.26	0.54	0.15	0.04	1	0	6.66	6.66
AlucOR36	3.05	7.76	0.47	1.53	1	4.83	27.37	3.53
AlucOR19	0.78	0.59	0.49	0.15	1	0.03	3.41	3.41
AlucOR109	2.19	2.79	0.18	0.12	1	0.13	8.95	3.21

Supplementary Table 4.

Primer name	Sequence (5'-3')
AlucOrcoF	TCTTCAGTACTCATTCGTCG
AlucOrcoR	AGGTAAGTTGGGATTGGAT
AlucOR1-F	TTCTGATGAAAGATGTGAAG
AlucOR1-R	ATAGACGCTGTTTAATGCT
AlucOR2-F	ATCAGTGTCCTTCAATTCCT
AlucOR2-R	TACCCCTAGACTCATATACCTAC
AlucOR3-F1	TTTGAGTGGAACAGCACACC
AlucOR3-R1	CCATCTATCATTGAAGACAACATC
AlucOR3-F2	AGAAGTATGGCCTGGTAAGC
AlucOR3-R2	TCTCAGAGGGTTGAAGTAGC

AlucOR4-F	GAAAATTAGAAACGAAGAAACCA
AlucOR4-R	AGCACCACCGAAAATGAAG
AlucOR5-F	GGTCTCGCAGAATTGTAGG
AlucOR5-R	GTTCAGCCAGTAGGCAGCA
AlucOR6-F	ATCCCCGATGGACAAAACAAGA
AlucOR6-R	TCGATGCTGAAGAAACAAAACG
AlucOR7-F	TTGGCTACCAGAATGAGAG
AlucOR7-R	CCACGTACAAACTGAACAGA
AlucOR8-F	GTGTGCGTGTATTGCTTCG
AlucOR8-R	CCGTGCATCATATTTCTTCTTA
AlucOR9-F	GCCAACTACTTTACCAACTGC
AlucOR9-R	TTTGAAACAGCCTAACGCC
AlucOR10-F	TAAAGTTTTGGGCGAGGTT
AlucOR10-R	TTCTGCAAAGTACGGCTGA
AlucOR11-F	CAGGTACTGAAAGTCCTGTGA
AlucOR11-R	CTTGGTATTCTTGTGGTTGG
AlucOR12F	ACTTTCCCTATCGCATTCC
AlucOR12R	CGTGATTTCTAACCGCTGA
AlucOR13-F	CACTTGACATTATTTTGTGGTAT
AlucOR13-R	ATGGAGAATGACGATTTGGA
AlucOR14-F1	AACCGTTATCCTGTGTAGTGG
AlucOR14-R1	CTGAGGCTGTTGATGTCGC
AlucOR14-F2	ATTTCGCATAAAAAGGCAG
AlucOR14-R2	TATATTGGGGGCTTATTCTAAC
AlucOR15-F	CCAGTTTTTGTAAGAGAGTGT
AlucOR15-R	CCTATGTTGGTGTGGTGAAG
AlucOR16-F	ATCAGTTTCAACAGTACAGTAATC
AlucOR16-R	TCCAACCTCATTCAGACATA
AlucOR17-F	GTCTTGTTTATTCGGGGAC
AlucOR17-R	CATCTGTTCGATCTTACTTAGAG
AlucOR18F	TGCTTGGTGTTTCTTCATT
AlucOR18R	TTCCTGTGGGGTTAGTGTT
AlucOR19-F	CTCACCATTCAAAGAAAGG
AlucOR19-R	GTAACGCAGAGGACCACTA
AlucOR20-F	ACTAAGCAGTACTACGCAGC
AlucOR20-R	GTCTTCTCAATACATCGTTTCT
AlucOR21-F	TCGTTGATGTTCTCCATGC
AlucOR21-R	TAAGAGGACTTTCTCGCTCC
AlucOR22-F	TCTCTTCTCTGTCGGTCAAC
AlucOR22-R	CAAAAAGGAAAATAAATAATGC
AlucOR23-F	GCAGAAACGAGACAACGAC
AlucOR23-R	TGAGAGAGAGCTCCCCTATAA
AlucOR24-F	GTGGGAAGGTAGGTAAGAGG
AlucOR24-R	CACCAAAGTGTGGAGATGG

AlucOR25-F	CTTTTGATTTGGAGTAGCC
AlucOR25-R	TTCATTTCGTTCATAGTAAGAC
AlucOR26-F	AATAAGTAAGTCCATTTCGTATGT
AlucOR26-R	GAGTCGCTTTTGCTCCAG
AlucOR27-F	AACAGGTAGAACACACAGGA
AlucOR27-R	ACTAGAAAGCAGGAATAGGG
AlucOR28F	TTGGTGTTCGTAAAAAAAGG
AlucOR28R	TCCACAAGTGTGTACAGAGTGT
AlucOR29-F	TCGCAGCGCCCACAGTAA
AlucOR29-R	CCGAAGCGTTCAAATCCAT
AlucOR30F	AGAAGATAGGAACCCGAAA
AlucOR30R	TGATGCTACACTGAAAATACG
AlucOR31-F1	GTGAGGTGGCATACAGGAC
AlucOR31-R1	TTGGCTATATAAATGAATAAATTG
AlucOR31-F2	GATAGGAATGAGAAAAAGGTAGAAA
AlucOR31-R2	AGAAAAACTCGGACGAAGATG
AlucOR32-F	AAGCCAAATCCACTACCAC
AlucOR32-R	AACAAACTGCAGCCTATCA
AlucOR33-F	GTTTTAATGATTCGTGTTATGTG
AlucOR33-R	TTATGAGAGGGATTTGGTTTT
AlucOR34-F	GTCTTTTTGGTGATCAGTGG
AlucOR34-R	AGGGTGTTTTATTTGGTTTTA
AlucOR35-F1	AAAAGTCTTCCTTTCGTTTAT
AlucOR35-R1	GGGTAGGTTGTGTTGTGGT
AlucOR35-F2	CGCAAGCAATGATGGTAAAT
AlucOR35-R2	AACAGCCGTCCTAGAGGAAA
AlucOR35-F3	GTGGATGGGTTTACTCTTC
AlucOR35-R3	GGATATCTTTTCCTCTAGGAC
AlucOR36-F	ATTGGCATTTAGCTTTGGG
AlucOR36-R	TCTTCATTACTACGGATTCTGG
AlucOR37-F	ATTTAGGTATGTTTAGTTATGTGTG
AlucOR37-R	AAAGGTCGGTAAGAGTGAGT
AlucOR38-F	ACAGATGACCCTTTGCATA
AlucOR38-R	AAAACATTTAGTCCGCTTG
AlucOR39-F	TTACTAATAAAATCGAGCAGTT
AlucOR39-R	CACCAATAACCATTTGACC
AlucOR40-F1	AGTAAAGCATAAAATCAAGCG
AlucOR40-R1	GGTGAAGGGATACCAATCA
AlucOR40-F2	ATGGTCAACGCTGATCCAG
AlucOR40-R2	TCCTTACCCCACAGACACAC
AlucOR41-F1	TTATTTTTCCTCCTCTCCTC
AlucOR41-R1	CCGACTCTCTTCCTCTCAC
AlucOR41-F2	CGAAAACCAGCATTGAAAAC
AlucOR41-R2	CAAGATCCAGGAGCCGACA

AlucOR42-F	AAAGAGAAGGTTGTTGACTTG
AlucOR42-R	AATATAATAGCATTGATTTGAGTG
AlucOR43-F	AGACCCTGCAAGAGTCTGG
AlucOR43-R	TGCCCTTTGTGTTTATGTGA
AlucOR44-F	CTATTTTTTTTATTTCCCTGC
AlucOR44-R	TCACCCACATATTGTGTCC
AlucOR45-F	GATGTTAGGTATCACAATGTCA
AlucOR45-R	GTGTAAGCTCGTAGCTGCT
AlucOR46-F	CGCTTTCATCTGTTTTTTCAC
AlucOR46-R	CAATGCCCCTGGTTTCCA
AlucOR47-F	TCTCAAATACTCGGGTCAC
AlucOR47-R	CCTAACTAATACAGAGTCAGTCATA
AlucOR48-F	GTATGTTTACGGTTTCTTCG
AlucOR48-R	GTACTATCCAGCTTCCTCG
AlucOR49-F1	GTACAACCTTACTCGAACGAA
AlucOR49-R1	GTCAGGACTCCCATGAACTT
AlucOR49-F2	AATCAGCCTGCTGCATAAA
AlucOR49-R2	GACCTCACGTCTGCAAATG
AlucOR50-F	TCTGACCAGAGAAGGAAGAG
AlucOR50-R	ATGGAATACCCAGTAATACAA
AlucOR51-F	CCGATACATGGTATGGAGC
AlucOR51-R	TGAAAATTATATTCTGAGAGAGTGA
AlucOR52-F	GAGAATCGGTTCTGTGGAA
AlucOR52-R	ACGCAACAAGGATTTAGGA
AlucOR53-F	GTTGTTTTTTTGGAATGACT
AlucOR53-R	TTCAGAGAGAAGTTCAAGTTT
AlucOR54-F	CATATCCACAGCTACCCAA
AlucOR54-R	TTCCTGACCCAAATACAAAC
AlucOR55-F	GAGACCAAACATTTTGCCA
AlucOR55-R	AAACCCCGTACACCTTCAA
AlucOR56-F	TAGTAAGTGGGAAAAGGAGC
AlucOR56-R	TATTATACGTGCAAGGTAGAGT
AlucOR57-F	CGACGGAGAAAATAGAAAAA
AlucOR57-R	GAGACTGTGGTAAAGAAATAAGG
AlucOR58-F	ATGAAGGTAGGTGGTGGTC
AlucOR58-R	AGTTCTTGTTGTATTTTGAGTGA
AlucOR59-F	GCTCGTCATAAGATAAATCG
AlucOR59-R	TACAAACTGGTTACTCCTCG
AlucOR60-F	AGCGTTCCACTCATTTTCA
AlucOR60-R	CTGTGCTTCCACTAAGTTTCT
AlucOR61-F	TTCGCAAAGTGGTCTAAGG
AlucOR61-R	TTCAAGGGATTGAAATCGT
AlucOR61-F	ACCGCTCTTCTGTATGTTGG
AlucOR61-R	TCTGTGGTACGCAGGCTTC

AlucOR61-F	GGTGGATTCATGGGCAAA
AlucOR61-R	CCAACATACAGAAGAGCGGT
AlucOR62-F	ACGACTCAGTTCGTTCTGG
AlucOR62-R	GTATTTCGAGGGAGGCTCA
AlucOR63-F	GGTGTCTATCTGCTACCACAC
AlucOR63-R	AGTTCATACCATTTCACTCAAG
AlucOR64-F	CGACATTTGAAGTCCAATT
AlucOR64-R	CCCAATAATAAAGCACAAC
AlucOR65-F	GCCAAGGAGTGAAGAAATC
AlucOR65-R	ATAATCATAAAACAGTGAAAGAAG
AlucOR66-F	GGATGGAAGGTTATGTTGTT
AlucOR66-R	GGCTATGATGATTGCTGATT
AlucOR67-F	GTAATTTATCACCCGTCCG
AlucOR67-R	TGCCAGTGTCTCAATCTTTG
AlucOR68-F1	TATTGCTCGATTTTTTTTCTG
AlucOR68-R1	TTTCTTGTACTGGCGTTTCT
AlucOR68-F2	GACAAAAACGAGCCCTGG
AlucOR68-R2	TCATTATGCGACTTGACAACC
AlucOR69-F	GAGGTTGAAGAACGAGTAGGA
AlucOR69-R	GGGAAAAAGATGAACAAGAAG
AlucOR70-F	ATTCTTGGCGCCTCAGGTT
AlucOR70-R	AGTAGGCAAGGGCAACAGC
AlucOR71-F	GTAGTGGATTGAAATGGGG
AlucOR71-R	TTTGTGCTATTCTAAGAGCTAA
AlucOR72-F1	TCTCTCTTTTGTAGTGCCC
AlucOR72-R1	CTTATAACGCTTAACAGTATCC
AlucOR72-F2	ATACCGTTATTCGTGATTGG
AlucOR72-R2	TTGGTTACATGACAGGAGCT
AlucOR72-F3	TATAGATGGGACACCAACAT
AlucOR72-R3	GAGGGCACTACAAAAGAGAG
AlucOR73-F	TAAAGGAGGGGCAGGTAAC
AlucOR73-R	ATCAAGTGCAGTGCAGAGTG
AlucOR74-F	TGTAAGAAGTTCGACGTGG
AlucOR74-R	TGTATCATTGAGGCGATAAG
AlucOR75-F	GGGGGAATGTGGGTACAAA
AlucOR75-R	ATCAGGGTTCAGGCTTGGA
AlucOR76-F	TCCGTCACGTCTTGACCG
AlucOR76-R	GCGATCATAGGAGTCAATACG
AlucOR77-F	TGTTGAAGAATGAATAAGCCG
AlucOR77-R	AAGGGAGCAATAAATGGGG
AlucOR78-F	GAACACCTACCAACAAACAGA
AlucOR78-R	ACCTTCAAAAAGGACCTACC
AlucOR79-F	CAGACCAACAAACGGACAG
AlucOR79-R	GCGAAGATAACTTAGAAAACCC

AlucOR80-F	CGTCTATGTTGGTTATTTTGT
AlucOR80-R	TTTATCGGTTTTTGGCAGT
AlucOR81-F	ATTACAAGCACATTGTTCG
AlucOR81-R	TAAATATCTTCTACTCCCGC
AlucOR82-F	GGACTAGAGGTTCGGTGATG
AlucOR82-R	GTGCTATTGGACTGGGGTTA
AlucOR83-F	GACACTTTCATTCGTTCCTA
AlucOR83-R	GTGGATTACTTCGATGGATA
AlucOR84-F	TTACGGCTGGTTTTGGAG
AlucOR84-R	GAATTGAGTGGCGATTTCT
AlucOR85-F	AAAACTGGCTTATCGGAGA
AlucOR85-R	AATGTAAGTTGTGGTTAAATGTG
AlucOR86-F	GAATCAAGACCCTCTCACAA
AlucOR86-R	CTAATAGCACTGGACCACG
AlucOR87-F	TTTTGAAGCTGGTTAGTAGGC
AlucOR87-R	TGAACTGGTTTGGATGGTG
AlucOR88-F	TAATAACAGGGTGGGTTACG
AlucOR88-R	GAAGATGATGCACTTTGGG
AlucOR89-F	TTTATTTCTACAACATTTTTACTAC
AlucOR89-R	ATTTGTCAGGAACATTCTCA
AlucOR90-F	CATAGCAGAAAATAACAGCAT
AlucOR90-R	CTACATCGAAAAATCGCA
AlucOR91-F	ATGTTGAGGAGGGTTTTCC
AlucOR91-R	GAGTTATTGCTGCGGGTT
AlucOR92-F	TTGGGTATAACCGTTTTCG
AlucOR92-R	TCAGTCTCTGGTGCTCTTTG
AlucOR93-F	TAAGTGGGAAGAGCCTTTA
AlucOR93-R	ATGTGTCATAGTTGAATCGTG
AlucOR94-F	TGTGTCAGTCAGGCGGAT
AlucOR94-R	CAAGCACTTTGGCTCGTC
AlucOR95-F	TGCTTCTTTATGCCCGTA
AlucOR95-R	GGTTGGCCTCGCTTATTT
AlucOR96-F	TTCGTCAAGTACTACGGGA
AlucOR96-R	GAGGGAGTGAAACTGTGAAC
AlucOR97-F	TGCCAAAACATACGAATCA
AlucOR97-R	TGAACAAGAAGATGAGGAACT
AlucOR98-F	TATGCTGCTGCGTCATCC
AlucOR98-R	AAGATTGGTCTCGGAAAT
AlucOR99-F	ATGAAACCACTCGTCCAAT
AlucOR99-R	AGTAATAAGTAGGTATCTAGGCAG
AlucOR100-F	GATAGGTGGCGAACAAAC
AlucOR100-R	CGATACCTTTTGCTAAGTTG
AlucOR101-F1	AGATGTGGGACATGAGGC
AlucOR101-R1	AAGGTGGATAGTAAAGAGGGTA

AlucOR102-F	ATTGTATCCCTGCAATCA
AlucOR102-R	GAATCCCGAAGTTCCTCA
AlucOR103-F	GTGGTTGGGATTTATTATTG
AlucOR103-R	ATTTTGAAGGCAGTCTCG
AlucOR104-F	CTTCTCCCTACAACACCTCG
AlucOR104-R	GCAAATAATCATTCTGATAAACC
AlucOR105-F	TTCTTCCTTCAACCACGGT
AlucOR105-R	AGGTCAGGGCTCAGCGATC
AlucOR106-F	CTGCACATCCACTACCTACG
AlucOR106-R	AATCAGCAACCCTTCACTAAC
AlucOR107-F	GTTGCCTCAAGACCACGA
AlucOR107-R	ACCCCTCAATTGCATAAATT
AlucOR108-F	TAATTTTCATCGCTTCGGC
AlucOR108-R	TGCAGTGAGGTTGGAGTGC
AlucOR109-F1	TGATGCTTCGTTTACTTCGC
AlucOR109-R1	CACCAGTTTAATCGTTCGG
AlucOR109-F2	AGGTAGTTGATGGACAAGAGTT
AlucOR109-R2	GCATAATTGAGCAAGCAGAA
AlucOR109-F3	TAGGTTTGGACTGCATAATTG
AlucOR109-R3	AGGTAGTTGATGGACAAGAGTT

Supplementary Table 5.

>AfasOrco
MQKVKMHGLVGDLWPNIRLMQLTGHWLLEYHEETGGMARLIRIAYCWMTTFVVYLQYAFL
VCFLILETYNSDEMAAVTITTLFFLHSVTKFTYFAIRSKYFYRTLSAWNQVNSHPLFAES
NARHRAAALSRMRKLLMIIGVVTILAVFGWTTVTFLDDPVWDKTDPDNVNETISVEIPQL
MVYAWYPWDAKTGMTYFMTFAPQLYWLFITLAHSNLLDVLFCCFVIFSCEQLKHLKEILQ
PLMELSAALDSVVPNSGDLFKSGSAGSNVALISNGDGGNDFDVRGIYSSQRDFSGFQGGM
TNGTTVGPNGLTKRQELLVRSAIKYWVERHRHVVKFVTSIGDTYGTALLLHMLTSTVTLT
LLAYQATKIEGVDVYASTTIGYLVYTLGQVFVFCIHGNELIEESSSVMEAAYSCHWYDGS
EEAKTFVQIVCQQCQKSLTVSGAKFFTVSLDLFASVSLNKTHITILLY
>AlinOrco
MQKVKMHGLVGDLWPNIRLMQLTGHWLLEYHEETGGMARLIRIAYCWMTTFVVYLQYAFL
VCFLILETYNSDEMAAVTITTLFFLHSVTKFTYFAIRSKYFYRTLSAWNQVNSHPLFAES
NARHRAAALSRMRKLLMIIGVVTILAVFGWTTVTFLDDPVWDKTDPDNVNETISVEIPQL
MVYAWYPWDAKTGMTYFMTFALQLYWLFITLAHSNLLDVLFCCFVIFSCEQLKHLKEILQ
PLMELSAALDSVVPNSGDLFKSGSAGSNIALISNGDGGNDFDVRGIYSSQRDFSGFQGGM
TNGTTVGPNGLTKRQELLVRSAIKYWVERHRHVVKFVTSIGDTYGTALLLHMLTSTVTLT
LLAYQATKIEGVDVYASTTIGYLVYTLGQVFVFCIHGNELIEESSSVMEAAYSCHWYDGS
EEAKTFVQIVCQQCQKSLTVSGAKFFTVSLDLFASVSLNKIHIIILLY
>AsutOrco
MQKVKMHGLVGDLWPNIRLMQLTGHWLLEYHEETGGMARLIRIAYCWMTTFIVYLQYAFL
VCFLILETYNSDEMAAVTITTLFFLHSVTKFTYFAIRSKYFYRTLSAWNQVNSHPLFAES

NARHRAAALSRMRKLLMIIGVVTILAVFGWTTVTFLDDPVWDKTDPDNVNETISVEIPQL
MVYAWYPWDAKTGMTYFMTFALQLYWLFITLAHSNLLDVLFCCFVIFSCEQLKHLKEILQ
PLMELSAALDSVVPNSGDLFKSGSAGSNIALISNGDGGNDFDVRGIYSSQRDFSGFQGGM
TNGTTVGPNGLTKRQELLVRSAIKYWVERHRHVVKFVTSIGDTYGTALLLHMLTSTVTLT
LLAYQATKIEGVDVYASTTIGYLVYTLGQVFVFCIHGNELIEESSSVMEAAYSCHWYDGS
EEAKTFVQIVCQQCQKSLTVSGAKFFTVSLDLFASVSLNKTHITILLY
>AlucOrco
MQKVKMHGLVGDLWPNIRLMQLTGHWLLEYHEENGGMLRLLRMAYCWMTTFSIYIQYAFL
VCFLILETYNADEMAAVTITTLFFLHSVTKFTYFAFRSSYFYRTLGAWNQVNSHPLFAES
NARHRATALSRMRKLLMIIGTVTILAVFGWTTVTFLDEPVWDKTDPDNVNETISVEIPQL
MVYAWYPWDARYGMTYFMTFVFQLYWLFITLAHSNLLDVLFCCFVIFACEQLKHLKEILQ
PLMELSAALDSVVPNSGDLFKAGSAGSDIALIGNGENGNDFDVRGIYSSQRDFSGFQGGV
VNGGTVGPNGLTKRQELLVRSAIKYWVERHKHVVKFVSSIGDTYGSALLLHMLTSTVTLT
LLAYQATKIEAVDVYAASTIGYLVYTLGQVFVFCIHGNELIEESSSVMEAAYSCHWYDGS
EEAKTFVQIVCQQCQKSLTVSGAKFFTVSLDLFASVFGAVVTYFMVLVQLK
>AlucOR12
MKFIDKLAEEEDDELIEILKGNYWHFLFYSMTFIRWKRPRIAIALISAYAIWIIVHLVIG
IYSIYLAADERNWAVVGLVTHHMVLGALAIYLPIFCNTGGFREVMADMHRTFTTDIGQYS
GGNMYAEQACIDIKKDVRRQTFVYYINPALVAAAGSLALAGPFLTKWFSGMENPYSPNGL
SLKLPTALYYPFPTDSGVVFYAIVLTQVISGTILGYLILAPQLVFINLSQNLKRELRFVG
YSMETLVRRAMRMTFENNVWKRKVTELDVDDTEFQQNVELSIKETIIHHQKASKLLSTAQ
VSVKGPLAASYIFGLVTIAISLYNITLALKTNDIGSLTTFLLLLSSEVIGTFINCGLIGS
ELTEQSEDVTEKLYFIEWYNFSVKNRKMFFTFQTAITQPYEIKAGGVTPMNMETFSDIMN
SAYSFFNILQTIE
>AlucOR18
MSFSFVEKYQLSPETEKTMVTEYSYLLYVGGLLINYRPKVWIISIAQTSIFIGLITSYTI
IFIISTAKSSNFVAFSQNLNYASLCCICLGLYFAGLSHRSAFVRLMEIIHDDFYDYGDSF
DNAEVAMWKSSLRTFKIIIVVGIPTYLIIIAVSIVLGDYIDTALGYDSTDEDYLGEIYQK
APLNLWYPFVVTNMFLRVAVTLSQMTTAAILATTLATGDVMMLFLGQTVALQLRILCLAA
TKMDQRANLMYEKGLARSSSGDKEDLDGCYKLCIKQLVQHHLIIKEFYKTYYTIAKWPTA
IAFMNGSLMIAMSIIVAMNGNEETPSTYISTYLLLVAEVLSMWLLCETGQNVNTWSEKLF
MDTYEFNWNGLSVPNKKMLLIFKENIKKPLLMMAGGLTPINRDTFATIMNTSYSYVNLLR
ASERRSND
>AlucOR28
MAGYGRLEDGDIVDGLSIWYLKASGLWEMFNHHRETGGRSKVLKFWMAGMIIAYSPVFVV
SVVGPFFAEKDLEGMSLVVLNPMSTVQMVVKFGILWFHMEKQSRLLDLMKKNFLACVPPD
KEAEVSRILGDAVKEANIYTFFGTRINIITVLLWSILPVLRSEYFRITLGITIFGTPLRH
NKLLGFSYPFDYDASPGNEIVFVYEFLLVLSAGLIITVMECLVAQLVVLLTAYLKVFQYF
MEELKSTHDPKFDKEQLLLYVKEHQKLMRVGDEVCDLYNFLITVQLSTGLFILIIAIFNF
FLSSGNGDVVVMIKFVVYTLYTLVEICVYCYAGSNLETTSEDVCFAAYSCEWYEMNPDFR
KTLQMMMVRSRSPVVLKAGKLYPLNLITLTNIVQMAYSTSMLMYQQTHN
>AlucOR30
MVEKSNFHVKRAQLFKAYNSIHWLTLTKWFYEDYPVEKLWSDKRLWIHLSIVIICQSSIT
MFKVFHLISEENFFIFLTSLTSFLVIVLVAVRTYILYQFPTFRQLYFKPEVFNCNLHRPT

RSLALLTEAITHSRKVGMWCLVLFITFDVAFLVLPIVPPILEIIDGTNKTYDELIPQYPS
INPVSLSWLSKELKYAFDLVMAVFNTIPWVGFVVVYYTVVQLFRAQHKIMMTAMLPGPPV
PGDGREPLELKLWIQDHALIRKLVYKLRNTISPALAGTICVNVFTVGLNMLALVSSPIGS
DAPMFTRYLYYFSFGTYSALSIFDIFIHCWLASEITNCGEDLSYALLKSDWQNDLKRSHH
HYVLPLMLCKKQIRFTGLGLIPVTLTTFTETIRVSYSYFTLLRKTDD
>HhalOR1like_isoform_X1
MWDWRQLNWLYYFGWWPSAAKTEVGYKINRVYGIALFIWDFIQIGPEVAALYIALSNGSL
KGTVLNLNTVLMGGVCFMKITGILLNEANIKWIIAKLEEMENRGKNMLGLNEYKMIADYR
DRRCKLLVIIVFTYLSGLVQWVVRPIYDICNGRTSLIIEAVIPWDKDTIYGWTIVFILQF
VHLTTAIMALIIIYVLYLSIMEMILCQIDVLHYSLKKLDFSPPGADYHSSITLRYCVKQH
QDILSLCYRFHKVVNVQLFVFMMFSTVVLCLSVFELSSIKDTTLFKVSNLLELTLNTVFL
IFMYCLYCHNTVDKLTEGTLRAAYKNNWYMGREEDKKSLDILCTMSIKPFEFGFIIPVNL
DTFITVLKSAFSYYNFLKAIADEE
>HhalOrco_isoform_X1
MQKIKMHGLVGDLWPNIRLMQLTGHWLLEYHEETGGMVRLIRLAYCWLTTFLVVMQFAFL
ACFLILDTYDADQMAAATITTLFFLHSITKFAYFAIRSKYFYRTFGAWNQVNSHPLFAES
NARHRATALSRMRKLLMIIGVITIMSVMAWTTVTFLGDPHREITDPEDVNSTITVEMPQL
MVDAWYPWDAKTGFCFFATFIYQLYWLFISLSHANLLDILFCSFVIFACEQLKHLKEILQ
PLMELSATLDSVVPNSGELFRGGSGGSNMPLVENDGNDFDIRGIYSNRGDFSGFGQTAVS
TIQTNGNGIGPNGLTKKQELLVRSAIKYWVERHKHVVKFVSSIGDAYGSALLLHMLTSTV
TLTLLAYQATKIEGVDVYASTVIGYLLYTLGQVFVFCIHGNELIEESSSVMEAAYSCHWY
DGSEEAKTFVQIVCQQCQKSLTVSGAKFFTVSLDLFASVLGAVVTYFMVLVQLK
>HhalOR9alike
MWDWRQLRWLSYFGWWPAAAKTKRAYRLLRIYGIVLFLYDFLQLGPELIALYLVICKGSM
NEVVLTLNNNLLGLGSAWNIGCVLYNEKNIMWIISKLEEMELRVKKMIGNEEYDKYAAYK
YKLCRNYVLTIFIFLAGLIQLTIYSLYWTYHGHVSYIVETWVPWNTDNLQSLIILYTLQV
VHSYTGLTAYATVYHLYMSIYEMILLEIKAFHIALSKLDFSPPGVGSHPPVSLDLCVKFH
QDLLLLCRKFNETINVTLFLFIMFSSLTLCLSVFELSLIYDRGKLTMLIELIMLTMSMTY
FYCVCSQDVVEQMTVGTLRAAYDNNWYVGCAKDQKALHLLCTMAKKEFQFGFIIPANLAT
FITVIKSAFSYYNFLTAMDLQE
>HhalOR94alike
MKPRNPEYGAIELCSCIRHLKIKQDMCTLGIVTSVLATGGSITLFYFFYQNRLEKFTDNW
NALNDNILNSDLDKKDFFRQMFLQVAKNNESFTKTILFVVFWTPIIYCTPVPVVDAIKQS
YRTNLPLPILYLYDDRQPVVYEVTFFLHMMGLVISVMKKFGNDCFFLALFKIHIAYLRYL
SVAIKCEGTKFSKCNNKIIKQNLISWIKIHQQIVKNAQDLIRLYTPIIIVYHVNLICIVV
FGLFTQIKNDRDSSVQRIGTAMFCTVNIFQLYMQCSSAEELTNEAEKVSQEIYNTPWNEV
DECNADIIRLVLKMASRPVEVTAFKAPTFLLNKQSFVAV
>HhalOR92a
MVMIPPFHYTRKQMVMLGILELPNAPKFLRNIITVVIIALSFNSNLSSFLYFLLEDKPFF
EKTESILNCIGILHVFSKALVLPFKRNVLLSVMSDLDEMSVDSNRFQEFRKGYNLNMMLS
RKIPQTVLLSMCTLMMCGTIVSIVRYTTQGTVTPPVQLYIPFKDNISLTILYNYFLVLPT
TFVFSVMISFLISLSLNISIQISYLIMKLENLGMDLSSKEIDSCIILHQKIMRVMTKVNS
LMSGLLFFEYLLTSMQCCLSGYQLLADKKNDGIAGFFYHCTFFAISVTFSSVNCYCGDII
KLKSENIFEAAYCNNWYSLSNVERKKLLVLQLASSKPLTLSYRHLITFDLALYGIILKGA

YSLVTVLQTMETV
>HhalOR85d_isoform_X1
MIIMGVIPILVAIVGGVRVIPMLAVFPFDPTEYFFVFCATVLLQITGGYTCTLRALCFEN
MFNMFACRQLALIRQLSRELRRILKIPHVDDSGELKYQNDEGILYSPEEAKNVVIEELKQ
WVKNHQKSMRMAKELQDMYSISLCIQFAFTGLLLCTTAFVMANKVGGMMNLFFCGAYLIG
LFIELLITCRIGDLILYESNMLERTVEGTHVYVLPSDVYKNWLRLILTRAKVPTRLSALG
VFPLDMETYKSFIVLTYSFFTLLKELKHET
>HhalOR85blike_isoform_X1
MLGIKLASYETDPAKLTVFRNCLIYAGALNDGTLKSKFFIFYRAYGLIVGFLQYAIMATK
TTEFMTVVEVFHWMCDFTIMTSMTMSCLYYNPILIRMEARIKAGFFDYGGPLTPEQIKVR
ATMNRNSQLMSKLYCSLCYCGLVATYIHVFFAKKEYLLPYPMWFPHEIRNMYQYVLTLIH
VFIVAETMTICAFSQLSAFVALSSHLIAQYKILIIAIKEIDLIAGTKATPEEKILKMHAK
LKTCVKHHVIINKYFDDLHKLFSIPLLTTAIFICLAICTLGFILLSPNVSVPVIGALIIL
FLPEMFIIVVYCVYGQKVADVCEEFGHTIYYSDWYTKPLSVQRDFLMILIGSRKKRQLTG
FGLHEFSMKGLSEIIKATFSYFNMLRAMN
>HhalOR83alike
MEGEAKPNLVDRDVLYNFEQERELLMMISCYKVSKSSKLNHFLSLSYVSIVWLFVLFELC
MGLYSVVLTIDSPKTQLLETLHTVVLTFYVIAHMTNRLQSGFDAALEIINKGFYTYDENM
GETHYEIRKEYVRRIRLVNKWFRIIIIYSGISFLMFNTAKKYLENVYKTEPSKIPINPYF
PIPYFMPFDTSTVVTFTSAYLLNVALEFFICSVTICIDEIYVSLIEQLKAQFVILNLSIS
NIVERALRRYQDGKAGSVPNVEELYQQKEFQDCVLQCLKENIRHHHALLRFTALIRNYVQ
RTFFLVVITGGLALALAVLVITKSKLTDQELLGVGTFLMMLLTELVFILQFCLYGEEISN
ESGKLFISLWSTPWWNFDKRVKLVLSIMMLNTKKPIIFKTSFLNVSSSLESFSNDN
>HhalOR82alike
MGCLGPIADSDIIDGLSIRFLKFFGLWKVINDYRTTGKKNSIIRFTVIISFILAVPYVLF
QYLSYSSIKVDLQKATFLNLYPLPALQMICRILVFWFRMDRQCRLYNLLKKDFLHIPENK
RVLVDKVYQKICKTSNICCTASMIVNFSIIGLYIFNPGISVDYILYHTGNMDAVTTGRKK
ILGGWYPLPMAQTPYYEIIFVYEATCVSWAGILLAVYFCLFFQVLISLYAQFTVLGVHIS
TLKFQSNKKDRKCDTKMFKELSQILRDHQKLLRYTDELKSVYNPLVTLTLGMGILILIIG
AIQFLLGKSNSPGFIFKLLQVFIFQGVEVSMFCFGSSFIEMASSDLHFTIYSSDWYMAGT
KFRKAAQMMMIRAKKGETLTAIGMYPVNRETLMTILQFTYTTSTVLSRITE
>HhalOR67clike_isoform_X2
MKLTRKLYVLAGILTEHNTPTSWKVKLISNLAVCSVFLFNLYAFCNGCVVSGLTKDCISH
TIVFIVVHIQSYIKFGILVTMRGTRMKYILDFVEGYVFVNDSYAKHIYLTSFYIIALTVL
AYAIHPLISHSLPFYYETPWGSESFSAFASSYFVMFFDLYLIQFVSTINDFTYLMCADAI
CYRLNRVKSLLESIEGEEDEGKLIRAIKEHQDILRTLNMLADTISPNFFMQVFATLSIVI
FSAFTAVTTEALHFAAYNNKWYNCNRRMKMNLQILSSFTREPFEIRGCSIVRMNLQTFKE
VVTDSYSYLMILITMDDTS
>HhalOR67clike_isoform_X1
MKLTRKLYVLAGILTEHNTPTSWKVKLISNLAVCSVFLFNLYAFCNGCVVSGLTKDCISH
TIVFIVVHIQSYIKFGILVTMRGTRMKYILDFVEGYVFVNDSYAKHIYLTSFYIIALTVL
AYAIHPLISHSLPFYYETPWGSESFSAFASSYFVMFFDLYLIQFVSTINDFTYLMCADAI
CYRLNRVKSLLESIEGEEDEGKLIRAIKEHQDILRTLNMLADTISPNFFMQVFATLSIVI
FSAFTAVTSNESQHFSACLSTLATLLTYCWLGQMVTNATEALHFAAYNNKWYNCNRRMKM

NLQILSSFTREPFEIRGCSIVRMNLQTFKEVVTDSYSYLMILITMDDTS
>HhalOR67clike
MAPERVMEKDLMDGLSIFFVKYLGFWGTVNTYRTSGKVNLLFKIQWFLTFLCVPFPIFQF
MSPAYIKFDLEKATIIILNTTSFFQMTFKQAVYSMNIKEHAILLEVMTKDILRSLPEYKK
PHAKRIFEKISKRCNFWCFIAVVITFTAVSLWTMNPCISSEYIANHVGNMKDVTTGPKKI
LGGWYPVPFTRSPWEEIVYAYEFLWFFWIGYNVAIYELVITMEVLTLHAQMSVLNYHVST
LSKKEIVQHSGKKGLTQREVEDLFYQELLAIIRDHEMLLGYGNRLRNCFNAYITMLLATG
GLLLIASIFQFLFGAKDAVVSVNYMLYLLYEVAEFIFLCTATTMLETSSTNIAFSIYSSE
WYTSDKRSRDTIQMIMIRSRKPMSLIAVKMYPVNVETLMSVFQFAYSASALISRMVE
>HhalOR63alike
MAYEYLPSVYIRRKDAEESTTKYSKRRSEVSVLYQVLEPILLLMRLAGRFPYAINQKPDK
EMHCGWAVYSAAVGVLQFAGVYFTSNLSYSLSLGNFDEQIFATVTTIVCFILGLNPFLTW
FEAPWLSAYLDKWSAYQDELRSFDVILDTRLQKWLLLWILALFPYTAVVAYFLDVRRGDP
TAFPVYLLLQIGSYLLLTLWFFMVFFIEDTASRLLARIGDASAEREVAILKKLWLTLANL
TTELGHVLSLTLILFMISCSIIGIANCYSLLFFLRDCFLDNCDSSSYQQIPIISQVGTLI
VSAIIIIAICEHGHRCTVSVGSNFLKEILKINFSLRNENTQRELHSLVQTILLRYPDMAL
GCYFTVNRRLLATMVTTAITYLIVLLQFRSTGIKT
>HhalOR59blike
MSEPIRESDIFDGQTTFYMKYVGIWKIVNTYRTSGKMSLVFRLEWYLTLLLSVPFQVLQV
ISPSYIDVDLEKATILILNTVSFLHMVAKHGTFWWNIKGHAELFRLMTKDLLTSIPQYKA
AEAKKIYQDATKRCNFYCRMIVTITYSVWSLWTFNPTVKSDYIQFHTGNMKDVTTGPKKI
LGGWYPLPFSQSPWTEIIYFYEAILLLWCAVIVSIFDTVVTQEVMGLYAHLSVLNFHIST
LKKEEIIFHSKREVHTEQEAEDLMHKEFVAIIRDHQYLLRCGKIIKECYNTYITALLLAG
GNLMIITVFQFFYGKKDIPTTINFVFYLSYGVMEISLLCWTTTLLETASTNIAFSIYSSD
WYTCHKKLRNTGQMIMLRSEKPLSLAGFKVYHVNLETLMNIMQFTYSSSALMSRMVE
>HhalOR4like_isoform_X3
MLGTITKFYKTDLLCAIENLHFLIFVTVELVAMMSFLQKRAVLVSMYITIGKGFFDYENT
LDDECLELKRDAYDKTDSRKRLVHHSFVTVVMSACITISVFRPAVLILFPKENMGNPNDG
MIRVALAPMWSPFDNTQWHGIVIVWLLEYIVAWTTPGIVFGATFFVLFSLEELGIQLQIL
KKSLTNVIQRAERLEQGMEENIKLCLKYSVRHHQLLFEFHDKLNEVISLPGLGLFVSFSI
MLCMSGFIFTLKEVPLVSKSVFGLFLLSECAMLFALCYFGENIIELSEEIGDALYNSDWA
IYSKVMQNYMLIIQMRSRRTMRLTLMDFMDVSRNTFSNICSTSYSYLNLMNEFN
>HhalOR4like_isoform_X2
MFLGNPLLPVAFILSGFILIYFMLGTITKFYKTDLLCAIENLHFLIFVTVELVAMMSFLQ
KRAVLVSMYITIGKGFFDYENTLDDECLELKRDAYDKTDSRKRLVHHSFVTVVMSACITI
SVFRPAVLILFPKENMGNPNDGMIRVALAPMWSPFDNTQWHGIVIVWLLEYIVAWTTPGI
VFGATFFVLFSLEELGIQLQILKKSLTNVIQRAERLEQGMEENIKLCLKYSVRHHQLLFE
FHDKLNEVISLPGLGLFVSFSIMLCMSGFIFTLKEVPLVSKSVFGLFLLSECAMLFALCY
FGENIIELSEEIGDALYNSDWAIYSKVMQNYMLIIQMRSRRTMRLTLMDFMDVSRNTFSN
ICSTSYSYLNLMNEFN
>HhalOR4like_isoform_X1
MLKLNDYADSEDEKYFKTGFKKNYGIWLLYGGMFLGNPLLPVAFILSGFILIYFMLGTIT
KFYKTDLLCAIENLHFLIFVTVELVAMMSFLQKRAVLVSMYITIGKGFFDYENTLDDECL
ELKRDAYDKTDSRKRLVHHSFVTVVMSACITISVFRPAVLILFPKENMGNPNDGMIRVAL

APMWSPFDNTQWHGIVIVWLLEYIVAWTTPGIVFGATFFVLFSLEELGIQLQILKKSLTN
VIQRAERLEQGMEENIKLCLKYSVRHHQLLFEFHDKLNEVISLPGLGLFVSFSIMLCMSG
FIFTLKEVPLVSKSVFGLFLLSECAMLFALCYFGENIIELSEEIGDALYNSDWAIYSKVM
QNYMLIIQMRSRRTMRLTLMDFMDVSRNTFSNICSTSYSYLNLMNEFN
>HhalOR4like
MTVKKDELDEEFFYDFRETRKWYKIFAGSSMSKQMKPTIFLEIYPLLTTEYLAEFSEAFH
YFCILLTMAIYIPVERLAKNDIDKSFLKLKKCFYLYNGELNNTQKKIKRQTIEYIKFTDR
VFFWMLIFVCVLYSILTPLKDYFYPHLRLERSEIIDRKLPIPFYIPFEDSYGITFIIVFL
MECCCNMMVHSLITSIHEAYISLTGQIYGEMKLINYSLSHIEERAIKLYLSKQQKRVHRL
KCIYRLPAFQKCFKKCLKEDMIHHQTLLSSIKLVRPFTGRIIISVFTLCSIVWAVDVYVI
SKMIEQASAMDKVLEFLLMLFTEAVYVFDMCHFNEKIRSENDELFMQIYSTPWYNYDRKL
GQNVHLMLMNTIKPTKLNSNLFNVSASFETLMPIISATFSYFNVLRKLKN
>HhalOR49blike
MRKKIDEVKESCVKSLKGFFKTIVFLMVWVSFNLTAMTILVGLIKQEPIYLFPCWHPFDI
NNIIFQILILLWQQYFLSTMIFMAFGGGSMLFIPYTHIKSEISLLKYALKKVEARAHEMA
RTRKASNVGSVKSKVLSECYKECVKMCVEHHIEILGYFYRGKRLTGIIYTTGFFSGVIAC
TFGGYNINSENLALKFKNLAILFFILGYLFAMFWIADATTTEFLTIAQTVFEVKWYELPK
ECQSTLQFMVFMSNQPLFYKLVLGQKVNMEAYMSLVKATYYYLNFLTA
>HhalOR49alike
MMQVMSYAMMLVHQMLMHIKTIITIFDFNMALFSQNVHFCLLIQVSLIVLGTFFRKNFNL
FHIHRLLSIDFYDYHEPSVIGEELLRTTMEKDRRRLTLIPFAVAFAAGIVLTVSPIIDFN
VGSFDFNRTDAIFEYQLPHPYMKYLYYSKDGFGFYFAVFGQMGVGFLLAAIIGGAGFIFI
NLTENISLQLKLLNNSLEHIESRIEHLYTKLFGEMDKDSMNSLQHDSRYDYCFTMCLRKN
FQHHQVILRAFHLLEDTTSLPIGSSYLTGTIVIALSLISTGSANELPGTTIASIILCAVE
VSYMFLFSVVGQRFADLSTDLRNKIYNTRWYMCSKEIKSYLMIFQEMTLKPMTLTGARIV
PANMETFTTVMNGAYTYYNLVVAFDRK
>HhalOR47alike
MQLSEKDYLLKGYQGSQCFLLRIGAMYVLQDENEVYNWIFVGIFHMHYYVFLFWILPYDV
SHTIPMGHMTATLQALHYYCTFLGYSIVNYLYHFNRKSVNFCLTLIGKSFFEYEEGGTEI
EIKAAQESDKNQKEYIKKLTKYVMVIVFATAFAVLMLPPFAELLTRDGPIPDDEIMNPYL
PIPLVLPWDSFSIGGYLLIYVFLFLVSYNICTELLALSMGYTSFLIIYINQFKILNNSVL
KIEERATKRYKKVAVKQLKGMEKFDDPIFQECMILCLKQNIKHHQILLKWMDEACHFLGW
GVLCTIFTTSFLLAASGFLITMESDSSLLKSLILVQVQILELLHAYLFCWWGENLATESA
KLYHSLYKTPWFYCGKRFNKLMQIMMNRASKPVIPRDPLFKINASLEVYMSILSTGYSYF
NLLRSMN
>HhalOR46a_isoform_Blike
MHMKFYGLWYCLNVTRTTGKVSRLFILVCALIVIIASTYVMFQIIYMFTIHLDLQKIAFV
YLIVAPCMQDFYKVIFFLAKMQEICLIYDTLLVDFLESIPKHKMPVVKEIYRRTAKKCNQ
VCSFAFSGLIIAGSIWLFVPGYDTDDPTSDRKKVLNGWYPFHYSESPRYELVYAYECIMT
LWCGGWYCIFECAILMPLICLCGHFDVLSYHIATLKKSDMVHVLGRSSASHLESNAFLND
QLKYILKDYEKLIRYGDTIRETYNLVITIILGVEIGNLTVIVLHLIFEDKDAMFLVKTGT
YMSFQLIEVILICFSSDMMGEASSGIREALYCNEWYTTDRKLATSQQLMMVRASVPLTLT
AVKMYPVNLETLLSIFQFIYSTAALLSKMK
>HhalOR43blike

MVKYIRSLLGDLPTVLDKTFLISIDSDKRFKQIFFTICNAIMTTACVGSMYFLGLEKSLE
GAAIFSALSTTISVKQLIYFFRLAQVKKLLYTLKKLQNHHRETWEIEMFEAGSVDTWNAV
HTFCLTLLCYLMLFLVLSAVLDFTIGIIFPRAPSLLVQLPGQGFIDFFEPRTLEWLLVTT
LFLMWAFEAMVIHIGTESLTFVPIMYVKIELKILRHKLLLFKEELEKLGKNTRHADKLLT
DIILHHQRTIEVLNVMKKTLGLPLAVQNTAFSITLCFNFYCIITFNEGGSLAVKFNGVLV
VICIGLLLFGLCFFGESLEKENHEVLNCIYDLAWYLQDKNFRRSVLTMLRQAQRPYVINY
RRIANLNLTAFMQIINTSYSYLMMLKSTV
>HhalOR43alike
MFITTYQILYSYRLKEITDKMDSIGKKIMENDLGGKDFFKQEYVKNAKFLSIFTRCSMTS
IFTTPFSYFLSVPVVEWFEGNYREHLPLPLANVFDDRQPVVYEIVVIVLSAGISIATAKK
AALDSLFISFLSIQTTFLKYLSVAKDEMSKEVRFADDGRSRRKLLTWVKLHQEVIKNIEE
LVEYFSPIVVVYYIVVIEIVVCGAFVELKKDNDSIVQSISVGSYVMLTVIFYYLLSNKAD
ELTTEVQKMVAAEYNLPWYAMKKSEVSIIKVVLMMCNKPIHITAYQAPVLRLNRETFSQF
IVRAISALVTFFQMKDIFG
>HhalOR33alike
MVYLQRLQKSDLFDGLNIGHLKFYGLWNGINDYRSTRKTSCIFKFNMTVSALYVFPFVVF
QFICIFIISVDLKMATFVYMNGVSAAQVLFKIIVFWYRFKDQCDLVDLLRVDFLSSIPDS
KTRHVNEIYKKNSLRCNIFTILAFTGNVLTIITWTILPGFNTEKTGTGRKKILSGWYPVT
YSESPWYEIVFVYEVILICWHGSLVSLYESFLLMLLVGLYSHFVVLGYHLSTLKKNDKAV
VKAGVDTKIDEAFNIELKKIMQDYNKLLRYSTLLRTTYNAITTVTLGLDIGVLILTIMFL
MFGSSDGLSTFKMMMYFSFALIEITLLCVTSSIVGSASMSIHDSAYSSDWYVADKKFATT
AQMIMIRSMIPVSLTALKMYPVNMETMIGIIRFIYSAVAIVSKMKE
>HhalOR30alike
MEAMRDSDVIDGLSIRYLKIFGIWKIINDYRTTGNKNMILKIQVIITVMVTLPSILSQFL
ALLVIEVDIQKATILNFHTLPPLQALCKHLVFWFNIDSISRLYNLMRKDFLEKSVNDIER
EKVDFIFRKFSKETNRTCFLVFMVINVAGSFILLFPGISVDYILYHTGKLSAVTTGRRKL
STGWYPLPMDTSPCYEIVILYEGFFVTWCGYSILVFMCLYYQLLKCLHAQFALIVSHVST
LKIDYEYEKNSIEYRFTNQQVYSRMYRILQDHQKLLSYAAELRSVYNPLVTMILGVGMSL
LVIAVFQFLFGQTGDLLFMVRAFFYVLYQCIEVSMFCLGSLFVQTASSDLHSAIYSSDWY
KADVKFRKAVQMMMIRAKKGETLTAIRMYPVNAETIMAILQFTYTVATLMSRFTE
>HhalOR24like_isoform_X4
MRNRTLSEAWVTTSVFSIKPYTKVSVNKYFLEIKPLIILQRALGKLPYSFNKHGFDPFKI
ISFPVLYTIVFFTVQSAWTIHTMSVIIKEKIFNAPSYDMALFWVSLELILLLNIASPITE
WIDVHKYVQFVNNWKDFQNGCRQMSTHRKTWLKLSKLVSEVGDSNAHTGIIMSVTYFTSL
VVTTYALLSSFSRLADYNSHFWGHLVSTLIGFLSNFVLCDAAHRTTQKVNVKLGPEFSSK
ILAMDMTHLSQSEVNEICLLLQTMSAHPPLIGYLGFVTINRNLFVSFMSNAVTYLVVLVQ
FKSTSPLNPIKEDITQ
>HhalOR24like_isoform_X3
MRNRTLSEAWVTTSVFSIKPYTKVSVNKYFLEIKPLIILQRALGKLPYSFNKHGFDPFKI
ISFPVLYTIVFFTVQSAWTIHTMSVIIKEKIFNAPSYDMALFWVSLELILLLNIASPITE
WIDVHKYVQFVNNWKDFQDNYMDSELEMTLSLPIKIIAVLLLPVALAFVSFQVYALTDLT
FFTMLPFIITACSSTITLVQWCATLYELRAATRVLLSKITMNGCRQMSTHRKTWLKLSKL
VSEVGDSNAHTGIIMSVTYFTSLVVTTYALLSSFSRLADYNSHFWGHLVSTLIGFLSNFV
LCDAAHRTTQKVNVKLGPEFSSKILAMDMTHLSQSEVNEIAMTDSPINLNVNLVSVEMAF

LASFLL
>HhalOR24like_isoform_X2
MRNRTLSEAWVTTSVFSIKPYTKVSVNKYFLEIKPLIILQRALGKLPYSFNKHGFDPFKI
ISFPVLYTIVFFTVQSAWTIHTMSVIIKEKIFNAPSYDMALFWVSLELILLLNIASPITE
WIDVHKYVQFVNNWKDFQDNYMDSELEMTLSLPIKIIAVLLLPVALAFVSFQVYALTDLT
FFTMLPFIITACSSTITLVQWCATLYELRAATRVLLSKITMNGCRQMSTHRKTWLKLSKL
VSEVGDSNAHTGIIMSVTYFTSLVVTTYALLSSFSRLADYNSHFWGHLVSTLIGFLSNFV
LCDAAHRTTQKLGPEFSSKILAMDMTHLSQSEVNEICLLLQTMSAHPPLIGYLGFVTINR
NLFVSFMSNAVTYLVVLVQFKSTSPLNPIKEDITQ
>HhalOR24like_isoform_X1
MRNRTLSEAWVTTSVFSIKPYTKVSVNKYFLEIKPLIILQRALGKLPYSFNKHGFDPFKI
ISFPVLYTIVFFTVQSAWTIHTMSVIIKEKIFNAPSYDMALFWVSLELILLLNIASPITE
WIDVHKYVQFVNNWKDFQDNYMDSELEMTLSLPIKIIAVLLLPVALAFVSFQVYALTDLT
FFTMLPFIITACSSTITLVQWCATLYELRAATRVLLSKITMNGCRQMSTHRKTWLKLSKL
VSEVGDSNAHTGIIMSVTYFTSLVVTTYALLSSFSRLADYNSHFWGHLVSTLIGFLSNFV
LCDAAHRTTQKVNVKLGPEFSSKILAMDMTHLSQSEVNEICLLLQTMSAHPPLIGYLGFV
TINRNLFVSFMSNAVTYLVVLVQFKSTSPLNPIKEDITQ
>HhalOR24like_isoform_X1(2)
MKQGSMSKAWITTSVSHNKPYKKVPVKKYFQEIKPLILLQRAFGKLPYSFNEEGFAPFKL
LSFPVIYTIIFIVFQSTWTVYSLCIIIQEKIHKAPSYDVTLYWVSIGLFLLLNFTTPMTK
WIDIRKFVHHVSSWQDFQNNHLDAELGANLSLTLMIISVLLLPIASVFVYCQSYLLTDLS
LFVMVPYIFSFIETGVIIIHWGVVLYELRIASRTLLSKIIMDGCRQMSTYRRTWLELSKL
VSGVGESLGHTGLVISIVLFTTFVLAMYALLSSLFEPAKTCNHVWGLLINAVLSLLCNLF
LFNAAHRTTQEVGPDFSCKILASDLTHLSQVEMNEISLMVQTISANPPTVEYLGFVTVNR
SLFVSLVSNAVTYLVVLIQFKASAPEKPVKEEVVQ
>HhalOR24alike_isoform_X1
MKYLKFFGLWKIINDYRTTRKKNSILKFKVITTLFLTIPYIVSQYLSYWMIEVDIQKATF
LNLHSLPALQICCKVLVLWFRIDSQSRLFNLLKKDFFGIPKSKEGEAKSIFSKMTSECNK
LCSAAFLINTSVVILSIIDPGISVDYIMYHTGNMHAVTSGKKKILGGWYPLPIDKSPYYE
AVFVYEILLIIWGGILLAVYVCLFYQVLMCLYAQFSVLALQVSTLKYSYIQDGKGRESVN
SKLYKELYEVIKEHQKILRYAEELRSVYNPLVTMILGVGIFVLIIAVFQFLFGSTGNPMF
IFRSLQFLAYQGIEVSMFCFGSSYIQNASSDLHFAIYSSDWYKADVKFRKAAQMMMIRAK
KGVTLTATRIYPVNVETIMAMLQFTYSVSALMSRMTE
>HhalOR24alike
MMSDQAIKDSDVLDGLNVKYLKFFGLWRVVNDFRTTGKRNKILRVKIFITLVLVLPYILC
QYLSYFVIKVDIQKAIFLNLHLLPGTQICCKIVVFWFKIESQCKLFDLLKKDFLSVPEEM
RPEAAEIFKKITRRTNKLCLAAFIVNISIIISSIADPAISVDYILYHTGDMAAVTTGKKK
MLGGWYPVPMAETPYYELIYVYEAVAGTLGGFLLAMYVCLFYQVLMCLYAQFTILCLKTS
ALKIKSDNGRINSSIYKELNEILKEHQKLLSYAKELRSVYNPLVTLIIGIGLFILIIAIF
QFLFGGKSDFMFIFKSLQLLVYQCVEVSMFCFGSTYIETASSDLQFAIYSSDWYMTGMKF
RKEAQMMMIRATKGETLTALRVYPINVETIMSILHFTYSASAVVSRMAE
>HhalOR24a
MQYQQPLRGPDVIDGLSIWYLKLFGFWKIINDFRTTGKRNLFFKFEFIMSILISFPYIAC
QFSSYLTIDVDIQKATLINFYCLPAVTMCSRILVFWFHADSQCRLFNLIKKDFLCIPENK

KAETRKIYRRVSKSCNMMCMFAFVLDLSVVFTTVGIPGIPVDYILYHTGSMFDVTTGRKK
ILCAWYPLPMAEYPYYEIIFVYEMMCVLLGGIYLPIYASLFYQVAVALHAQFLVLGYHVS
TLKINPNIKQKKKNMSSGITEDLYKILLDHQKLLSYADELRSVYNPLVTINLGGAIGILI
VSVFQSHMGETRDIVFVLKSILYAASIMIELLMFCYSSSLIQAASSDLHFAIYSSDWYKA
DTKFRNTAQMMMVRAKKGVNLTAIRMYPVNLETLMSIFQFAYSTSALMSGMLEE
>HhalOR22like_isoform_X2
MKQGSMSKAWITTSVSHNKPYKKVPVKKYFQEIKPLILLQRAFGKLPYSFNEEGFAPFKL
LSFPVIYTIIFIVFQSTWTVYSLCIIIQEKIHKAPSYDVTLYWVSIGLFLLLNFTTPMTK
WIDIRKFVHHVSSWQDFQDGCRQMSTYRRTWLELSKLVSGVGESLGHTGLVISIVLFTTF
VLAMYALLSSLFEPAKTCNHVWGLLINAVLSLLCNLFLFNAAHRTTQEVGPDFSCKILAS
DLTHLSQVEMNEISLMVQTISANPPTVEYLGFVTVNRSLFVSLVSNAVTYLVVLIQFKAS
APEKPVKEEVVQ
>HhalOR22clike
MKLLISDYDVEIDGVIEKTYSGLNTISAIYPVMDMERPRLCLINLFLFLFHSVILTVMTS
SLVQASLELFYINFVPFVHELHLAFLGLLSIAILWHSWLKRSNVTRLHRIITQGFFDYKE
NLEDKMKVLGEDRIKERKRHILYVILIGFAASVVVILIPAVNQFGTFRYNSTAYKVNFDL
PVPLPYPFMGTEPLDLLPGYTMIVVTASSIALMNCTKSFMAIDCNLHIQMQLKLLLHQIE
IIKSRAGRLYIKLYGTEPKYNGLKLYDKKFMKCYRICLRRSIQHHQIIVRALNEFNEVFG
FYVFFYYLTGTLDIAMSMLATSSTKEFPGTTIGALIICIVEVGFVFVFAHMGQNITDLSV
ELREVIYDMPWYRCDQETKTTMRVLQIATLKPLSFSFYSLLHINYDSFATVLHSAYSYYN
LVNAKN
>AlucOR1
MLPGFILLVANCVIMVPYISKWVVGMKNPYTPRGVNLNLPVACWLPFHSHTGFWHVVAVS
NQLIAVSCLAVIIITLLFMFLKFSQKVRYELKVLHYGIETLFKRSKRLYFKMYPERKAIR
FHWTDPVYQRVVGICLKDSILHHKTIVNILDVFMTMVSIPAALAYVIGTAVIGLSLLSIL
NALNQGNYPNVILFALLCVGEILNMLVASLIGETLTHETIILREELYFIEWHKLNLSNRK
TMLNFQTAITEPLSMKAAGLVDMNMDTFSSIMNSAYSFFNLVNAQ
>AlucOR2
MLLCWKKKKEKQKPVIKEQHDFANNYRLFKYIGMIQDGSLFSRIRVFLATFLLFYAWFHH
LIPLVNSTEEYSFDELMDLIHLEMVYFLWCIVWPSYIIRAPLFTSLASKIQNGLYTYSDP
LTLEEKTILSTANDAVVRMTKISVYVYVCGGIGIFLKGMNKERMRKLQLPNIGWFPFAIN
SLSRYAIGCLCQAIMGINAISIAIGTFMSFAIFLIHYEAQFKLLRTHLKRSFPKNVPLRI
AQTDKYKKVSLRRLKDCYRHHLAILGFHQEIMKYYGILLLVFRVAIVMWMCTLAYVTVMV
DVNAHNLLKMLSFASTELLYVFLFSFRGQDVTDWNYQWREELYSIQWWEQPKEVKTNIGI
MVLGTTQPLLLYGVWKIALYSHEKLSDIGNESFSFFNMLRAIN
>AlucOR3
MSAQTIQSDGGRALLNRDDVKGLNMGLNTFGAKTFWHTLEHFHATGKRHWVMATYIVLLH
LVGFTYCLLGFSAVFFIKMDIKRGTAAIMNPICGLQTVFKCWTFSWSTAEYLKLFEILKK
DFLTCVPPQKEGGANDVLAKNVVATNEFVKNAMRWNFLTLCMVSTMPYLRSQAFREFFRL
GEGAIVPNKICENEYPFEWNSTPIYEIIWIYEQIAVILAIVTSSAYQAILLFLVMALVGH
LRVLGYVMENLRASDFRGETYQLMDKSAKANAYQQLIRCIRDHQKINAAGDALAERYNFF
LTFHLGTAIIVGIIAIFNCTAADELADKIKFAIMCGYGLLEVAIYCYCGQLLENASEDVL
RQVYQCEWEEMEPKFRKAAQLMMVRANNPIALRAGRLYRVNLETLGAIQQLVYSALTMLS
SMIDGS

>AlucOR4
MVLVPYLKPQKERNAAVDRGYDITGMFYARLAGLYPDLEIGWRYWFFGSYQILVVVAYFY
YVLAYVIANVIAIKYMDVELIGSTLCFGSYTYTYALIALTFYIKRSKIDKLLEIIGNELY
IYQCPLSQKQLKIRNEETTRAKNFGRYSFFVPCLVALTHMSVVPAIHGFKGEYSSIVNGS
APINKYTPLPVWTPVQATSGMSFFVVFWCQLCPGFVEFLIFHGSCTFFVGVVCVLVSEIK
ILLESLNSITDRAKYLYHVKGGRGSDIDNLYDDPIYQQCMVDCLKENVKHHIKIKEFRNL
FQDIISYCIFFIFGGAAVTISTPPYTILKIMESGDTDKLYSAGVVMMGHTFLSVYLLSRY
CKFGQNFESENSKLLEAFYCTPWYNTNMDYRKILIIAMSNSQKTLQIKGSVVGVSLSAAA
FLDVIKSSYSLLNFLATAGS
>AlucOR5
MKIVTSDSNVQPVGSTSESKMVERKPQNLTKQLSLSRNPVRTRKERQKRTKTIYDQLDTL
FHVGVRCLLLGISPDKFRGNKGYERFLIYFVVILFFFAYSLFQLCAPYFFPGDFLQGMRH
FYFGSYCLTFGYQWIYLLRNFDKINQNRVNLKCFNSSRAVTEIAEVILEGKLKPFTRRIV
CIAMIFSIASFSHGLGELIQFGEQYFIMGEVKTMLTYFPFPIWGQLIIVLLNTITISLLL
AGTVSALIITGLLTLEIETQCAVLCAAMVRGTDDGSQSFTGLIADHVQIIKNARWLIDLM
ESLNSPAFSCFYFHIAIGLVIMKLVETNGYLFYVSMAFGFMSVILQTGAQGWFSSAITHS
LESIAFAAYETNWYERDKNYARDVLMVIQMGQLRFVQKIFFRSIEIDRATVLRIVRSSYS
FYTILMIIQ
>AlucOR7
MLFGAGSKELGFRKETIEEEDVGFMQLAGLYPLTRGYTAYYFISFILCTTLMEGMIVGSY
LEGEVDTSLETAHVLLISLNMFTQICTHRYYYDIVNKLLRAIDDNFFSYGDTMDEDTKQV
IRKLAKEKTARKKMFGKVFKIQVSSAAIAITFQRPILYVLNGRGVKDVDGENWLIYQSPF
GILVPFSNYWVPYLFGMVLVVNQVITTSITAMATATSFVRFSEELLHQLEIVKLGLGNFM
FRARHLHSLRYNQSKESGEQDKHLDKCIITCLSKSVEHHAIIIKLFGDFKNMMYIPLFTV
IFDGSVLICMSAVQLITSKNPIVRMSMPPFIVAELYYTYLYCSYAEKLTNMVLRTAYSLL
NFLATRK
>AlucOR9
MNLIGLPPREDDRKAVDYRKCTFFKILYTENETGGKVSMARGVFVIVTLSIMATCCLVSV
TRSQTAEQLLDNLKGMHLEVMVLMVAINECVSRKRMRRFMSYVERFRANPRYDLPGEEAI
LVQARSNAIRDITFLVIIFGANFPLMMLTKPVTEALGGGSWKQLPFPWTVLPDDEDTTFV
AFLLLHTLGVFFSHCLGVVGMCFSTITTQITALFDVLLLSIERIEERAARKMKQLGLSYQ
ESMLICLKESVAHHQELVQEVRSEKPHLESQFFSEIVNISTIMACEAFPLVRPNLTISIA
IKGLVFLVVQVLCTAVLCDRMEIMADQNTEVFAALYNTPWYKCGVEYRRILLNGMTFCRH
PLTIRGKSFLGLIATRATFYTAMVNTFNLLSMIRKMG
>AlucOR10
MDRHEGPLSTHFRRLFRLVGVYNGKYITPHSCLFFFSGFVNIIYLSYILIFTDCNMTKVA
HFIILQYFYIGTLWTVVYKGNDIIWIANECDKFVGLDGHHFERLYDEVREQEKTSPATKG
KTIIDRIASIICTWTCIEPFINAWTGKAELEFPFTGTNADTTKFIFIYLMQCSLMFIVAI
VCSVIFKSLMGIALDLVIKYKVIGLVLSSLNEQMMNHRNVYKSDLHQTIRKCVQSHHHVL
RIFEKYRDICTYGFRYSYVGLIGATSLSRSLLSSDDPDLGTIPHIIAELSYMGFFCYILN
QVEQEHDKLKDAVFAADWPWLPKPATSSLRLIMMRTAKTPRVILVKGGGPANLETFYKLL
NGTCGYLIFGLVLDQAAF
>AlucOR11
MFRGKEKERPYVIKDPKDFDTDRLVFLWLGFVYDGSFFSKVRLTVFVLLLYSAPIHHMLP

VILDKSTTTDEVLIALSINMLYVLLCIAWPFMIYRSPEIIRLWSTVRQGFFHYSDPLTSY
ERTILSKANDLIIKSTRMSLIAYFCAGFGTYLKEMSPNSMRLYNPPYPGWFPWTINSNFR
FAMALLYQLSICLNTTFALEGIFLLFLFHTISFEGQISLLKQHFEDTFPPGLPTEMTHVP
AFKERTLKRLKECVRHHLVIMDFHKRILSYFGICLLVYRAICTIMLCILCYLTTTGIALN
KFLQLACLAALILYLLFIFCLKGQKVSKMSEIWRETLYEVDWWNHPVEVQKAVLLMLVGA
GKTMTVYGVWTPAMYSHEGISAIGQETFSFFNMLRAMK
>AlucOR13
MAFIMRLIDKCAAMENDDLDRLLDNYYGPMFKLGLVFPSWKRSALVFTIPWFILNVSTFT
WNLILLGITVYKAFLCDNDMDLFSLSTHYFLLLLCGSLIIFFMNWNRKLNGLNHTRISVD
VGKYKDSRLYSHKDCILMEKQIRVESYRYLCLPLLIVLICGAVLIVPYASKLFRGVGTMY
TTCGVDMFLPIPLYHPFPTHEGLNHFLALISQVLVVCCLANVIVAIMLNFTQYSLRVKLE
YQVLGYSLDTLFARSKKVYLKNYPNEKASFHIRNPDYQRIVGSLLRDSIVHHQTLVDMMD
KYHGLITYPVVFAYLTGSGAIGLGLLSIVRALQKGDTETLLLFSLLMLGEVISMLTMSLI
GESVTEATIMLRYKLYDIRWYDMDIPNRRSLLNFQTFITEPLVLTAGKGLVNLTMETFSS
IMNSAYSFFNLVNIQQSE
>AlucOR14
MLTVGSSAHNSLSRILEVIGITRYKEASFFSENSFKLFRVVQWALNVLMIVSCLNFIFSE
SVDDSPDKLQSLALCTADIQFLTTQLILISRQSLVDDLVAHLRSVYHGIKEIPGSMDILA
EGDRQARLFIMSYGVVIFSNVPSSMFFAGIKMILTGETAYPFPMSIFGLPTAVGWALQML
MIANAANILWGFYCVLKTVIYILGAYSNVMAHMLRERPIDVEAKNDRKMLKLHCDINSLS
LKLVNVYGLISFLEVTVASGRCCFVAYHILLAVQEGDYKNLGVALSTLLTSVAITYVLCS
CGEEISMQSVTIRDGVRDSKWYAVSPPARKTLLPVLLFTQRPIQFHYRRFVYFNLETFRN
VLKTAYTMTTALAQV
>AlucOR16
MTLLSFFKDKFKWNEPLGITETTATLFGAFVNYAPSPGYRKFFRFFGWYIIFMFIIFNIN
VVLTIYFASDFFEESLEAVRLFVTAIHILAKLLTMRAMEKQYMELIEQIRRAWRTYEYSS
GDMLNKTLAAANKGTVIVFVAIGNTIPINVIVAALKNLGNPPEIQFSMQCWVPPSLRTSF
LAGSFYQLTPYFFPVMLYCMTISFLNSITLHVEALGLALAKEIRSQKEWRDEAARSLYIK
HQEVVRIVGRVNDLMASNWGFEMMCATLQLTLVSYNALRTLKKNDVAFFNQANLMLVNFL
VIYFIYGNGNRIIKMGEELHNSLYDTKWYTSTVKERKNVLFMMFRTSMPMEYRFKIAHFD
LPSFAKLVNTVFSYITLLRSVDEPEEQGAF
>AlucOR17
MSKLKNLPFITILWREFKFLSVMGGTYGFYHTKAWTAVTVINYIVMYSAMLFTMSVLVYT
TYLLRDNIGYMSQALHLLIVGCVTTTASFTITINRYKVEKFVVFFEDPWSLCEYSRNDFF
EELMKETQKKKTRLIVTWILIYGICGVIGLLQSGINTVFGTQSELTDVNGAWVILPFIMW
WPEDITASTGAWMRAFLVQSLYLYFCVIMVISGVVFAFSAVERILDQVKLLIYGIKTLDR
RAKDMFQQKFPGSDMKYMEKEYDDCYYECLVQNVKHHHKMIKWIETFLDMASLPIAVPFY
GGAVLLGMALITITEKDDPRVGPKCLAACLAFSEAYNMYLLCDVGQRLENLSQELYDTLY
FSRWHTRSPKVKRAIQIMKIGCQKPIVFTAAKLLVLNMSLFADLVNSAYSIFNLKAASEK
FEDK
>AlucOR109
MKRELKAEAVRHFTRLVVKKPMDLLPRLHRGRGVFNLEETSSGRSDSESKLPRAQHNVFY
QQFRPMILVLTAFGRLAIQRGSDGEYRWKWFSWLSLFCLLNYAVQTYFAVAICRQRIKAV
FESSNYDEFIFAIHILAYIQMHFHVPVSYWVQGPLYAQYLNQWSQFQNEWFLVTGEELKF

RHKKAALTFVLVMLPFLALVLVMEKYSTLHDPFEYLLPHFFTIGSTVCVLGTWYIACLEI
AFISKDLTKHLIKKLHSNPDPTFLQKWRALWMNLGSLVTNLGTNHFAIMSSIFLTFSVTF
LLGLYNALSKIIIGDFSLKTIGYLTASGMSIIIIYIVCDSGHQATSRVESYTSQCILRAH
LPAARDDVKYEVDLILRVVQTDPPQIQLAGFVTLNRPLFISFVANTITYLIVILQFKG
>AlucOR20
MVIQVRNMEQGLKEITKQYYAAAGISGISAYLKNDKPFFLIRLWVWISTWIILPQVTLAH
IYTALFTGIPLATRFLSLSLGLDQLQTTIKCHYMLHNLNRFRSIMLDLETFNCVNHLGTE
ALTTMLKMCKLVKWLRSTYNFACYFTFIAWTLVPVVSTPKALFYAEDGVSMMKVLPPEYP
FSTDYFPMVQLIYALESFSTFVILTYFASTNLIMVTDILLICQLFRVLNESVAPSKGRKA
MTLRMFAVDHQKLLKICAEVRDLLSPLLALQLGISVMTITLAVFEITMVNQASSEGGINQ
VVLMSRKTSYTFIIFVELLLYCWLSTELELSCLSVRNGLYNSEWYKYEQLGTKDYRNYIM
ICMRALKPVRLTAMNIVTLSLTTSMEVLRMAYSYYTYLKKLR
>AlucOR21
MFLVFMVTVGILFQGAKLLWNKNETTFDLLFSLVHGRENHCTYTGTPYDEFLKSRRHTFL
QYGPTLFTYTNITVMSMYVWLPPLVDCIMGVTHHPNDPHNGIATTLGLPMWTPLDADHSW
TSYFIVIYMQITFFRIVTVSWAFGIFYTSIFQLTLLDEMRLLRKSLQEIDTRATQLFTLK
YQRQPVNRKTKEYDDCYYECLKENIIHHQFIRKIYGEYQKRVGWTIAIPIYLSSVVLALS
SSYIMLDNRNALKRSMDVLRIIGDSLLTFLTCHAGEMIAYENDELRNELYNTEWYDRSKK
VKQAISICLHITYMPMRLYGGYMVFNHELLSTIVNSAYSVFNFFRVIQKSAK
>AlucOR22
MIRLRLPPTINQDTSQKNIKTIYEELDSQFHTGIRFLLMGASPEKFLGKLSLTYVLIYLS
IILFFMLYALFEIIVPFFFPGDFLEFMNHIYFGSYCLAFGYQWIYLLMNLNNIYLNRLNI
ESLYSTQAVSGIAEAALRKNLRPFKIAIKCSIALWSITILAFGLGSLIEIAVEYLLTGEV
ESYAIMSTFPFPPWGQMLVTALNFVTLNMGFAEVVAMAYISSLLALEIETQCEILCAAML
QDRDDWFKFRGYISDHARIIKNAKWLIGILESLNAPTVFSAYILMAIEMVVLTLVEPDGL
FFVAMASDLMGVVIILIFQGWISSKITLSLQSISFAAYQTSWHEQDKNKALDLMIVTQMA
QRTYVQKILLGTVVIERGTVLQIARSAYSFYTLLMVLQSNKTL
>AlucOR23
MWLKYPSDEELNRLGICRSTFDGNVFFKAQLGGLCWFSNSWSVFYFISTIFGIFSGCGFA
YGVFMEKEWEALYEALHYIPLVVNITSTAASYHYTQDEYLQVFRSIDKEMFDYEGTLDEQ
AVEEIARMKTEARARKKKISMIYTKLMIVAFICQTLRKPLNYIIDGRGKKDVDGENNLIW
DVCPFGIYMPYADYWAPYLIGHFLCWSCSAFSAITAVASALTYQAICEELLADYSALDLT
LSTIVQRAERLFSNMNRGFTGNASGTVTFDYCLEKCLKVSIKHHHEIIRLFNIVKKLLYI
PLFFTIFDTGIVMCFSGFIMISDDFSPKFKLLLSPVMLAESGIAFMFCYYGEELTEMNKN
IGNRIYFSQDWMKHFKSIKPYALTVKSYCDIPNELSAGGFTKVNRLAFSNILSAAYSYVG
LLLTTS
>AlucOR24
MISLTIGVRKQLQSTTNIMDNMFSFFAATPSFLGMGKIICLLRQRRALRRIWKSLDDLLE
NVLKRDVDEQLEKELRWRLKRCWAMYSIFLTVGTCITLHWLLRPIVYALYGERTSIVSTW
PTYLESWIQWFTTYIFQAMNISSIGHALYIYDNVYFCICENLLIHFAVIKHHLHQMDISK
GKPGGVTMKFCISHHVKLMDICMELRECSKYVIMQQVFWTIFILCPGVFELVSGRQTDTT
ILFNLMEITTIMTCILFFYSWYSNEVTLQSSQVFNTCYMSNWVEGTPNQRRTMMTMMTRS
MKPMIFGGLVNVDLGTFISCFFRC
>AlucOR25

MSFLFKFFAYLAEGEDDELWDTYYNFYGPFLEISFVFPSWRRSRIPVSLTVLGFFAFTFP
VHLWLLTLGIEGVRDDFNLASMEFHYWMLLMFSMISIFLMNSHRQNMIDFHRRLSRDVGF
HGPGRIYDEDYPMKLEHNKRKQLLQFLFLPGLVLVLAGATLILPYLSKMDGTVLYNSREV
NMKLPIPLWYPFPTHEGILSIMAVLGQFMAAGGLSAVIMTLDLIIFRATQAMIFEYKVLR
YAIDTLVPRAKRLYAQEYPMMDLESVKMSDDAFQICIGKCLRACVIHHQDINRLLNLYKV
MLKWPGFMAYGFGTAVIGLSLINILSAKQNGDYENIVLFFGLSMAEVLNMLMMSVFGELI
STESKELRQELYFIEWHNLNTFNRKLMLGFQMGLNNPVIVKVGGLVTVSLETFSSIMNTS
YSFFNLVNAQ
>AlucOR26
MGYVWSKLQLKIKLWDSWEDEFSIDVMRHRYRGFHRIGFIVLDLSSKYAKLSIITALLGT
SFLLMATLSLLATCAKMSDDFEECSGVCNLGFLSTLALAFLVNHNYFRKTILSAHHMLGK
GFHDYQEPEYSSTEFEKYKTMLRKQNVALIILACYVALIGILVVVVCPLIDESLGFGWTE
PYDEYGVNRQLPVTLWLPFYSHEGINHWFTFLFIEGLGGAMICLSIGGSALLFTSLTIGT
MLEQKRLVLSIRDIEQRARHRFQTQYKGKPGVDDEGKNVAL
>AlucOR27
MDGNIERHFDHMEKFLKYYWQWYGYPDTFKGRLITIFNIFRIFLLMVLVGIAASQAYFYG
MSYLVDGSAIFLPLGIMSLVLNSHQSWNFGSIVKTAKQFELFLSSFQDEEERNLIASRMK
ERKRTVLQIVLIIEFYLMAPLMCHAISMTLHYYGFLKKPVLIPLLFEMFLEGNYELGPKL
IATAVVSVFYVHLVANIVTMILLNVHFLGLVVACLEVLTERLKGFAEKTQEGYGKLKEEL
DLRDTIQQHADLLHIINSFNSWNGFLVTFCLAACSITFCLDALTTKRALEQEIYSGACLW
GSFLLVMMVLSYLICDSGSQIETKSEELLRAVYNLPWYRGSSETRKAVWMMLTQGNRLII
LNYKELMDLNMVTYLEMLKRAYSYFMILSSIE
>AlucOR29
MTEAEVKDGTKKVDDKLGCIDYRKYTFARMIMIDDGLAARGLTFPLLLIMVVNVGMQTCS
FISIFTSTQTSVSLDNIRACLLGTSVTMSLFNQFISRQAIARLHGFFDKSFRTISTNPNF
PEEKIILDDARKAASSQLEMYVKMFSCNAVAMMFAQPLGELMSGHSWRKLPVQWTFPPSD
DEFSFWLIFAFQFTGICIAHCVGIMIMSFTSITIQMTALFDLLIFSIQHIEERATARGRQ
SGGDRHACLLACLTDDVDFYQQLIREMDSVTPHLRNTFLIISAAVPMVLACEAYPIMQGN
FSFADLVKSFLFLSIQILCWAQTCSRLGTMTDKHAAVFAALYDSPWYEAGMKYKKLVLNS
LTYAVQPKYIKARLSNEVTASMATFYSFVMSAFNLLNMIRNIG
>AlucOR31
MNGNGAVLNGVTESSMPIKRPRKLKKVPNKRSIQFETDRNEKKVEKFLSDEEALKKGFDE
NQGIYLVLGTLYRTSLWSWVHTVLFSLTALFMMVCLGRVAVLISDDFSLLFETIHYITII
GGVLVILPPMMRDEFRFEKIFKTFGRNVYSYDMLDEETAQSVQKLRAQGNREKQLLTKAF
TVMLLGTFAGFSVLLPGMYIINGKFFERQRDDGIIMGIPCVLWFPARVGDDWIIFVRVFL
LLIEEYAAFTVVAFIIGQQTSAICIGHTLLYEFKVLSLTMEKFVRRATKLAEGKKFEGIR
INSEKQLYEKLTACLKDSVKHHDILLDVSEQYKSIFYVPELVILLSSTMVICLSAISLTS
DDIPLEAKALSLILTGAEMVNVFVNCYYGQVLLDAHDELGDAIYGSGWTSCSTTVRQHIL
IILSRVQRPLSLSAGGFAAVNLDTFAQVVKSSFSYFSLLQALKE
>AlucOR32
MSGDISRFKAKSTTTNESLMREEYIRKGIDENNGLFLIIGGMYTGYLPISILHAILTAVH
IPLLLAAVIIGRNDYVVVSETIHFIILLSLAFVISMRYLSVRKKLDNIFEAMGRGYYNYE
GTLDPGTEKEFAIHLKESEKRKSVLKYVFVGGCIGALICVSILRPVLQYYLKKYIKSKKL
PHGLNGAKNTFIYYPWDSSNTWLNFIGYFLQDAYTLMTANVVFGFVLMFVSTAESVVVQL

DKLKLSLKRIKIRAAFIASINHEDPATNNNKDFRRALHICIKHSIKHHQLISRIFDDFKS
INYLLLFYLIGSLTFLLCMSAVLFAADDVSLISKATFVFFITSELVATFLVCMYGEHIAG
MSSSLPMDLYNTEWYHFSNELLIYYRMLAMRCTRPCQLTAGGFSQINRNTFLEVLKTAFS
YANLLQASKQK
>AlucOR34
MVTVAVLTLTYVFTHEVVTQTDFIIAINSYIVSMTFCAAAFKILIFLVLQKEFKKLFLMV
EELGDLDMYAPSTKDHFFNCYMYVTLVLTNPCTWSLWHLIAHNDIPFKSQYPWGDDGVGY
LLSFFFGIMAAVFCGLSHILVDTSFMMVIAGITLHVDKLSQSLSLLGKHRFKDSKIMSAG
IDKHAQLLRVSQHLSTCYSNLFVGQSVYTVGHSCVLLFGAVHVESKVEVVMSLGTMLVTS
YCQLLVYCYYGELLTSKFSDLVFDSYNNAWYDSDLQVKRALPKFSLMCHRHVSLRGFGKV
IPSKSNWLHSLQESVSYFLFLKTISGEE
>AlucOR35
MGLLFGRTPFYIKSGWLRFSYKSLPFVYALILAFANWFCLLYYIDHLHNTWNQTLGSNVS
FSGVLFAILVFSQPFSTIFMVYSWAFEVPAIVRGYNSTAVLEEKISIVFPLYKQKPSKRN
LVTFLIAFLFVVDLIIAYFLLKTRFTETPLILILLIIVNVVVTFSYCVLWCFNCYFISDL
AVKLNKYMLQCLQVRENCAFKIKTCRKIWISVWKQSQMNSQSIAVSLSFALVLYGMIFVV
GCYGILTSIRNQNILETLEMSPYVVVTFTIIACVFETSYQASHKLGATFLDTMIILDKDR
VDHECVEEIDKFVDTINRTRIAAITLKDYMTMDRTLVVSFLSYSITYLIVLIQFQDKNEE
PSVNISTPMRNNTL
>AlucOR36
MLTEYNLRFLKVLHYYGFWITFVGWKHDKIKKICLPVRGVVIGLSSILAIYKLGQEGIQC
IINGTVVYFPFMVYTFTIPILLFKNRKHFLSLLLDFEDCWKFFCEEKERKVLERHYKRIW
KVANYVHICFFFEIAFYAVMQLALDSVLHYVFDYLSKPHVLTYPHMGYLPTNRTWDGLYY
VVAVVGCYNLVETLSVQLGWVILFVVIVAYCYPILLLTEKAFKNLLQYDGDPDSAALKKA
VQSHQLLVKLNKDLKAFLGLPCAFETLFVSIILTLTAFTSVTSTDALVVGAYSSGFILHF
LAALLYFSLGQLLENKSEELFTILYDLRWYTFSPAVRKDLNMMIRQARKPFVIDFHGNYK
FNLENFMQILSTSYSYFTIIQTLTDNH
>AlucOR37
MTPKVGKSEYVIKDTKVFATCRIPLVSMGFVDDGTVMSKIRKLITVFLMYSAPVHHILPA
FVDETINLDGVLIGVSLLMLYILICVSWPVMFRRSNDIIKLWKTVQKGFYQYSDPLTKEE
RTLLSETDDLVIKTTRISIIAYFCAGFGTFLKEITPSSLRAYKTPYPGWFPWTINSNFRF
SMALLYQLALCLNTTFALEAIFVLFAYHVIHFECQLRLLSQHFKDTFPTGLSATVTYGAG
YRKNTLRRLNECVRHHLVIKRFHEQILSYFGICLLVYRVIVTIMLCVLCYLVTTGISANK
FLQLLGLALALLFLCFIFCLKGEKVTLMSDQWRQTIYEVDWWNHPVGVQRTVLMMQLGAT
KPLRIYGVWKPAMYSHEGISVIGQETFSFFNMLRAMK
>AlucOR39
MGYRVYPQQDLSDPSHMFSFQLNALKSTTMWKPDNQKYYIPFMILFAVNVFVLAICTVGL
LLKGCSTKDLVDRSEAMDIFTLTGSALYKMVFFLYHYEQLVDMVTCGLALVRNLPEGWTK
NCGLLSRIHYTAGFLVLLIWGLAPILKVMYGETTWAEMKLPINTYDPFDSTGFLFFLFYI
TGQYVLVLSAVIYMAADCYLFTSIYVAVGALQYIVDQFENMRDLNNNNKHTVADTMHDCL
QECIEIHVHVLDYLRKTDKLFKSMILADVVHAVISLSFAMLQTSESKGIFEGVKMVLFVQ
VCFVHQFLNSHFGQELIDKQDNLAKQIITDIPWTDASRKFKKSYYIMLTCVREPFKLSAW
NVYFLQYATFLEFSKTMIQYYMVLQEVQDEAEVS
>AlucOR40

MIPFVFKRDDSVDHEVVKGYQSTYNYIMRFCGLYPDFRGFWYYISGAHLNTVHLAYIWFL
AAYMISTYYAFAYRDMELLSYELCYGLVTLIWFTVTHYTIYKRDQLDSLFRKVGRGFFTY
EKPIDSEEEAIIDECNTNCRKTFQKTLALTTILAFWTCIIPPLPKAVMGDYSSIVEGGVP
VNKHLALPTWNPYPTDTHLTYWTMWMYQALAGCTEAYIIGATCILYCNFCTIINRELKLL
RFSLGNIKNRAIHAFKMRGYSLQLGQKYENSQLYQVCLVHCIDESIKHHIELKQFHGAIQ
NLLGFPIFAIFSGSALTISSPMFMFLQMIGEHEESSFTLVMNIFQYTIIIFGFTYFLANY
CLFGQSITDESALLHFAFYDTPWPEAGLNFRRKVLMGMIHSRKPFVLTAHGLASASSETL
VDMLKTVYSYFNLLAAT
>AlucOR41
MGCLQAKIEEWSDAEDEEMMNSIRRRFGLFCQLSLAYPSWKPGMRRWTLLLFFIHTVLLT
THSVLLGISGVLMVLEWNMELASLTIHFSVILFFAIFIVYWMNSQRPLYTRQNMLMVTDV
GSYKSGRIYDDDFCVEERRKNKRELLYYITCPVFISLTAGYVLTVPYIQHWLYSSGESPY
TANMVNKHLPLPCWYPFPTHEGVLHLMVLLLQLAAALCGAIVLVAILLLLIFNTQRIRYE
MRVVGYSLTSIFHRAKKMFLEQNPHRKGDNLRDDPGYQKVIGICLKDTIIHHHAVSEILS
LFTKQADMPAALAYTIGTGVIAMCLFNILMALRDENYTSVVLFSMLVFVETLVMFVMSLC
GESITSESVNLRHELYFTKWYNLDIENRKTLLNIQTNLVEPVIVSALGLIELNMN
>AlucOR42
MRMTENGKNSDPDSLEIAEEKATKYLYQRFFVLPVVGGVFGFHQTRWWSIFTKTVFTAYY
VSVLSTIVTLSYSSYLNRDNMAVASGCIHILITAFVVLGISLTLQRLRKDVVQLLSLDEI
ICEYQCSEYLSNLIRKSDKKLRFLLIFWVALYGSSAWIAVIFPFIDVYLGYELSLSNVTD
VYWKGLPFASWWPMDADNSNLAWTTCFMSQGLYAFFAASSATSGMLCFAIFSEDIFNHIK
LLVNSIERLEKRAKLMFKMLHPGKSLRNSLDEYDECYYNCILQNVKHHQKIVIKKDLLMK
IANIPVAVPFFGGAMLLGLAGINLLSDGDIRIAPKVLFTCLGVTEAAQMFLLCHYGEQFR
TQSELLFNATFYTKCYRRSMKCKRAMMIFRLGVSRPMAISAAKLIVLNMGTFANLVNSAY
SIFNLQSITTQED
>AlucOR43
MRGTMELDDEEVMQTLQESGLRTWIGVIAGFRFAQQPRFKGTLKGRIYWWYEILTDLSVA
INGISQFAALLTPEFRMIDRCLMCFPAASCLLCLFMSNYPRFKRKNFRSLVEEYENSFSD
SQYRHHLEEQIRKGAKHTRSVIMCLVLLEFISMFIFCLILPVLNEATGFAFGPRRLAVPS
LWLWDPLAGFWNYMAVVFVQLCGSVFVSLKKIGFLESFFVYASRQICMFTHLRYNLGKIT
DPLIVNDDGKVDVKEFTGSNRYLMKRKLIGWVKNHQNCLRLFEDLVKLYEWPLLVYFGAT
ILILCTATYVTSDNSIDAQTCVICGVFNLGIFFELLFICRTGDRIKHESEKLLGALNGKN
TFLLKSDEYKYLKMILTRCQSESVINASGGFPLTITTFIAIIKSSYSYYTLLKKVNGQTD

>AlucOR44
MKSKVFYFTDDAPKSTWDKSTRIYHRLRPVLLVVLLIENAIGLYFLEGFGVMDGSIIYLP
ICLLLFVINSTAYFSRKAQDRLIATLNKHFTESNEPWMLTVSHKYTTKVWKYIKLILIYD
KACKCMYLAAPLICDTVLHYVFDMLEKPFYLPTFITPWLPKDVTWGPHYYLILVFGWWGM
LECLNGIMGFMIGYTLLVTYVLIQVVIFKEKVKSAKIDGSQEEVDAEFRTLLSWHVDNLQ
LNRDLKAYFGFTCAFQSIFLSVGLTLTVFTAVSSKVIAVKIAYGVGFFFYFGTGLLYCSL
GQLLENEVQSKHSEKFEYDYATVSHTAHTRLPRSMQNELRKFHADFETVLFIFYLTTISN
IVGLKFFLPSSPTFITVGTKVVGTKVVGTKLAS
>AlucOR45
MLIHSDIDKYIKFMKGYCVWYGKSVTWDDTRFDLCRKYYTESKCYLSAVLLCVVSFALYS

TDDFGLQDGTFIYWPICLMMLVLTSIATATRHQQDVLTMSLNDNFLENTESWMRAIKDQN
INRLWKMLRFYTVYNNTISALYMLVPLVVDSILHYGFDYLQTPFTLALPLTPLLKYSNTW
NAQYYVLTAFNFWSCAEMVFMLEWFLGNYLLLTTFFLTELIILKHQVKSLDFGKNEEWDQ
QVESIVNKHSKIMKLNVELRDYLGLPGAFISFFSSILLTFTAFVSFTSTSIPLRVSYGSG
FVLYFGAALLLTTIGQKLENESDELFKAFYSLRWYQYSPNARKSLNMMMRQARTPLIIDF
HGRYKMNLANFMQILRSSYSYFTLLREVAKE
>AlucOR47
MSMSVWALTIGGLRSSLGIEMTYACLTIMTTVQHFHMYRNRTRTEAIIATFQEIRNTYQK
GTDIEFKNYTRFMWKVVKVYIVMIVGICSAMSLPFFADLVVWFVWETPTAFRIPIGMDSM
VDKEPVRDATYFAVVLFSNCWTILGGVTQMGVDTFLFVSCYSLSSMVKTFCKQLKVPPNS
TPEETTVHIRLLAAHQQALYKLQTEIRRTFGFPFFVQNLLGSFCICSLLYVMSENGATLL
SQFIYVFNMIAVLMILASTAHVAQHVKNTTSEVFEALYEMNWYTLRPSDRKYLVTMLGVA
RNPLCIHFYGLLPLDMENFMSMLNTSYTYFMFLKSIG
>AlucOR48
MSESVAVFKSLNLALKAVGFVDSASGIKSYLWTTWNFIIIFIGVEFIAITIPLNIIQDND
IYLKLECVLCIYLNLSMMFRFTVLTIRKKRLFSLINRIESLLQQQMEREFDKAFITALAR
RCGNWAYLFTFAMTATCIEPVVMAYVKYYFRGVEDPVPFEVNLPFEKKNNIHAVVWYEVF
QFLGCSMIVISTNILFSTLSETTSELVKKIAENFEKIDETNADYLLKQTIKWHTEVIGIT
KETNDILGITIFADAFFALQYISIAGFLLIRVGLSNTTSFSKYFITYICVLTNPIYYCYS
GHRVSLMGDVLYDSIYNNKWYRLAPKTSKNLILPLMVARRGLSWNYKALNFDMALYLEIV
KQSYSLITFLKMMK
>AlucOR49
MIRNWQEEEEGTTLLERMGQKFLNGHSIYLGGWVLRFPVRLPPFFYLTCAFGLIVKMILN
YDNLTLIIDCAHMMIHMIVGLQTTLLALKQKGNIMRLKTQLDNFGYVKDLETAGEKIKEE
CEQEAMELYKPFSRCILITINVYILFPICKLFTEAGRAKLSRVLVWQMWMWVPDDTWWGF
TIIFLFELVTSLFLLLSVMYTVPYLACLGKMTVGHCKLLALQLESISKKATQAAFTSGSF
KAALNHEIDECARRLHETHMLANEVAEVYKYYLSSFYYGGMFALCMSGLQAVSATENIEE
SLKFMGVLTGELIAIGLATYVSEGMIQAFADVRSSI
>AlucOR50
MGEKPYTMDKNGEMEIDWLTQEDKNHINFFDQFHCWSGMWRSKKSIQWTYFWLSQMIAFS
LVYFYSFYFLLSELELLAHLIHHMMVAADDIMYIYLLNIHRIRVETIHDYASKTYNYDSG
IVREKHKKLMTEQLKLYPKMSKFIFLTTVATAMSLEVNYLLEATYLKSYVTMYPMYLPID
LNHPVTYTIVVFLQHLQVFISMILCGGLMSILFVVWSHLKVELDTLTFAITHVDELVEEK
LRHFSYTNPADKEKAKAEFYNGFCYHFARHHAAIKRYFGAFQISCKVTMTFVLISGLICF
ACVGITSVTENIGIKLKFFVVMVIQTLIIYSWSWVGQDISDKNAALQNIIGGTHWWKMPK
TCHSTLKLMLVGTSRPMLLYTLIGQPNNIDSFMDMTASSYKIFNMVYQVKFSS
>AlucOR51
MSVQGEVIASKAFKDNFGRYMVWSGMYSESKIYSGAIHVFFLVHTVFLAYTVILSLDDEK
LMGESAHFTAFRVSAFMLMINAVLNKDNLEILFIKLGQNQVHDYQNTLSDQCKKEIQAVR
KNCDERKDFYGTNFLRTVSAALVIFWVRSLMEYYRGHMDNPKSDNGVNKNLPVPTYLPYE
SHDWPGYQFALLSEVALVMMSYFLVLGHDCSFICFSEEVLRELEIIIITLREVEQRSDHL
RKEMLYKISQKESVHICLKHSVIHHQKLIKIFGSFKRYCFYSLFFMLSGGAFLICLSSLM
FTSEKISHQDKSVFMMFLANELFHIFIFCYYGEHIMDRSIEVGNSLYNSSWIRIAQYVKP
AFIMVKLRCQVPLSLSAGGFITAGFDTYGNVLRTAYSYLNLLQATN

>AlucOR52
MGFSSWIAQQVVTEDEFQIRLKKYGWLHYLFQFSLVNSCYRSMTSLLMYYISLMFSLSIV
IFHLFCYIKTALNAYSFGRADMSVANVHSVVLGIFIISVLTSYAIDKTKTSAIEQLYLES
FLSYENEYPAPVTLFQKLMTLAGKMGIASGGMGLFFNVYYVAAPMIDFRFWKESCVVKGIN
FCLALPHYYPYNSEDGWSFYLTELFQLMFGVYRISVFCAVQVTLTVWPLHLIQELTRLKT
SIEHLEERIKKRYHRITMRNVEKVNLITMCMDKTFNECAKFCINENISHHHNILKYHERI
DGIMALPSFLAYTTGTLTMGIAMVKLLSVEGDTTLGGNLAYVTVLAAEIGFMLLISTMGE
AVTIQAEEIFNEVSHIPIENYDLEFRRNVIIFMEGTIKPIALSSSKFNKCNMEAFGNVLN
AAYSFYNVTSASAKLEK
>AlucOR53
MSKFNEYWDNWWFGDPPMNDKVYDAIYEEFNIILYVSGLFPDPRPIRRLITLGILIFNVV
NCAAYVYFLGVTATIQINDFVTASQTVHFASVDLVAVTCMISIVASRRHMIDMFRTIANK
YFDYGDDFEIPEMVEEYRTMKRQKIIILVVLPSYLALNAFVCMMGRTIDGYFGRASNETY
ENGVYMLTPEPMWYPFTIHNELMHWMIVMSQATGAFALASAVSGSAAIMVLLCQSINLQF
KIIIYRIRKAEECAYHLHRKNGGEKLKKSELYSDPSFMGYFNSNLNKLAEHHSILIRQFD
SLYQVVKWPAGCALLLGSLLIAMSLLALLSGDGKPSILLLAALLIVAEVMNMALLCGQSE
SVQELGQSLHEELYNMNWIELNPAAKKTMMMMILQSKRPLVLMAGGLQPLNWEAFSGIMN
TAYSYVNLLLAADV
>AlucOR54
MFEPSEEILRELKISRETYLENDFFLARLSGLCRWNRLFSLFYFLSMTYAVASAVGFVLA
AFSEKDRDQVLENIHFVPLIFNMASQAASYHYTQKEYLQLFRAVDSGFFNYDGDLDIATE
LEITEVKSVAKNRKKKFGHFYSMLMLVAGLGQILKKPLLYVLRGGGTKPVDGENNLVWEA
PYGMYVPYADHWISYLTGMFLGYSACTFVSITAVGSVLSYQYMSEELLAEFKVVEITFSK
CLRRAQTMYENRKNVLKANGKTSQITMKDCIIHCMNLSVKHHQHTLRMMNVFKDLMFFPL
FMVIFDGALVLCISAYLTISDDVSLNLRITMPSVITAEATLAFIFCYYGEKLTEATEDVG
DSIYNSDGWVQHSDIIRPYALIVKSFCNIPNELSAAGFSSVNHNTFGNVRT
>AlucOR55
MLHEWTLFQKTGSSNSSDKEHYDIKETKHFATCRKVLVMMGFVNDGSVIYKLRQPVFVFL
LYSAPVHHLVPAFIDKTAPSDQILMSWSISMLYVLLCIAWPFMIIRSSEIFQLWATVRKG
FYHYSDPFTAAERTILSRTNDIVIKSTRVSIIAYFCAGFGTFLKEMQPESLRQYKAPYPG
WFPWTINSNFRFALALLYQCAICLNTTFALEAVFVLFAYHTIHFEGQLRLLTRHFGDTFP
PGLPATVTYSAEYKKRTLRRLYECVSHHLIITGFHKQILSYFGICLLVYRVIVTIMLCIL
CYLTTTGISLDKFVQLLCFALALLYLCFIFCLKGEKVTQLSDGWRLTVYEVDWWNHPVEV
QKTILMMQMGASKALKVYGVWKPAMYSHEGISVIGQETFSFFNMLRAM
>AlucOR56
MSRYGKIEDDELVNSIDIWYLKRSGLWEVFNHYREHGVRNRRFTLWKIITLILFVPIGFF
SLCGPFFTETDLEGMTLVILNPMTSSQTVIKFAILWYGIETQCRVLELFKRDFLTCVPPS
MQAKASEILTKAAKKANKLANLGILTDVITVSFWNILPLLRSEYFRIELGITAFGTPLRH
NKILGFWYPVDYDETPYVQFVYCYEFLSCVWAGFVIALLEGLVIHLVILLTANIKVMHHL
LEELKTSNGTLNSETLLTYIKDHQKLVKISNDMRNLYNMMITMELSTGLIILIITIFNFF
LSSGNGDLVIMFKFMVYLMYTLVEVTVYCYIGSDLETTSEDLGFAAYSSQWYKVGKKFRK
TLQMLMVRTRYSLALKFGRMYPINLMALTNILQTAYSTSMLLYRATSQDEQKEEAQILM
>AlucOR57
MTPEKFMGTFSVQHAILFSALFIFHTMYAIYEVTITAFFARSLLETVSHFYIATYLFAFN

FQWYFMLYSIRTFHENEIFLEDFKCTQTHADFATRELDENVYIFTRVLFVSCLCWTVNSS
THIIGPAIEALISLIKTGQIDNVLFILPPVFSTPWWAQIIIYFCNAITMFGLLLYCLASY
TIMGFKVLKLKTLCDILNEALRNDVEEESNIKAYIKDHQIIIKAAKLLNAQLRTLNGFMF
TACYLEFAVQLFALTLIDPSGSYYFALALDLSSIFLILVFQCWMGTMITHSLETVANGVY
ESLWYSRGNNNRSEVILMTQMAQKPFVQTIWLGTLKVERATSLSLVRSSYAMYTLLNFFQ
DK
>AlucOR58
MSFLLKFIDELAEEEDDELIEVLKNEYGHFLFLAMIFPRWKKPLASFGLVLFYLSTIVLH
HAMLSYAVYLSLLEHNWEQVSFLTHLVILLSFAIFQPVNFNWVRRVVAHVHRTLAKDVGI
YCSGRIYDDPVCIAFREKIRLEKKFYMTMSAFCLMMGGILWSGLYISKSFSDIEQSYSSS
GLSLKLPLALYYPFPTDRGVLHYVILGSQLLVCLVIGFLYLICEVLLINLFLKIKYELQV
IGYAIDSLVSRSINAVGVNENNPQKDVLVIEDTKLQRSVEKCLKETIVHYQKILGLLLIA
RANLDAPLAIVMMLGLLVIGISLLNMLAALKANYIGMFLTFGMLVCAEIQAQLLVCLLGS
SITEQADILREKLYSIEWYCFDMKNRRILLNFQACFTKSFVVTAGGIAEINMVTFSWILR
AAYRFFNLMRSTS
>AlucOR59
MFVFIREKELKRDPNAMIGRRLVQARFAIFAGIYPDFYGWRHYYFVIFLWIIHPGLYSYF
VMVYVLSFVEGLRYMDVELLGQVLCLGTITVIYCLVSIYYIAKKTDVDELMMMAGKGLSN
YSRPTTQEEKTILDSKEKSTYKYAIGSSIMFVSVSLLHMGFLPIRRGLKGQYTSITNDTA
PINKYTPLPVWTPYVCDDLTTFLISYFTQLIPGCMEISIINACCILYIGLCQQLTGNLEI
LVNSLRQLPERGLHMFEAERGIVEKFTPELYQNEYFLRCLNTCIGENIEHQYNIIKFYKK
IQSVVGFSILAIFSGTGLIISTAAYSMLLIAERERDTEIIITNSFVWSFNLFVYTVLLTL
YCYYGQKVTDKNEEVLEALYDTPWIEADMAFRKSVIIAMSYSQLDMTLSAMGLISASLAT
LLDIIKTSFSYLNMLLAAR
>AlucOR60
MNDIGGLALAKSGLNNMMSILGGFRGPREVRFKGTIYEHIFIAYSYFGLLVSHYHVICCY
LTPIFMPDMSFKDAMFFAVPCITTTFSHLRIYYMAWNRSKFIQLLEMNEEASKDDYYEDE
LQKEIDGWAKQVRILQPILYFAVSAPIVPWGVTPIVNEVLGNPWGPRKAPIISWYPYNVQ
ETHFWVFTIFIQTMAGCHATLSNVMFDAVFICISTRQLALLIHLKNSFSKIFQVIHVDPK
GISWYTNYRAEAVEKEEIENDLTQRLKYGIRKHQTTLRLSKTIVFFLATRFWIICLIYEL
HMYLFFMEVVQVRKS
>AlucOR61
MDTFCGGFMGKVYYLKKHNIYINVYLSCRVNRDNFSSFYRDFRYFLSQFYHLMAALSAAL
LGGSVCIVFIAIAEHLLAQLEILCISFRNAIGFIPAPGDRVGEKLAYQRVKSCLQHHNII
LKFFDEFQKYYSIPLFCMLAGTTVAMCTIAFVVTDPSSTFGVSAAFLSLMAPEVAFCICY
CTYGQKITDMSDILRDTVYNAPWYYQPKPVKMALLMALNKTRTPMTLSAAGLKDCSIKSI
GEITQTTYTYTYFNALQLFRGKPAYHRE
>AlucOR62
MGWRKYLTEMREFEDEDVRKAIRDNYSILPRLNNTFSYIDEGWVPLAIIHSIAFIIFVDL
YLYLFFVTCYLLKDDFVLVGVQFHYLLLALFGLVFQFHLYNSRKEVCVLHKIMAQEFFAY
ENNEILAEEKTKLKKHMIKQRLQLIPFMILIGMIGLFIVGVGPLIDNMVGAGHDSDYLNG
VYMKTPIPMYFPFEIVDFTTHYVATGFQIITVAMLALTISGVVS
>AlucOR63
MAEDDYTLTDMAGVYLLPHSRAYMFWTGHWVGAVPGPTPVPIMVARALGGTLTYVAVFVV

CWGSLNGIMHGSGTNDMPMNLIIISCSISSVHKYCVYTNQEQGLGRLGRWMKRVSNREKK
NKIPKTITDHILKVCLLIFYYSGTLAAFMLLTKLALTGTTYNVLVPGLENVLLLKLAIAL
SFMSLGFEVVVDALILMNSLFTFRRELMRSFEEWRKLNFDSENPNQYKEELKERVQKHIE
LLTIFQDLREFNNSMFGYQVFAIVFTTCALLYGMAKETENSHKVFVQTMPTATASFLEFF
ILCWCGEDIKFGFEQIHRSIYDTNWYEASLEDKKSMTIVLEFSKNPIILTGFTVFKANLE
TFVESMRQSFSLYTILSEMV
>AlucOR64
MSGFHGFWDKWWYGDPPMDDKIFDAIHQEFNQILYFCGLFPDPRPIRRLLTFGILILNIV
ICVTYMYLLGATAVLQNEDFFTASQTAHFASCDLLSLICIISLLLNRNRMIEMFRASAHN
YYDYDDDAKISEMREEYMTTKKQKTIILIGLPSYLSLIGMVCLMSRTIDEYLGGGSNETN
IDGVYQLTPVPMWYPFSIQSEPMHWIAVSSQAIAMFSTATSIGGTAAILVIFSQSISLQF
KIIIYRIRKAEKHAYQLYRMNGGKKLKNVQLYSDPSFLGFYNSNLNKLAEHHSILIRQFD
SLYQVVKWPAGCALLL
>AlucOR65
MSFIVGYFEKILFHFDKLEEPRSEEITKLHRKHFSILLLVSSVDLNLGRKYLFWTLLHAV
FNYVVLAAQTCVLMYSTFLLRNDFEIGSGVLNYGLLMIVAIGILMNMQYFRHEVLHISGI
MCTGLFRYSDKTMETEDMIKFRKHMKFQRQLLIALAVYVATIGGIVVLGPIIDEKLGMGF
DGTFDENGVNRRLPVPLNYPGIDTSKIFGFLLALGMIFQSGVETTLIYGGATLLFATACQ
FILTEMKTLSVSIQTIPHRAAKKYCRIHNVSKKSLDLKTIFDDSEFQDCITDCLKENIQH
YLEIYKFTKVLETYVKVPLLLAVLVITLAIGLTMMKLNEDIVRIGATISFTSVALGELCI
MFLIAVYGEYYLTMSQEVNWEIYFTPWYKFSVKNQKLIRQFLISTRNELCIFAWIVRMDM
EMFASVMNSAYSFFNFLNISKTLEEDELN
>AlucOR67
MPVSQSLWNMIEGKMEITEEFEQKVTETPVERTAEDVFSTQIKALQIVAMWPNFKQNSNM
ELFTRALLKINTFVLAYCTLALFVKGLLTQDLVDRSEAMDIFTLTTSALYKMIFFYTHHK
EMDDMVNWGAALAHQVPPKWMQYTTFFSCFHNFMGIFSITFWGLCPIFKWIFGETDLDGM
TLPINVYDPIGVTGAMYSVFYIVCDYGLLSAVQIYMASDAYLFTAIHLAIGGFETLNNKL
RKMGQINFNKGPTVNDSMNEYLKDCVKLHTHILIYIRKIDRLFRSMIMADVLHAIISLSF
AMLQASESKGIFENMKMAMFVSYCIVHQYLNNYFGQNLIDQQEILNKELLISVPWNDGSK
EMKKSYQIMMAGCLKSVRLSAWSVYTLQYATFLEFVKSMISYSMVLRQVQDQTVKQP
>AlucOR68
MSDRHPDIAKYIKLMQATRNWYFAEDSTSHPAVDLLKRCYYHVRPSLFVFALLANGYGLY
YREGFGALDGNLALFPQALASLVTSSTIYFNRRHHRKLTMLLNQRFLDKNEPWMVEIKNK
YVSAVWKFIKAVILYQEFVKIFYVLAPVIVDSILHHIFDYLETPFFFPLTFSTFLTDDDK
WTGRYYAVMFLNIWSGFEIVANLQGFIICYTVMTVFSVVELVILTEQIKSLDFYRSNGEI
NEQIRMVVKSHNDNIALNRELKAFLGPACAFLSLFTSLVLTLIVFTTTVTNDLMVILAYA
VGAYFYFVAGLLYCSLGQLLDNQSSEVFDELCNLPWYRSSPDVRKSLNMMIRQAHNSLII
DYHGHYMMNLANFMNIMKSAYSYFTILQSVTGSD
>AlucOR69
MGSVTAKMDKWEKDEQEEVMKLFKEKYGPLIQLALIFPSWKSSSRSSTIMIFVLHSVVLL
FHWMMIMISIKRSLESEWNFEMLTIWIHFAFIVFFVFIVIFCVNNQRSTYFRQYQIMSND
IGHHRGADGSIYETDGCVSEAKNIKREMLLYMIIPVLIFLFASTIYGLPYISKWLEGMEN
PYTLAMVNMNLPVPAWYPFPTHAGLGHFTAMAGQALVALSVGVVLITIILLFLTNALRIK
FEFRVICYALQTLFTRSTTLFLQMQHDMKDIGNSEHSYQRVIGSCLVDIVVHHRAVSELI

SIFEKQVFFTCALGYMVGTLGVGLSLVNILEAMKVGNYVSVLIFSMMASMETLLMFTISQ
IGETITEESVKLRHQVYDIEWHKLDTQNRKILLIFQTAITEPIIVKAGGVINMCLDTFSN
IMNLSYSFFNLMTNTN
>AlucOR70
MARTDLSDVIQLLQFTGHYFSFKGSRREKTYETLQKLRVVFMVICNPFTLSSLFIGGLKK
SMGVELFFGLMGFLTAMQHVYAYRHRKTTEDIIRSILEIRRKYQQGSDIEFQQNTRAIWK
VVYIYFSAMTSLLVFYITIPKFVDILYGILWDDPVALRLPQSMDAYLDEHQHRNLKYATV
ALVSSSWSFVSTYSHFGLDTLLSLVGFYYSSLVKTFCNRLKLNTHLTSKELEGHIKILAA
HHHELFKLSLKMRSIFGCPYAMQNNFGAFCIVSLVYALLSDDSSGLLIKVANLFNLMILA
GMLTSTSYIGQHVTNEISAIFDALYDLPWYELSPSNRKYLVTMICVARDPFTIHFHGRAP
LNLANFMAILNTSYSYFMFMRSTL
>AlucOR71
MAPQVNLFKAWMIWMKIAGADPPSVNFPYALALLWKLIMLYGSVHYVIIMFLAIVVGDSA
FHLKLEAGLFLLAGIPCSYKHFVFVIRKNKLHEVIDRLNTLLEEVEDVYGTETLAGWQRI
CNMVMYFYSTQFTMLVVPVFSFFYYIYYWEGVEATPYEVYIPFEKENHIHRVMLYELLSF
LGPAAGLITGNIFFGSLTVAVSGVLRKIQEQFSQVSPSNAQFLLHRTIRWHSEIISIVGE
TNRLLGTVFVVEYLLAMVYICFSGYMLLKVGSATEDVNLNKNIILCIVCIVMPLFYCLCG
HVIVLEYDKMSDSIFQNDWVSLQPVDRKKLILPALLAKRGLSLHYKKLLKFDMTTYLKIV
KQSYSFLTMLKLMENT
>AlucOR72
MSVNWNRKLNSLNHTRIAFDVGKYKDGRIYSHKACIRMEKELQKETILYLCIPLLIIILG
GAILIVPYGSKLVRGYGMMYTACGVDLFLPIPLYHPFPTHEGIHHFLALISQVLLVFCLM
NGIIAGVLNFLQYSQRVKLEYRVLSYSLDTLFARSKRVYLRHYPDKKANFTIRDPEFQHI
LGSLLRDSIIHHQTLVDMMNNYHGLITYPVAVGYMTGAGGIGLGLLSILRALQKR
>AlucOR73
MNTEVTERFEYASKAYWGALGFTGLDAFLYEKPPKHNVLRWYAFRIIYIYFVFVHYPIFI
TMQFWGIVTAESHTLMQISFDISLMGYNIQNIIKLLIWMIRIKTVRSLRLNFSKFNVNKY
RPKLSSWIIKRDAENALKFTGRCYWISYANLLFWVVLPTATAIINYSTYLAGYADWQEND
FPRYSNTRFPFDLSQHRSQVIVSFLEVILFTLGFMSFQSMDMFFSVIIRMAQAQFSVLNS
ALFALDGELDKFWGTEVPVNDCKPPMKIHLIVQDHQRMIRYGVRLRKFLSPILGLETLNC
ITIICNMTIVASSQVSGGGEFLEVALAAFASSLVVITCLVVFFTFTSMTGQLKDAEESVF
YAMYSSKWYERDVSHRKSIILMQKQAMTSRRIKMFGLGDMGRSTFIDGLRMVYTYYNFMQ
RFK
>AlucOR74
MVEDLTVKDLTGIYLLPHSVAYMHFTGHWIGAVPGPTPFRVKMYRAFGGTFTWLIVIATA
IASLNGMLHGSGMSDISMNLIIISTSISSLHKYSVFIHQEQGLGRLGRWMKRANEQNKIS
KNPDTTTDRILKKSLVSFYYSGIVAASLLLVKIVVTGYTYNALIPGLDQRYQPLILIFME
AFSFSSLSLEVIMDALILMNSLFVFRRELMKNVDEWRKMNYKSDNPQQFRQQLKTNVQNH
VELLTIFQDVKNYCNSMFGYQVFAIVF
>AlucOR75
MDILHYSDQTGFKFIPVYDHMLRSIGVHSERGETQATAIKRYFGNFLILVAIVQGWSSAV
AAYDSLKEEDFRAVTNVMSYLSVQLSSFSKFHIARTHMEVTHRLGNWIVEAKKNRPRDMK
QPQLEFLVLKVNPAFFYFGLFATFFWCSVPLTNNLQAFIPTQYPFLDKQTSNSISFPLIQ
VPLYIFFTVTITYTATSLLHFLAIFTTEVKLLSKKWEQVFYDKRRPDNYMELMKDCIQQH

IKLLDVMKDLNIIHDSMFAFQVFIFIVHFVSFNFCLVMTSGSNALSSVGPLTMSSLMEFG
LLCWMGEEITDALQQFHRSLYMTNWYEASLEDKKNMIVVLEVLKKRHALTGTKVFVASLE
TFVEAARQAFSAYTLMKGLTTTE
>AlucOR78
MSQRPDPEGPPSKRTYLSKGLKLKSSVRLDAWRHIGYRLLLLGMTPENFMEKFSVRHALV
YLIFFLTHSLYSAHELTVALFFSRSLLERVTHGYILTYIFTFNLQWYYMLRHVGNFHRHE
MSLENFASTQAHLDFAEEVFNKNINAFLKYLFISMLWWATNTTTHVFGPLVETVLMYVRS
GDFKLATVLPQVFSLPMWAQVIMYIHNATATILLFVYCLASYLVLGTRVLKVKTQCDILN
EALRRDYDEESNIRSYIKDHINIIKSAKLLNGHMQMLNGIIFTACYLEFATQMFALTLFE
PSGAYFFALAFDLMSIMLILVMQCWFASIVTISLQSVTDAVYETNWYRRDKDNSLNVLMM
LQMAQQDYIQRIWFKSFKIERAAILNLIRSSYAVYTALLIFQE
>AlucOR79
MTVVQTNKRTAMTDVHHYHSLLLTMLEIAAVFKKREGSIFSPTGFKMFRVANLIVCFLFV
TSCARYVFHEKGAQFFTVAIGTGSIEFCIINMILVSKSDIIDRMLATSAKIFYQLPQNEE
TRDVLETYRTKGYTFMRAFGMLIGVNEVLGLIKPFWMARLTGQLGLPFDISCLGIPTVPC
WIFQIICTSHLIVTVAFHVIIVKTLMYLTWGHSIVITKIMNRRPVHDDEENDRKIIELYC
DFSRFSTSFSSLFGLTTFIEVTFTSTRCCFLIYHAIKSLSNNDMEQAIVSVTALIASIAI
SYVMCSCGEDLVEINQMMRDGFYNSKWYESSPQSRRRMLPMLVLSRVPIRFQYRYYMYFN
YEILMKIMHSTYSLSAALIQFL
>AlucOR80
MVVEVNSRTYYGIEVVYFKFIGFWQFLTNGLGKDKLVISSIVYGLMFTFFIIVQILDMFI
KDYDFSIFSEKLSVNLTCFESVIKIGYYCFKRSSLLELLPLYRLDLLLSAKHSPVISTEI
LMANRRFVNGATKSFVVMIFSTVGIWNCLPLLKCFTSGGCSTLQIMPTWYPGDVSYVPLN
LFVYIFEFFIMIYCAALLYNVNCFFSSLALTASAQFELLSNNFANIESNAERRIEDHASS
TDEDTKKATMYVLLRECLIDHQTLLGILQKMEDVFNPMFLFQMLTSTFTICLVLFQLNFH
TASGDDLPIAMACKFVMYLVFGSMELLVYSWGGQIIYNKSEEIYWSLQKCGWEVGCDKFK
TNVQIALQRSQFPVTLTAGKFYVVNLASFSQVIKASYSYFTFLHGSISNEE
>AlucOR81
MGSFYQGITSPSENALKKLQISADTYVENGFFIARFSGMYRWSLLYSISYFSCMTFGIVA
AVGYILNVSTTDEWDKFLENIHITLLIVNMEAQGVAYHYDQNGYIEIWRAIDKGFFDYEG
TLDEETDEEIAIMKSELRNFKKVFQHNYTMLMCITTVLQFSKKPITRYLIGGGTVDGKNN
LIWEAPFGLYFPFADYWIPYLLGLFLGNACGLLILITALGSVLQYIYMSEALIQEFAVVK
KTMSKCIERAEQIYRNRSNSNQVEHQQWTMDDCIIHCINQSVKHHQITLRMMNVFKKLMY
FSLFAIIFDGGLILCISSYILINDEVGITFRLPMPCVIAVEASLALVFCYYGGKLTDANT
DVGNGIYECKRWMDHSKILCPYALIVKSYCNVPNELSAAGFTNVDVRTWGNLLSTAYSYI
GFLLST
>AlucOR83
MMGEWWKDLKLPAGRHPESAPTLKKIYDDYIRRFDHLKMFKPIFCDTQFKWHTLLAYFGL
FLHNTFIGFSYLVTCILNMDDISQASFPANLVIIHLIVLAILYQLVRLSWTENVTIIDNL
VTIVKGREEYCKYTGTQYDVYLINRRSKILRRVTQVFTYVFGFQISWLILPVINSVFGEK
RVPNEVSNGVAVTLGVPIWTPLDADHSWFYYCIVCSMQLFFFGSSGLFIGLGVFYNMMSQ
MSILDEMKLLIRSVGELDSRTSRLLTDKYPKLSIDKYSEKYDKCYFQCLRDNIIHHSFIQ
RTFSEYQSLVSVTLAIPFFFSSIIIALFFADITSGSLTLVELSIEVIHMCMNIFLMAMMC
YFGQLITLKNEE

>AlucOR84
MYYSPWMANNCIMDTQEYKTARKKDYWYLFEMSGMILDWRPGFYIINLAYVVVMVINAVY
TCACLIASIFKVDDLIDVCQYLNFVGLLFVSLSVLASLNFQRERTLETCAIGSTEFFDYG
VSFSRSEEIEQYRKEGRRRMKILFMVIPPWLTIIALSLMMSGPIDAAFSYPKINATYVNG
IYQMAPLKMYYLHPIDNEFIRWLTVLSQAACSGNTALVIGCADLIMFNAGQNVIIQLEIL
NLAVLDTDKRASKLYELKFGREPPSDPVDKTNDRPLMNIYGFCLKQIVDHHKIILRRAEV
YHKIVNWPCGIVVVNGSIVVAMSLLSIMQGGGKPSVLVLSFFLIIAEVASIFMVCEIGQS
ITSQCERLFDSMYAFKWMDCSVEVNRAINIMKCRFRKPIIMTAGSLTPINRDTFGTMMNT
AYSYINLVAASGKEDAD
>AlucOR85
MSKAKKSAVGTSSYKELEGLERAEALKKGYDENGGAYIRLGGQYVVSRSEIWRPVLFYAD
TALAMFELVTASYFSVLQGDMEAASECFHFIQMIFNMMVISANLQYYRKNIDELFTAIGA
GFFDYGDTIDPQTKEKMDKHMMDMRANKKFRFKVFVLVVWVLGGCMFIKVIVAHFRFGDT
IDGEGGSVSRKHIVAIWLPGIDEWPSYIAMVVAAYLCQVLIMNSIWGFVLPVICFAEELN
AQLHIVGIGLRHTTARARHILYRKYGEQKSGNLKFKYEESLREALKSSVQHHNVILEACK
CASTLLNLPLMTVMFGTAVLLCMSGFVMVEDSVPIIANIISLLFIGGEVIYAYLFCYYGE
MITATSLEIGDELYNDDWWEGRDVFRPYMAMISLRSNRPIKLSAGGFTDVNNAAFSNIIS
TSYSYFNLMFTSKS
>AlucOR86
MWLREWFKNRSSKTASKEKQVVPVNIDGTPDFASFATCLSYQKFVGLYLDGSVINYLKIS
IPILFLTTGCISFAMADILNYKNKNIIWLIENGHWCIVYIAAIFWDTQMGIKSPLLLRMS
RSVKSGVYKYAKYDTINREDLEKTNSQVVTTSRFCVFVYVVAVLATLVKPTILEEYDPYR
HFFNGWFPFEVNSLWRVSIVRVYELGCAWSAASGVCTFFVTFMAYSYHIEAHLKLLIQKI
EKVFDPESSEYDYIPQLDKKIRECLGHHREILRVFNDFSEFCDPTIGCATLMATFMVCTL
LYLMTNPDFDVSVIVTFSGVVAPEFSLLISFRLRGQRITDLSNKINEAIYKLKWLDQDVK
VQKNVLMWLRLTSKPLELKSFGYRNVSNSGIKEVLQTSYTFFNMLKAST
>AlucOR87
MWKCWKKSSAPPPSLKDEAWKWPHESMMNWFGWWAEELERPLVVKLLQVMRAILIPSHLI
FYGSLLYQTSNEFRQGTIISTVKSAFVSGPSTVACFKLYVIVRHRKSLKEITNSMDVMMK
GILSRHIPEDLEKEMRSRWTGCRKLYKCCVYFGCSVTTHASVTPLLQTIAGALLTDDPLP
FDSWPYFLMGYYFWALNTFCIGHVLYMFDATWFAMADNLQIHFAVLKNYLENLDLTKRSD
VDLNLCLKNHMELIRLCRIFRRISRTVIVTTRMCSMLLLCAGTFVLTSAGDEFTSNDRGN
LLSTLIYIAAVFFNYCRCADNIAHQLDELTTDCYSAQWVNAEKSQKTSILNMMTITRMEP
KFCGIASIDLDTFVNVMRGVYSYYNFLTAVDVGDESETSRTEVNEPL
>AlucOR88
MSFLMKFIDSLAENEDDALWDQLHKFYGPILEIAFIFPSWRRSKLPISLAVMSFYAFIFP
VHIWLLTVGIQCVRDDFNLASLEFHYWLIFMFSLVALLMMNGNRNFMISFHRTLTSDVGK
YRAGRIYDEKRPLEWEHNKKKQLLKFLSLPTLVLVLAGFSLLVPYLQKMDGTVEYNERGA
NMKLPIPAWYPFPTHEGILSLLAVLGQFMAAGGLATTVATLDIVVFRLTQSLLFEYQVLR
YALETLMPRAKRLYTLKYPAEDMRKLRTNDEAFQRCIGKCLEDCVIHHQDIIKLIKDYKT
LVKWPGFMAYGFGTGVIGLSLVNMLSAKEQGRYEDIVLFFLLALAEVLNMFMLSTFGESI
TTESKELREQLYFIDWHLLNTSNRKLVLNFQIGVTHPVIIKVGGLVNVSLDTFSSIMNTS
YSFFNLMNAQ
>AlucOR89

MAFGLEQMDCLTKEEIGIKAHFMRLMNLSGTFSRRKQTRLRSAIIWSVIYVPIILTLVAT
CIHFRKNFDLSSYALHHAALITIGFIVNVMTVCMYWKEFHDIMDGSTMSYNYDSGLVKNF
AQQTIHERFKLSGLLVKLVSYGSVGVIIEVQIFFAIEAFYLQTYKTIFPMYVPMDLDDPF
VFTSVVIWQELVVIYTTYLPLMLAVLYYNAWSHLDIEIKILTFAVANIQRIVEEESQNFR
HEGIHRETLEAALYETYSYHFAKHHAHITSYFELFSKCVKLITLLLFTMGPVCLVTVGLS
LLSDNIGIRLKLFWFLVIQLIMTYAICWIGQYIADVSTGISEVLVTAPWWLMPKSCRSTF
LLIMTRCRKPLQMTTDYGVPANMESFMDLLKGVYQIISVVIQMRDG
>AlucOR90
MVASTSKNRKIKNEQAVRGFTRSEEKQIEDHLFSIFNILPIIGGIFGYHQSPKWSALTYT
LNIGMYTSVSLTALNLLYCSYLLRDNLSQVTMAFHCFLISCVVMTASISLTLQRNKLIEF
LLKLQFRGPLAEYHDSDYFQALEGKTRQRIFRMLVIFLSCYGACGLIAVIFPFVDLYLKN
ADQVTNVPEIYWKGLPFAVWWPYDVHNSTSAWILCFLSQGIWALFAPVIVTTAVVLCFYG
AELILNHFKLLIFSVKNLDQRTKAMYERKYKENSRTQLENVYEDCFYECIVQNVKHHHII
LKIVEEFLALANYAIAVPFFGGALLLGLAGMNLLSTDDLRIGPKIFCASVGATEATNMFL
LCVYGEKFQHEGEELFNSIICTRWYKRSMKCRKALMIMQCGSFRPPKITAARMIELNMAT
FSNLVNSAYSIFNLNSVASATEDK
>AlucOR93
MTLKSYIKKTLKWEEPLGLTTMIAVISGAWNTMAPPQSIQRFIYWQSWFQTSTYVLFMMS
AGINIFVSTDFFGECLESLHFLVTAFHVFIKYMTMRFRERDFLELFDDIKRVWSGYRIHN
EKFLSSTLASVNRTTVIISVCIINVMFVNIGAAVLKNILEPDKIHFPIQIWIPSFCRDSF
MYGTIAQVVLFSWPLFIVAQSTTFLNSISVHVEALGLSLAKDIGRQKVWKGDSARRFYKK
HQEVISIVSRVNALMAGNWGFEMLCSSLQLTLPAYRTLRALKRNEIEVFNHAVILSLNFM
VIYIIFGSGNRILSMGEEINDRLYESDWYKLPVKEKKNVLFMLFRATKPVEYRYKMIHFD
LPGFMKVVNTVFSYMALLRFLDGGNDGGNGGLL
>AlucOR94
MSNQDDVGDDVSVRRIPRSAQKRPQITVKNEKEFDGGCTDYRKYIFCRLMYIDDGLVSRG
LTLPLIIIMVHTVTMEVCSFLSIFGSSQIKDGLDCVRSFLLGNLVTMVLFNEFANRRRLG
RLHVFLDKSMRTLRTGLPEEEEILKRARDQASSNLKMYIIIFSNNIAPMVFAQPLGEWFA
GRSWKRLPIPWSFPPSDTDLAFLLIFLFQFIGVIMANFLAMVFMSFSSITIQITALFDVL
LLSLRHIETRAALRSKLEGTDYRQSLYNSLREDITFYEQLVREVGSATPHLRNTFLAFSA
TVPMIMACEAYPIMLGNFAIADLVKSSVFLAIQFLCWAQTCMRLETMTDQHAAVFQALYN
TPWYEADLKYRKLIFMSLTYAAPSKYIKARLSNEITATTATFYSFVVSSFNWLNLIRKMS

>AlucOR96
MAKLFGFHIINADSRKCLSYRMSFPMTTLLMAFNLCMLSGNAIGCLIAALLDSNFDRRLM
NVKGMMLLIMIILLAINESFISRNRVNRILDYINRIKSITRYGFKEEEDIMNKAIDDCYK
STKYSTTFFFTNSMLMVTLPPTMAIITGESWKQLPYPWVITQTNDWLYYSSLVLQVVATA
LCHGVGAAGFSLLTTMKPLAAAFDKVILGINRIEERAARKMSEEGITYQESMLSCLKESI
AHHQEIVDELLMEKPHLEIMFFAQVTFISIVMACEAYPIIMGIVDVSGLIRGVLFLFIQV
MCCGFLNLEFDTIANKNVEVSEALYGTPWYALGVEYRHVVLNSMTFSQNPIWICGMGFFG
LRASRATFYSAMVSACNMLNMFRKFA
>AlucOR97
MVKLFDELAEQEDEELMGVYEKLYGPALQLSLLFPSWKRENLYKTFGIILVYTVTLLVHF
YVLSVSVVMLRDDFEAACLAFHYWLIFVMVFLSLALINMDRRTFSFAHRCLARDMGNYAA

GRIYSESKPLALEARKKKELFRFLVLPGMVVILAAALLVVPYLKKINNPPHYNAYGVNMN
LPLATHYPFPTDHGILHGVVVLGQLSAAFSLAVIVVSLELLLFRVSQAIIFEFKILQYAL
ETLFERSERLFFQLHPDYYGKLSHMNSNYQKCITRCIQDCVKHHYKIQELLQAYEYVLKW
PAALGYGIGTGVIGLGLVTLLMAKEKGNLENVVIFSLLIVAEVLNMYIVSVFGEDITTES
AAVRDELYFIEWYKLNIPNRRMMLNFQVGITNPVIVKAGGLVALCMDTFSSIMNTSYSFF
NLMNANPLDGNK
>AlucOR99
METKEDEYMKPLVQLLKFGGFWFDFSGHKHAMALKWCNIARNVIAFSVWAYQMAYFMGGV
SYLLTEAGVFVPICFDEGAVAVIVLCNQSTIRETIRIYRKRFEVFGSTPWAKNIIDSEMN
KFNRIFQLPKLMLSVFFLFYSVVPLVYDAVLAYSGKSPYVVPLPLNFLLETPMQRTPAFY
MTIYLSYLYFMIIVPRFIAFEALMMYIVAFVVIDVKILVQKMKNLSEKDDGSLFLQEEWN
LKDVIDHHSTVVRVVEEHFWLVGLAMMVQNLTFSISSCLVIYLTKTSFNNGDIVLALFCG
NFVVLLMILNLMFNGAGVLIENQGEMVLTAIYDTEWYKQPPKVRKEINAMLRQGLHVLKI
SYWSNTVNFETAMVVLNRAYSFFTLINTGE
>AlucOR100
MESVAMGEGEKRKTNRSEGCVDYRKFVFCRLMRTDDGLVKRGITGSLLIIMVTTVAMETC
SFVSIFMSKQLQSCLDCVRSFLLGNLVTMVLFNEFANRKRLARLHDFLNSSMSSPRTDLP
EAQEILNKAREEASSSLRMYIIIFSGNIAPMILAQPLAEWISGHSWKKLPIPWAFPPSDS
DVKFGLVFTFQCIGVCIANCLGMVFMSFSSITIQMTAMFDVLLLSLRRIENRATIRTQRE
EMDYKTSLLYCLREDVIFYQQLVREMTSATPHLRNTFLAFSATVPMIMACEAYPIISGNF
TIGDLIRSFVFLAIQFTCWAQTCTKLETMTDQHEAVFREIYQTPWSDSGPVYKRLVYTTL
LFSTQPKHIKARLSNEISATTSTFYSFVVSSFNWLSIIRKMN
>AlucOR101
MWDMRQLRMMNLWGWWPKMIKDPKKRKIMRVYGYCSFGLDSITMIAEIISLYLAVVNGSF
RGAIINIVTTTLGTMAAQKIYTMLVHHEFISHICDTLEDLDNRAIELMGEECQVTMKDRE
RRCLLTFVFVGSCMFTVCHYNVRPIIVYFLYGERTIAMDMWTPWDEQTSETGWIVVLIYE
WIHIFAAMYGMTVFDSLFLSIFEMILAEFDVLKIALRKINFAAEKKEVTLEFCIKFHQDL
LLLVARINEFLIPIQTIQCVMFTFTICFSGFELLSLSLSDGSLNKMANLVEVVGAATYITFG
YCYQCHCITEECEEVVRAACDNNWYEGSVEDQKKLLIILERAKNPISFGNIIKFDLGCFI
AIFKTAFSYYQVLQAFDI
>AlucOR103
MGTSTLGARPTMRPNRNLFSLSLLLMKIQGLETPSHATLKLVSFVWKYWMLYTALHFVMV
CLLGVTIGDNPYYLKLETCSGMIAGMSMVYRHFVLAFNRKEVHRLMDRINALVDDVVSVY
GEETIAPREKMCCGIMILSTCVVSFTTIPAYAYSYLKFYTDGEVTAPYEVYMPFERDAAH
IHHVVIFQMLSFLDQAITLIVSNTFIGTIVVIVSGVTEKIAKRYKEINRNNFHTLKVTTN
WHSEIIKIVEDTNALLGSAIMVDCLLSVVHISVSGYLLVKVGFESGTNLHKYIFLNLLCV
TIPSYFCLCGHIISVGRDKLHEAVYQNEWYELTPSDRKTLILPTWMADKGLSLHFKKAVE
FNLPTYLAIIKQSYSLIAMLKLMDG
>AlucOR105
MGFFTSVDMTDVQRLRVVEDSKRRSGLSDLIGRCGGYRGPLYNEHYSKNILFRIYVHLTD
LAVWINYITMIAAAMKSQSVLEFAMVGFPISAESLSLFLSYYSGYKNAEMTEVLLGFDDC
FDDDPYPQHLEMEIRKSADYYHHFSRSLLWLQVFTMQIYCFLFPVTNELMHDFFRPRALP
LPSLYPCDWKESRSCFIMIIFIHFLGATYVNWKIIGFGEVFFSMVSRQVALFRHLNHNLN
QILTSVHVSTDGTVIYRVDKEIDHIYIKSALRKWIKHHQNVMTQYDRLQTLYSWPLFVHF

GLVSGALCCSAYATSDETLDFDANLLCGGFLVGQMLELFYLCRMGDWITIETNELTLALT
GSYTFVLDRIESQMLRIILSRVHRPSVMRAVGLYPLNTSTLKMLIQSTYSYYTMLKKVNR
G
>AlucOR106
MKEKDHSKRKDMLSVDYRKYMYCKLIWIDDGLADRGLTTPLLFIMIWMVFMVFFGLVSFV
MSTQNKVRLDNLRSFLLESMVTMSIFNEYMSRKSLARLHHFMDESMKTPRTGLPKEEEIL
KDAKSLAKKHLTAYTVIFTFNLAAMILSQPVAEWIQGNSWKKLPYPWVIPPSHNEFLFWI
VFMFQSIGLYFSHCLGVVIMSFSSITIQITALFEVLLLSLSHIEDRAKVMSEREGADYTT
CITECLKEDIIHHQRLARELISATPHLRRTFFALSVTISMIMACEAYPLIMGNFTLGELI
KGLLFLVVQFMCWGQMCTRMETMADQNTAVFNALYGTPWYDSGVKYKKLMLTSFTFSREP
MYIISPLFIEMKATMSTFYSFVVSSFNILNLIRKMN
>AlucOR107
MEESWLVRYYGGGLGQAEYERVRDFAVSEFTPLVLFLGIFPPTDKMALMSIVVSLSIAVY
AFYIVLFTITCSFATDDFVLWSELIHHTSLMYLGIFIRSVLILEAKEMIKLARDYLDGIY
HYEEGYVDPIFQQLQDKSRKLQRKLFMLPLFIVLVTGIALGLKPLLDDVNEVEPHPKLLE
NGITYRSLIPIFYPFNNENTYQVLLMNGALLYFAFLVVVTVIAADLLFIRVSCRISLEIA
ILVESLNLIDKRAKRLYARKYGLNKKNESWPLYQDCIEECIKENVKHHQKIIIFYEQFSA
VAAPAIGGGFFTCTIVLGLGMIVVNMDNVNISDIIAFVGTVFAEMMNAFMISWMSEKIGE
QNYELYNAVYNLKWFKWRQSNKKLVITFLDGTRQPLFLNAFGMATINMEAFGSVVNTAYS
FLNLVNASETLEEKK
>ClecOR2like_isoform_X3
MNENLKKLEVIRFIMKLSGQWQEFDSFSTVKVFLQRFRLCVSAAFIGPMAYAQLDWGKSK
SVYDKLKEMLARRDRQEEIDFIKERTRLIWFCVKIFGTCSTCFITTFDILPYFYDFYLYT
AGVEKPFMVPLPNTGYLGENPKRSFYYYFVNVVTSLWCAKISVVAFGYESLGYLSISYAC
AELEIISMRIKKWGELRKESGIELRDIIDEHRQILSICRLAHELKELFGMAMASQNVVGA
LSLTLYAYTIMVELGEDVLQVVVNAFCLVIVTALISAANFMGQNLQDKSMELFKSLCDVP
WEEMEPAVRKDLNMMIRQAKRPMVVDYRGRSPLNLVTLMGIFNASYSYFMMLKSMY
>ClecOR2like_isoform_X2
MNENLKKLEVIRFIMKLSGQWQEFDSFSTVKVFLQRFRLWYMIAVTPLTFISLFLSGFKE
SLSKYSLFSVSAAFIGPMAYAQLDWGKSKSVYDKLKEMLARRDRQEEIDFIKERTRLIWF
CVKIFGTCSTCFITTFDILPYFYDFYLYTAGVEKPFMVPLPNTGYLGENPKRSFYYYFVN
VVTSLWCAKISVVAFGYESLGYLSISYACAELEIISMRIKKWGELRKESGIELRDIIDEH
RQILRLAHELKELFGMAMASQNVVGALSLTLYAYTIMVELGEDVLQVVVNAFCLVIVTAL
ISAANFMGQNLQDKSMELFKSLCDVPWEEMEPAVRKDLNMMIRQAKRPMVVDYRGRSPLN
LVTLMGIFNASYSYFMMLKSMY
>ClecOR2like_isoform_X1
MNENLKKLEVIRFIMKLSGQWQEFDSFSTVKVFLQRFRLWYMIAVTPLTFISLFLSGFKE
SLSKYSLFSVSAAFIGPMAYAQLDWGKSKSVYDKLKEMLARRDRQEEIDFIKERTRLIWF
CVKIFGTCSTCFITTFDILPYFYDFYLYTAGVEKPFMVPLPNTGYLGENPKRSFYYYFVN
VVTSLWCAKISVVAFGYESLGYLSISYACAELEIISMRIKKWGELRKESGIELRDIIDEH
RQILSICRLAHELKELFGMAMASQNVVGALSLTLYAYTIMVELGEDVLQVVVNAFCLVIV
TALISAANFMGQNLQDKSMELFKSLCDVPWEEMEPAVRKDLNMMIRQAKRPMVVDYRGRS
PLNLVTLMGIFNASYSYFMMLKSMY
>ClecOR2like

MAGKEKGEIKEKSKVRHYTQCKFYKENYGRYMSWGMFLPMSRMQRPLYFFIGTIHLPVVF
YTCYLILEEEFADFTELIHFGILLILSTTVTSFFNSAQLENLIWYYESGLGDYGDTIDKV
SAKEIKKVKSKFLKRKNFHSLFMYYGLYMASCSLGLLRPVYEWLSGKYDNISFKDNVDRM
LPLSVWYPLDVTSWPVHIFMTCLEYYIIVQTTFLVIGSSIIYISVSEELCAQIATLKITM
KNITRRAKHVGKGVTTKENLRECLRRCVIHHCRIIEWFDMFQKFYSRPLWLIMTILTYMI
CLCAFITTMKADLTFSNRVTSVAFLVLEVFYLLVYCWYGEEIRSESGDLHFEIYDSDWID
FQVDIKNYLLIIQQRAYHPLKMYGGLISADLNTFSTICNTAYSYFNLLNASQN
>ClecOrco
MQLTGHWLLEYHEENGGIMRLLRLAYCWITTLLILVQFGFLVCFLILETYDADQMAAATI
TTLFFLHSVTKYLYFALRSKYFYRTLSAWNQVNSHPLFAESNARHRATALSRMRKLLMII
GVGTIFSVLAWTTVTFLDEPYRDITDPDDVNSTITVEVPQLMVDAWYPWDARNGMAYFLT
FIYQLYWLIMSLSHANLLDILFCSFVIFSCEQLKHLKEILQPLMELSAALDSVVPNSGDL
FRASSTSSNIPLIGNDVNEFDVRGIYSNQRDFSGFQGGAIPTNGGIGPNGLTKKQELLVR
SAIKYWVERHKHVVRFVTSIGDCYGSALLLHMLTSTVTLTLLAYQATKIEAVDVYASTVI
GYLLYTLGQVFVFCIHGNELIEESSSVMEAAYSCHWYDGSEEAKTFVQIVCQQCQKSLTV
SGAKFFTVSLDLFASVLGAVVTYFMVLVQLK
>ClecOR92a
MVSEGIVKAKYCYYSALGITGLASYLDGNKPKLTTRIWTCFFIYVILPSVTTAHFYTSLF
SETSYAQKFLAMSLGLDQFQIMTKSIFFHNYMSKMSGLVLDFSSFACVGHDWERTGTILG
KRTDEILRLQKFYNMVLLVCFCSWSLVPIISSPSALFLSRNLEEMSKVLPIVYPFDVNSF
PWVQIIYVYEVVSTMGILIHFAGSNLFFVCCVITLCGMLELLKMSFETSNNYEDFKQFIK
DHQRLLCMCKEIKHILSPILALQLLISALTICFAIYEITMVNETSKSGMDQVILYSRKTT
YTMVIFIELLFYCWLSTELQQATLTIRDGVYNSSWYLDKQPKYKDIVIINARSLRPVSLT
ALMMNNLHLGTSIEVLRAAYSYYTMLKQMNR
>ClecOR85e
MKYSGLWKIINDYRVTGKKNILLKIGLLISFLFPGHYVMFSLSSIFFSEVDFQAATMFVL
NPCSGLQSVLKVLVFWFGLHSQCQILNMMKKDFLRCIPKDKAEEAKEIMKKIAIRSNYFC
IAGISANAICVLVWNFIPILRSQYFREELNIKLDNMETNTPNKILGGGYPFNYTITPWSE
CVYVFEFIVCAWAGFVISLHECILIQLVMLLWGHIQVINFVLSNIRRSDFLVKRNLPTRL
SKIKEDDINSSEAVNKRMNDHLLNCVKDHQLTMKVGDQIRDLYNFLITAQLGTGLLIMII
SVFHFQYYGTKDALFTVKFVVYLGYCMVENVLYCYCGAYLEAASEEVGMGFYSGEWYKGD
ANFRKIGQMLMVRSLRPVSLIAVQLYPVNLTTLTSLLQLTYSSSALMSRFVNE
>ClecOR85clike
MNNVIDPILKYSVAIAGDKKLEVYTLYFSFLNFLGFDWKQGGINVPVPAWVKFKVYPASY
PIMMLIVSFLIIKLNVEVEPKTEFEMIKMINGFIVSMTFIAAFAKLLYLRIIKNDILELL
NMSVTVGPISAEKPVVKDTIRSCVLYFILLNANVGTWTVGYLWLHNDLPFAAAFPWDEST
VVGFSLSFNVDVLCAFYCSIGHTVLDISFPLVVSTMCWHIQRLEKLLSTLGKDYFIDRMI
LDKAVDLHVRLLRQVIVCYNQLIVAQSVYTVLHSCILIYAVVKVSNKVEAALSSVPMLSA
SYAQLYMYCSYGQLLTDKTHTLSFAAYNNEWYRCPPKLRKSLVLFLEATKRAVHLKGFGN
IQASLANYLHSLQDSVSYFLVLKTLTSDENA
>ClecOR85blike
MDFIFKTSNRLIVALSCEGRLRIFTLQRVLLKILGFEWWKEGLNFPLPDFMKKWGYQAFW
VILMICDIVLTLKASVWMAIDNDLKYTIKMNGIIVMMPFVGSLIKIFYLRYMQKEFIELI
ETCESIGHLDMEEESIQSGLRLNFVYINMLLSTVGIWAIFFTIINHEIPVATDFPWSQDV

ALGQFFSLMIDFYCGINCCLAHSLLDTIFPVSAIVIIAHLATLRKKLAKLGCNKYQDEVL
LNESIELHNKLLSMSELLQICYNEVSVAQSLYSVMHSCVLIFAVEQVPNKFSVLVSSFPL
LLCSYAQLWMYCSFGQMMTDEFDKISFALYNNRWYACSISSRKALVTFAQACQKKVGLKG
VGNINASYSNFLHKIQDSVSYYLILKTVTSESDGEKN
>ClecOR69a_isoform_B_isoform_X2
MGKEKSSILLLKNLLTGIGVLPLTDGHSKWIDRFMIEVPIPIIDKLETLLYITASIHIVS
NQAILILKSKAVKILIEDIENLYNILYENENNASILNKWSNRGKRQAYFFISTYIVYTCC
NCLMSILHLLIFNVYKSPYSIGTLFVPKDLKALAMVFQVFTMFFGTLIMSIHMSFICIIG
TSIAGGMEVLKKELQARSTTDNRDFHLFSYKLHSQIINLTARFNKIYGFPLAILTAFCSI
QCCLTTYPLARANVRAQDFVLFCTTNVFPVAICETGNAVFTESNNVFLATYDNYWYNESP
KSRKELSIMMLVARRPLHLHFRHVVRFTYETYLSILQTSYSYMAVLRTMERS
>ClecOR69a_isoform_B_isoform_X1
MGKEKSSILLLKNLLTGIGVLPLTDGHSKWIDRVWTLLCFCCGSFLLMSSFQFMIEVPIP
IIDKLETLLYITASIHIVSNQAILILKSKAVKILIEDIENLYNILYENENNASILNKWSN
RGKRQAYFFISTYIVYTCCNCLMSILHLLIFNVYKSPYSIGTLFVPKDLKALAMVFQVFT
MFFGTLIMSIHMSFICIIGTSIAGGMEVLKKELQARSTTDNRDFHLFSYKLHSQIINLTA
RFNKIYGFPLAILTAFCSIQCCLTTYPLARANVRAQDFVLFCTTNVFPVAICETGNAVFT
ESNNVFLATYDNYWYNESPKSRKELSIMMLVARRPLHLHFRHVVRFTYETYLSILQTSYS
YMAVLRTMERS
>ClecOR69a_isoform_B
MKFGRYYIERPSNTIYHLYELGGMMVSPGSKHPYLVKFNIVFVGLGMFYFLTTQLLTLYT
KIGDQLVIMQFTISTCQIMFKHCMIFVRKKQIVDFISDLDVMWEKVEADDVHKKLFNETI
KGRVRYANTFMLMCFCVQPYNVLFWLLTIMTKPREEWEDWIVWWNLVDAEHFWLRFIVQI
YCFEAVMISEGVTGSMVTYLCAHISGYTRVLRHMLESRLPDNKSQYKETYIFHQKIIKLV
RKLNSIYSVSYFVEIVTSSLTVAFRSFFMVKLVATNGIGGSFAAELLLTILEFFAPYTIS
MSCELIKSEGEEIIKSAYHNSWYEEDPSTEKDLLILSAFATRPLLVRYKTAFEFSMEKFS
TFVQATYSYIAMLKNMNF
>ClecOR69a_isoform_A
MGTVKTELTFEDLTGFRALPIQMSLVKLISLMPNSLNKITKFIIDILLILYLIAGTIQTY
SIFGGWKEAYNQKHFLKVSNSINYTVLFIDLLQKITLLRFNTSAIETLGNWAKKVKIRIG
NNETNLIKVVKIVNKFHSVTSNIAALFWAFYPLITGKTILPLKYPFLSQFLSTISFPVEF
IIYTGFSTVISYGAAVFLYALALFSTEIHRLADDWASLTYDSKQPQLYRQQFKLLVERHV
ELLKVLVNIKKLHEGAFGFQIIITMLLIFTGTFGIIKLDDDAVTLIIITTPFGVLTTSEF
FLMCWIGVQITDGFSSIHQKLYFTNWYDTPANDKLSLQIVMEFSKKPIILTGVKIYTASL
TTFGNVMKETFSLYTVLKALMG
>ClecOR67a_isoform_X2
MKSECMKFTRLLLQISGFIPINNSYGYSFSFKLISLNGKVAMLVYSFISFMFVVQSDLTE
SFLAMTIFCMSVQLVLKAYMTSKNNFFKMLDEADQLLENVLTDQKAPLKNPYQLWSPFSS
VLLSYFFQSYILLMAGIIETSYLDSIASVSLHITGCLKILSHRLSNQSMFVSKKVERESI
KIHSDVIRLVASMNKNYGAMLTIEILFASVQACLAGYQIMVGLENLHSNLLVFFITFIFV
WLLPSMICFCGQEIETESENIHRLLHYNSWFQRSPENRKTVFFQMLMMSKPLKLHFRNFI
VFNVAQLAGVLQSAYTVMTMMRLFFN
>ClecOR67a_isoform_X1
MKSECMKFTRLLLQISGFIPINNSYGYSFSFKLISLNGKVAMLVYSFISFMFVVQSDLTE

SFLAMTIFCMSVQLVLKAYMTSKNNFFKMLDEADQLLENVLTDQKAYDIFEPMFTFIPLH
FLQIVIDYFSDRPLKNPYQLWSPFSSVLLSYFFQSYILLMAGIIETSYLDSIASVSLHIT
GCLKILSHRLSNQSMFVSKKVERESIKIHSDVIRLVASMNKNYGAMLTIEILFASVQACL
AGYQIMVGLENLHSNLLVFFITFIFVWLLPSMICFCGQEIETESENIHRLLHYNSWFQRS
PENRKTVFFQMLMMSKPLKLHFRNFIVFNVAQLAGVLQSAYTVMTMMRLFFN
>ClecOR4like_isoform_X1
MMWKVASYIANDDRKTGFTYHKAAFKLLGLFYWGEKNWFQVFYEWYPIVNLGIGATLLTI
DVIQKGQSASSKAFIELVKGSVLVFTLYFAMVKLVLFQLYSKEIRQAIDFLESLPKIGGT
DTSANTRKTLVRYSVLLLTTGCGWAFFNSIHTGSPYVCYWPFETPRPKIFFLLLAFVQKL
MVIGYCVSAHSAVDTSFPMLVTYMAYQGTRLVSLLETLGKNKYEYKKFKEGVNIHINILK
SMGYVQSAFRVLFMCQVTYTIVHACVIAFSALKVGNIMFVITSLVPILCSAYVQLFIYCQ
AGERMTDIMDKIAVAVCDNTWYESDLKIKKSMVLMLQMANKSVTMNKLGVYTASLKTYLN
SVQESFSCFLALKTVSSKL
>ClecOR4like
MVILNLILGILFTIQITIQMWNNILAGYEFSTFTEKFSVNLTCFESAIKMLYYCSQKTSL
KFLIKSFNSNFLLCSKHNEEATERVMSKCADFVNRSTKSFMYMIFSTVTVWNALPILKCI
SGDCGSWQIMPSWYPFDTTYFPVNGLIYIFEFYVMVFCAALLYNVNCLFSALALSIAAQF
DLLVTSLSSIPQNAERLAKKESKEEIMSLLLRDCLMDHQNLLRLTKELERMYNPMFLFQM
LTSTFTICLVLVQLNEYLGASDQLSIPLACKFIMYLVFGSAELFIYSWGGQIIYDKTSNV
HRSLYETGWDQASVKFRKCVLFAMTRSYRPDSLTAGKFYTVDLNSFTQTIKASYSYFTVL
RGSDSKKH
>ClecOR49alike
MDSLKSYNPDTFVTKAIRKLLTSYSLMMSGMILLWVCTPLVFLQRRIPMYFSAPWESESW
LGFITEYILASVCLLCEAHIHTMMDSFVMLLSLQISHRLYLLRLSLEHIGSSGKSEMEGM
EGTIYKSNPLTTIQLCVDEHVFIRRLMKAYEGLVSVVFLFQVLISTVIIGIVIFSVTTLE
KTVNEIIKYVPIFGLVYSQIFLYCWSGENISTHYNNLSFACYSSKWYQLPVTHRKQILKF
QLNSTKPVKIRGWYITDMALSTFLASVQQSCSYFMILYQFVLEK
>ClecOR47alike
MQSKCIKFARLGLQLAGIIPMKNSYNHFFSFKLLSFNGKLTMLVCGIISFMFAIQSELTK
SFTAWAIFSVSVQLVLKQYTTHKNDFAKMLDDADRLLESVLTDQKAREIFEPMDSIRIKF
CKVFVYSLLCYMPLNFFSNVLDYFSGRPLRNPFQLWTPFSSILLSFVFHSYILSMAIITE
SSYLNSIASVSLHVVSCLKILSHRLSKQPQFISKKVERETIKIHSDVIQLVALMNKNYGA
MITLEILSASLQACFTGYQILVGLENLDSNLLVFFVVFIFIWELPFIICYCGQEIETESE
TILRALYQNLWHQRSPENRKTVFFQMLMMSKPLKLHFRNFIVFNFAQLGGVLQSAYTAMA
MMRLLFI
>ClecOR46a_isoform_Blike
MNENMKDFDMIRKIMVWAGQWQEFSTYPRLKIFLQKFRVTYLMVLSPLPLTSMFLSGFMD
SLSKYALFAVGATLIDPMAYSFFFEWDLAKKVYTRLRAIVARRDSEEHIKRFKTKSRPIW
IVVAVYALFTFLFVFFYEIMPYFYDAYLFLFTGSDKPFMTPIPNQGFMDKEPKRTFYYYC
SNVFTTLWCMDLAMIVFGFDSLTYLVVIYTCIEVEIISERVKSWGEKGADPAELREIIEE
HNEVLRLTDDMIELLGGPMASQNTVGSLAITIFAYTVLVKYETDLFLVIVNGLSVGLTFL
VIATANYVGDRLEDEGVRFFQSLYDTPWYNMPQKERKAIRIMMCQAVKPLVITYKGWSPL
NFVTLMDVVNASYSYFMVLKSMN
>ClecOR45blike

MERLEESDLVDGLSVNYMKYSGLWTIINEYRETGRKNAMLKIGVFITMIFLCPFITFSLS
SFFVIEVDFQAATMFVLNPCSCIQSVLKVLVFWFGLESQCQILNMMRKDFLNCIPKDKEE
EANEIMKNIAHRSNYFCVAGITANTACVLVWNFIPVLRSQYFREELNIKLDNMETNSPNK
ILGGWYPFNYTVTPWSEGVYVFEFIVCAWAGFVISLHECILIQLVMLLWGHLKVVSFVLS
NIRRSDFLVKKRFSAIHSFKNYEDDSINSSEAVSKRMNDHLLICLKDHQHLMKVGDQIRD
LYNFLITAQLGTGLLIMIITVFHFQYYGTKDALFTVKFVVYLGYCMVENVLYCYCGAYLE
AASEEVGMGFYSGEWYKGDVNYRKIGQMLMVRSLRPVSLIAVQLYPVNLTTLTSLLQLTY
SSSALMSRFINDN
>ClecOR43alike
MIIVCILFMVKYRSVYSEIDRIIKSGVYDYGEDFTEEQQSICEGANKLVLKLGKLLTIAF
IATVSTSLTKQTIFRRKPGSWNTLFKGWTPFVINTWPRYLIVFIVHIIMIINTSTSGYLL
VLSFLTFGVHLVAQLKSLRIALQEIFLSEKNQSEEQILAKLKKCAAHHAEITRFFNLVQV
YSGSVSGVLPIGSVGMICTFLYDMTGENTVEASFMVILPEASLITTYALIGQYITNESEN
LRTFLYDIDWYAQPVSVQKYLLLMMMSSKKSLDSKGFGVFKYSLEGLTEVMQTTYTFYNA
LNATR
>ClecOR30alike
MDYLPWQLVVLLWQQQVISAMLFTVYGVLGALTIFYDYISNEISLLKFAMLHIEERAKEM
MKTTKMKYQNLTEQELYKKCLIACTNQCAKHHSIIYDYYLNAIQAVRYTYFIVVIVGIVA
LVCGGATIIFGSMNIKTKFFGMIIATLIFLYLAFWFGNEVRDLSDTIPWIVYDINWLELP
KECHSTLRIMMTRSMKPMALTTSLGQNVDLENFMGLVKAAYSYFNMIYQGSQQGMF
>ClecOR24like_isoform_X2
MLFLKMDYAVTYTVSGLMYDHGFSPSNPKQAWMDKLEKTVLKRPRKVTESAFYLEMRPLL
FLLRLFGKISYMINKEGKMEARLFSISSLSCLAVFAGQTFLVARNIVTLVEVLKEEENFG
RFVQGFLILTFMAFHFFLPFSLYLESGKICHFFNEWAVFQDLMEKTTGHKFSTNYDKWLR
ACLFMCPLGVIIIVLYERNILYNSVWYQLIFYAILLMIFQISLYLWIFSLIEIGYAAQVV
QKELKKTTVESCTGLNIYNYRLIWLKLSKLLEIVGDAVALTMIAMTTVNHTCFIISAYML
ISSFMHSLYDSIPFLTIMVITGLMITQTFEPGEFVSRKLGKQIADTLMETDISKVDSDCL
KELNLFTQAVSGSNNVVTFGGFANVNRSALAGIVGSTVTYLIVLVQFNQSPES
>ClecOR24like_isoform_X1
MLKRENSIKHSAGTKPGSNETVCEGYLGSSGSTVNIMSVSGLMYDHGFSPSNPKQAWMDK
LEKTVLKRPRKVTESAFYLEMRPLLFLLRLFGKISYMINKEGKMEARLFSISSLSCLAVF
AGQTFLVARNIVTLVEVLKEEENFGRFVQGFLILTFMAFHFFLPFSLYLESGKICHFFNE
WAVFQDLMEKTTGHKFSTNYDKWLRACLFMCPLGVIIIVLYERNILYNSVWYQLIFYAIL
LMIFQISLYLWIFSLIEIGYAAQVVQKELKKTTVESCTGLNIYNYRLIWLKLSKLLEIVG
DAVALTMIAMTTVNHTCFIISAYMLISSFMHSLYDSIPFLTIMVITGLMITQTFEPGEFV
SRKLGKQIADTLMETDISKVDSDCLKELNLFTQAVSGSNNVVTFGGFANVNRSALAGIVG
STVTYLIVLVQFNQSPES
>ClecOR22like
MASKRKHTDKPNDPFYLEIRPLLVVLRAFARLAFGIKDGRLVSFPRSCVSLIWFVLYGAH
FYLAVDVFLIAFRRLKTETSFFLTIIMMISLILTSVHFYLPLSVVLESGKICSYVNSWAD
FQDLFFLVTGTRFRPKYRKLLNVCLFLTPLHQIGVLWLQQKIQYFEQWYHMSLFFSILLI
ANMNLLFWIISFLEMAHAANLIKEIIEKFQYNFQSKSVAKLTVLWISLVKLIGRLSDSMY
ITMLVFITVVHSCGVTSAYAVISSINSGSVEDVFIFLMLLLLSCVLILAVIEPVHLTKIK
VHDEIYREIIKIDTKKIDPTISKEFERFGEVVQKMNLKVTLGGFITIHRNLMTSMIGTAV

THLVVLVQFSTRQENSSS
>ClecOR21alike
MTFLQHLYFQKSNNAFYLEVRPILLLFRIFARMTYDIKDGTLICRPRLIASLAWFILYGA
QFYVTIDLLMGLTWHLKQEKNFITSIVMTVTLVFILMHLFLPISLILESDHICSYVNRWA
DLQVQFYKATGKKFIPKYRKLLYVLLVITPFHEMLAIWISQSVLYYDRWYHLVLYFSTFF
VCQLNTLFWAISFFEMAHIASEIKEGLRTNFRSFGGYSNIANLRSLWVSLVDLCADLGRA
LWRTMIILMIVNFGSSVASVYAIISHFISGSPGIWTFVMVLFCNGTAILLMVEPVHMAML
KAGHGVYQELLEFNVTKLGGTSFVQVDKFLQVVKGINPRVTLGGFFTIDRSLLTTIAGAS
VTYLIVLIQFRIPSENM
>SfurORco
MEKVRKHGLIGDLWPHIRLMQLTGHWLLEYHDNNESVMVLIRKLYCSLTTFLVLLQFIFI
FVFLCFNTYDADQMAAGTITVLFFLHSITKFLFFAFRSKYFYRTFSAWNQMNSHPLFAES
NARYRAVALTRMRKLLMIIGIGTILSVFAWTVIIFLDEPVRDIPDPNDANSTIKEEVPQL
MMYSWYPWDAKTGMRYMLSFGYQLYWLFITLSHANLLDVTFCCFVIFACEQLKHIKEILK
PLMELSAALDTAVPNSGELFRASSTTSNLPLLGSESEKAGSDFDVRGIYSNQRDFSAFRD
GGGGGVMSVSGGVGPNGLTKKQELLVRSAIKYWVERHKHVVKYVSSINDAYGAALLIHML
ITTVTLTLLAYQATKINNVDVYAATVIGYLFYTLAQVFVYCIHGNELIEESSSVMEAAYS
CHWYDGSEEAKTFVQIVCQQCQKSLTISGAKFFTVSLDLFASVLGAVVTYFMVLVQLK
>SfurOR1
MMKGDSDLKNGPVSENENDEPINSEKLISYFGDKRIVGFKGMPYLVVTFLIILDLIFCLN
YEWPFFLRRILTIKEICFAVFALGGFFVQYDYYAVEKNYILKYLKSVDQHATSEPKKKII
REANDFLKYHSVFAVRAFSFMFVFAAVIPMLQVVIIVFKSVLKHEKIQKLPFVIFLYMPE
GFRSPLVYLATQMVAFTWYALAMLMWGIGYKVYLIAMKCICAEMKLLSQSLMELDTALVT
NKVEEHLDAVGEDLVIGAENNARLKRYLQKIIVHHQDIIKSMKMLNNEFQLTIIIFINIY
SLQLCLYIIFIMKMTSIIHRMKYFFVYCVVLIIQLQWATFGQDLINNGDRLRLALYESSW
MNKPLWMKKSLLIMMARAEQPLEFKPYGLYVLDKQYMANLFKATYTYVNLVYEFLN
>SfurOR2
MDITRPLRKIINYLHCDSLGTSLPIIFITSLCIIVIICSIVAALEDWRDRRDVLLALKDI
LVMIALVLCTLEHTFSPGKINRLMDIIEEEFLVGRHRSLKQKSPKTTKYHEFFENSHAEM
KKLHNNIFLTLVVIFVATLLKRLIRNWMEVLDNKVPDDWPTPYLFYYPEGYSAVSLFTFA
FVLHSVQLWFMLLAAYCILVSTAIATTKITADFKALCLYLEDFSKEYYFLAGNEVDYDYF
NKTNSFENGLAYMINVDSNNNDEIPTAKNDLGEFSKTEQDLRDDMREIVKTHQDLYRNLK
TLEVNSGTMTLLIDFAISIETCVGLFVIMKTDDSVTQMNMVVAVVAIDFLLLFTCNKGQL
IINQGYDVRFQLAKCPWIDKPQWFKKSLQIMMTRSNLDTELNPFGLYVLNYESFKNILKA
SYSFCNLLNAMP
>SfurOR3
MLWQNTEENWKSVRAGLKKSHMKFVVGSGLLIYPEQPWLACNLTMMAIGLSVSSFFVWVG
AVSLYYARHDPLAFVEIAHGYSIVLALWVLITRHYIFTMPILHEILQIIDTDVFHYKTVY
GKEEDEKAIQSVKTFFEDVFQRGFYHIITIGTAILCVAPGILSMYGSEDRKQIKELNYDF
PVPFWLPFNTDTRQGFFMGYLLVCLIIFFVALYLSAAIPFLVHSVLLLGLQYEILERSIL
NVESRAIEMYGWVYGSQDIKPEILQGDPLYEKCVHECLRQNILHHIAIVRYLNLYKEFTS
PIFGVLIGVSLIILGSLSVVLTKTKLFSFETVKFAFFFIVELVIVAGYCFVGSYLTEASE
KVPRAIYDCSWYNLSTRHRKTMLIFQIRTGEIEQLKGSGLFEINRELFVQIVRATYSIFN
LFSSMDN

>SfurOR4
MNQFGLDENSRVIYKAPSPKYIGVNSPAKDVTRLSRFLVSFVDGKNTLSWSIATIFRIYL
LAYHIDLIAGVIKLRDFTSRFLVIKELNLIWFALAVSKSAAEVTMLCDMIDRLSISSDHL
VVSSLANAKKRELIKEREDKLVESGEFYASLLTCAFVLTGLLPFCQVVFWFFYYTFSSDE
EIEFVDLPFIMFVYYPDSYKSLNAYFLGQLSTFIFLHITIMHYMLPVFTCVSQTIICIVY
EVKLICLRLEHLNDLTVYKNMNVLLRVHTSALIKSHVLLASDIESLNKGINGLAFAYMNV
MALQICMSLFCVLEFDDIVIRLKYGFCIVFIFLVMSVCTSFGQKIIDVGDVLRWELYNCP
WLEMPNWFKQSLLLMMRRSFRNMELKPYGLYVLDMRYLTNIVNATYTYFNFFLKA
>SfurOR5
MNIDEQHTPSHLELKESVWKRTQFTLFPKITVTEVIIAVIVTYFATQTALLLILHWDAYD
INSRPEVFANLSYNLFVFLVTMEVFFINGRLRIFMQIIDSEFKISGELVEKKRMKIDELE
ETSLKNSKALSKVLLFFSIGYSFNCIHGILMCVYKLRPIDELPITFSFLLPANFTKTKES
GYFYVCIALLQVWYLYFGYIIVSVLYTSRLFAYNSIFTEIELFLITLEELNHFRFENEEE
SNFESRVIEQSDVVRLKEIIRTLGKHHQVIFKKISMFEEGGRYHIFYVNASICALLCLAI
FCTQRVTAHNLKVRYALLALSMIAGCMIYSENGQRLMNRGEDSRKAIYECSWIDKPIWVQ
KSLLIMMMRNTKDFDINFYGIFKSNRSNVSSLLQASYSYFSFLNNTN
>SfurOR6
MDGNVQSNPVVKRVRQLLSLSGMQPPQKGTELSLIFTLLSFYFGFETCISLIKNWPLIHS
RLLALEDIIISIIMIMCCWEFAYAPTHIQCLLNILDESYFNDDDDETESEQKREIIKKAM
DRERITAKIIETLAIITGINLTALPIFVLIKKLVVGESLFENVEDVPLPFEYSYIFVECN
LSSFLIIYVVQSIWFLMSAPAFACCVYMVTVQAMRRVKTELDLLVISLNDLDKSIGHDTD
LGDEYVIEQKLKSRLKSFVRHHYFIIQRVNELNMGLKLTPTITLQAFCFLICSCLWYIVK
GEEIVLKVKYVSFLIPLLIYLFLYAAAGQDIINGQEQLAITLFECSWMDKPKWFKTSLAT
MMLFVNRPLYVKQFGIYKLDMPYFSKILNAGYSYFSLLNTVQ
>SfurOR7
MAQITAEATKKMRKFLMQRGHFERKSMRVLAKFYLLLTVVLIGNTLQDIYYLQADQFEEK
VFRFKNINMLICGLAYPLLHKSTYRLISYVEEHIRPPDLTFKPTLARSQLQQQMNADLNY
VDRLTHTVVITIVATLSIAPLFTAIFKIYRQNSLGEPINVVQLSLPISFWYPEKYASLLV
YFSLFIVQMFYVYLFAGYVYCDLTSSFMALKITIYDLKFLNLTVEEWDDNVGGVKENNGF
DEGKMEWSRNKNEGSMEWNNKENIGGAKNWNNESDKGRSMNWNTIETTGRAVNWNNKSEA
GYVEYNKENAGFYDSSSLLRNNIVSFIKLHDMICRRSSSMNKDFEMIYTIYNNTICFQIC
VCLYTSAKENDIILKAENILHIIPLAGILFLYCFYSQEILNEGERFRMKLWNSSFIGKPK
WYRSSMLIIMIRNSKELEMKPFGCYALNLETFSVVMKAAYSYFNMLNSLKKRI
>SfurOR8
MDQTITGASLVSCFGEPRIVGFKGALFFVLTVLISVDLGFCLVYEWHDFIKRLLTLKEIA
LVIFCTGGYFAHLNMASVENEYIFKYLKYVDEPSHSHSPKKLELINEMNVFIKWLSAFST
RAFKFLYIFAAVIPLTHILIVLTKAHFSHKKLDEVNIPSVIHFYVPMKLRTASGYFLTNV
AASTWYAFCLYIWSIVFKLFVIGIGCLCTEMELIFDDVNEIDHIPPAAETNPSKPIEGTM
HVNEILLKRKFNSIIEHHQDITKSMKLMNRNFQTTIVLFLNVYCLQLCLYLVFIMKLEDI
VQRIKFLALYIFALYLQYQYAACSQRIKDRGETFGETLYECGWVDKPQWMKKMLLIMKCM
ADQPMVMKPYGWYVVDRVFMANIFKATYTYLNVVNEFLK
>SfurOR9
MEDRVSSESVISIFGNKRVTGLEAFIFLSLSLLILLNLIFCLIEEWSDFFARLLTLKEIM
LILFSAIGGYVVQFHMRDVEENYILKYLRNTKNFETTEEKRKIIDGTNNYIVRHNVVTLR

AFKFLFISGGCLPLLQITIKTLKQYLTGIEAEKMAFVIHLYLPEGYKTPLIYLLTQVAAF
ILYGLLVYMWGVNYKVFLIGLKCISTEVCLLVESLKELESFEGKSAPAELAENEEQMLAV
SGFEERKLREHLGRTIEQHQDVLQSLKLVNDSSKLYIFLFINVYSIQLGLYIIFVLKLKE
LAQKMKYVFTYMVILFIQMQWAIYAQDIQDNGEKLRMALYNCSWLDKPQWMKKSLVLMLM
KANKPLELRPFGLYALNKSCMANMMKATYTYVNVVNELLSRK
>SfurOR10
MRKNLEKRDSIKLQTYSTAPKVSKLVNILNRDEGQVNILRRLTELIIVLMICNLSFGIFR
FWNDTIKKILAIKEINLAFFALCVQMSTTEVLPLCALVEDYCTKIIFYYNENSEKQRSIR
EERLVKLKRLEDFNIKFFITLVGFSVFMPCLQSIYIILIHNNYDDIQDLPFVLFSYYPEK
YKSLTLHAIVQTGYGVFLIINELQMYCIFTAMYMASQTLTDEFDLLVTCFEEIDANIRDY
DLVKKHFKDDVEVVKYQSSYENKLQTFLSKIVDHHSSLYSNVEVINKRISILCFGTINAF
TFQLFTCVMCAIEIENFATRVKYVFFSISIIFLMILCAAIGQNISDKGEEVRNALCNCSW
VDKPEWFKKSILIMLIRSSKPLLIMPFGLYVLDLRRFAVILNGIYSYFNVIY
>SfurOR11
MEFEGEREHAREIEKPSYFKTSKKPAEILLKLYLLVTSMMIINLLFCLKIEWNSFIKRLL
AFKDLNSMWSAFVAYFATNDAITLFKYIHQFTGQCALHHEDSRNLKEFKARLDVVDKSVS
KVFMTFVVICSVLPYCVGIIMILKSDPSKISAQRLPFIMLVYYPEDYRSVNVYLIVQTII
FLWYAMIIYLWCMCLKSMLLSIECVTHQVNSICESLKKLMKLLHRSSQLLLLKQTLKVTT
QNLLKRII
>SfurOR12
MRQILVQRGHFEGEQIRLLAQFYVVLTIIFISNTILDIYNLEANQLEEKIFRFKNINMLI
CALAYPLLDKSTYRLISYVENQGHTGLSFKPTLARKTLQHQLNAELYNVNKLTSTVVVST
LGTLSVAPLIAAIFKIYLQKSQGTHQIDVTSFALPIIFWYPEEYKTPLVYFSLYILQVIY
IYLFAGYMYCGLTSGFMAFKITIYDLKFLCLTVEEWDENSLKKYFSDHDDEEFGKMRSAI
KEDPYDGEYKTGSKSLSKEVNDFPEYDLSLLKAGIVDVIKFHNIICSRASGMNKDFEMMY
TILNNTICFQICVCLYSSAKMDDLILKIENFLIIIPLAGMLFLYCFYAQQLLNEGERFRR
TLWESNFIDKPKWFKSSMLIIMIRIAKELEIKPFGFYVLNLRTFSMVMKAAYSYYSMLNS
LKKRI
>SfurOR13
MSKNEEEKEFENVINGLERANIKLYQFSLLVFYPSGPWKYFNYIVLFFFEIFIIYIMITG
VITIMLLYNDIFAVFEVANAFLIALAVFINLADFHFIRKKRVLWYKLFSYCKDEFYEYDC
ESVKKEMARLRSLCRTYMKWFFMITVPGLIMNIIFIFFVIPITRYMIGKEQPTNDTRFNV
FLPLLWWSTFNTRTITGFIMQYLLMAFVGFYILEIFLVYISTVFYFIMELIIQLKILCFA
LNKHKERIEIFKDGMRRKRQERNDLLATIGKADEDAITTEVLIEPILHHLKIREFFNMFQ
EFSSFFFGLVLSFNMLVIVALSVILTKMDTLFSVDGIKFLTIFIFEILNIFCFTYCGSML
AEESNNVKKSLYSVDWYGFNSNQKKILNIFQIQSASNFLLSASGIYDIDLNIFLQVMQTA
YSVYNVFSTIKV
>SfurOR14
MGLMQEGVLTKPYGIHILLGGLYVNTKRAYWLPCIIYSTLVVLDILFGIFSVAYTVLEIW
SDDVALSLKQFNYVLLRLLTLGLVMTGLFHTEKMDELFPMLVRFQYTTPLNREQEAVIDK
YDKQFWKVFNYFKYLMYIVNTVWIAILPLVEITLNEVILHDRNTTRKFGQYIPVLAWHPY
DPDVFISFLIQYILQVFFTFLLCHTAVGITASYIDVAIFLISSFKVLKISLQDIKQRALF
LYCASTNRSVPLERLDESVTEDPVYQRHLVGCLREDLNHQLLLRRIQRGMLKIYGPILLF
LLLIGGVFICTKAITLLEVNKLNDAHLFRNLFRMIQSVLSFTSYIFVFCWYGEEITQAGA

EVFDALYFCPWSDSPSKLKKAIHTMMICNLKASQMSVAGLKTLNLKTFGEVVSAAYSYFN
IVRSMK
>SfurOR15
MYIGASVPFLVYLVFEVNLQYMILERSILNLELRAEELYKKSVDLHSEIIVKNSLLYEDC
VRECLRENIHHHLAIARLYNLYQDLTTTIYGIVIGLSMSVLATLALVLTRTKLFSFETMK
FTSLFVIELTMVFGYCFMGTSLTEASSKVSNALYNCSWFNFPANQKKTLLIFQLCTSETI
VLKGNGLFEINLQLFVQIVRTTYSIFNIFSSTDY
>SfurOR16
MTMEIIERVRSFTKFLGDRRNRLGSRLFILLNLVIQADQLFSMYIDWNVFVKRLLAFKEF
NSALFAIFVCLSVDDIPSLCNMTENFLKNDKITQEFNIDTNITSKFARNSTGSRQKWDIT
SKYTDDSRQNSKMIVECSNNMLYSHQNSNPTPKYTKHMTNLHLESDITSKNRTNLMWTDQ
KSVTISKCIENMASIDRKLKRILVTLISFASGLPIIFACLITIFKDRSRIDIDRMPFIVH
MYYPEGYKTLKVYFVSLFLSLVWYAAMLSYVYCASAAFALASISLHTRMRLICQSLFEVG
NFGVNETADCNDVVAISQLKQIIREHQEVFNDFLELNRRSKIILSAFINILAIQMCCYIF
FIIELENVSAKIKYVATLFLVFSLMFYFSTVGQRLTDESGNVELALWECQWLDKSTEFRK
CILMMMTRASRKVFLRPYNLYVVDFRCIANIVNATYSYFNLISNSK
>SfurOR17
MILKKVTDSYHYYSSNILLRTIMFALLLNQSLNVLLAVINYWNDMIERFMGLKDVVLFFC
LSACAVDISFRFERCSALLAVIEDKFVGEEHNNQWTTSDTVALYRQQLTNLDRRIWKVIT
VTIFNMNTWPLLAIALLRVGGVKVAHPPLPCAYYLPQTMTSHPVASYVIMFLLQTCCLWI
FAVVGHVILSSVTVATQKSVLDFELFCELVDKFDRCAFGYEEKSEKIFDSYTYGHEKKRE
EIIDEEGMKTETGEKERKRSWRTGEIEREDSWRMYRVESERVLREQTRMLVAYHQKLYNT
MKILGENAGFGTTVSNTNIGLINCIYLYIFVKTDMVVIRIFTLCYVIFSFFLVFFYCHCG
QNIINRNEDVRRRLSEVSWMNRPKWFQRALILMITRSNGDVHLKPYGLYVLNHDTFTKIL
NVSYTSSNVLKALS
>SfurOR18
MLLLNLINYLKSSNTEKYECHINIMKVLFFMLGLWKSPDSREPNYKIVIAIAANLTVSCI
FLLFALHHSVVNVDIEGMTEALMFFILFARIILSYFWLVKRISSINRLIEAAERNYDDFE
ERSIAHKTGLKSECDSWNLLFFKSLIWMFCILQLLYAYATGFYMPHSTEKKYMFPIRSPW
KFEESGNLCYYLAISNQFASSALGCALGTLDVTFSVMSINILSAEVDILGEAWKQTTTYN
QLRRNVRQHVLLLSLMNDVQSILSFIVLDMFVTCEFMLCLCSFQIYQIYSTTSFVSLMKN
ICMLAALIVHEFVYFFFGQRLVSKTMDLHRSVYFCEWYNETKSFKSSMNIVHIRMNKPFT
ILAGKIVPVTLASFITCLKAAFSYFNLLRALSHT
>SfurOR19
MGGAGKIKESREKLVRYLKLTNLYPKENATFFGKFIYAFVNYMFLQTIFAIIQKWSTWSF
NSKVSAIESLNNNMGTLGLVNDIFPFINHIPLSVAMIDGKYFLNGNGRKKKDYLKRVQLI
DEMEEKTVKLVDRLEMILICFYTGYMLAPAFGIILHVMKSQSLNQVILPFMFFISHESAA
RSPPVFSSLQTLLPVFLVESFYIYIQLICTVILHTRFILTVSHVSIEIDLFCMDLEEFCL
ENFECVGDGSNNGKDFAHQKKKLEMIVRNLVIHHQLIFKKVQELNNSLVSSLIYGNSFLT
IQLAILIFCLQQHPDFLQRIRYAIMMIPLLFMLIAYSEYGQRIENQGNKMRLALHNFPWQ
AKPKRVQSTLKILMIRSNMSPQISSVYNFFKQNRQNMSKLMKLAYSYFNLLERIST
>SfurOR20
MENIASKPHKGYKNLLKNDREPSKTHTRSYKKLIYLMSILMCTSGLWTSPKNRTINLPVI
LLIFVVYLFLICGVIASYFDHETISDTIDSSVIVVLILLDLGQYSALIAKKKHLASLIEY

VEQVSEITATGNEYRNLVTSTCEKSYARQITITDNDYHEYFGSKVTVTSDNKYNSKITDT
DNGYQEPVVTDLDDMEAWDNNVKERASRVTATDNCDEYGAATREKGEFWTLFVTAANIVG
VAMTLSAYLFKTKFYRFQERRLLFPLKLPISNQHPLIFTPLLLIQVLGGFISCQFRLFAS
AMINGPLFMLIAEFQILRRDFEQQLLAPSNGRTKNYQQAVGSMQYPGRNFGGTANFDLDL
KRLVKRHNKLVSLCKNIEEVMSKLILILYIICELLLCLQSFKLYKDWSRLSAEDVATGMT
MLLQTVQHLFFYFYASQNLMSQTQDLHTSIYCSQWYKADCKYQKSLSIVLSRSVRPVILT
AGKVLPVTFRSFLKGLQAAFSYFNLLRALTHS
>SfurOR21
MIFYNPFTPSPAISFSTFTQYLLILPVELWYIHLSFTLAFVLNESSFLAVDNVVREMKLF
QMNLHELNKNADAIEKQANATEDYNRLKVIVRQISIHHQLIYKKVEMLNKGFDFQVMYNN
TFICFHLCLAIFCMVKVDLLYKIKYGVSIFAITIISFMYSENGQRLENEGENLRLALYSC
SWIGKPAWFCRSLLILVTQNNRTPKLETFKIFTLNRNNLKVVMQAAYSYFSVLIRFSR
>SfurOR22
MVLLLSEVGRAPEKLQTILGIVKYGVKYSINMKLSEKQKLIIKQANKNDLLMCFCFIGTV
TCYAITTGIIPAISGFIKISTGDLDDDILEKEIPMPVAGSWLPFSIKNLSTYTLLAAFQQ
FAISLHLFIYMGWFAMTSSAMMNISSALKLLACFVDEMDERLGNIQEGKPLDNYIKFMVD
YHNAIYKTIKDYDSATAVMMLLLYAVCVVEQCVSFFCIYEVNDRGLQITFLGVLIVNILI
LGSFTFFGQFIIDEGEKLRLSLNHSNWINKCDSYKRSLLIIMANTQKDVALKPAGLYVLD
RHTIMLIANASYSYLSLMRNFKK
>SfurOR23
MESSNKPANTASRKYTTLTLPPKIVSGIYLILTILLQLNLMLSVHSGWNDFIQRLITIKD
MILTCFCYMSFARVIGRDTTLLCEYVEEHISSVNIGKNRIEGQNLILLNRERKLETTHKI
VKRYYYLLVGICGLLPYVQSLKVLIRADYLERKPKVAFLLNVHYPEEYRSFFMDLIVQTF
LLVWYCVFIYYWISDLKAMSLSFQCITTEMELLRNSIDEMDTIIENHHLAINKQKNMDSD
LFNKIILHSYLSGIVKSHQNICSRMNFFNINLKNLVVFLMNVYALQLCLFLIFVIELPDI
FSKVRYLLRYSIILSLVFFVSVDGEKVTSEGEKLRRALWMCSWTDKPNWLKMSLLTMMTR
ATVDLKIQPFTLYSLDLTCFTQIVKGTYSYFNIYKSLKK
>SfurOR24
MGEIKQRLTEKNPPLEFGDKKTRKLPEEQITEESLLLAFGDKRINGKFGLLFFALTWTLQ
INLIFCLTYEWPDFLNRILTMKEILMSCFATSGYFVQNHLLILENDVIKRYLKDVKYQNT
SRERNRIIGETNNFIKWFSVVSIRAFKIMFIFGGCLPLLQVIIFLTKSYLTGSELKQVPF
VVFVYVPERFRTIHGYLIAQILAFSWYGLVSYMWGVSYKVYLTSIRCVETEMQLLCQSLE
EIGMMSGSGNESILVFKSDTKKVQGNEEDKLRQFFSSIIQHHQHILKLTSLLSKHLRLVI
VTFINIYGLQTCLYMIFLIKMKETGLRMKYAFIYCIILVIMYSCTKSGQDIKNKGEKIRM
SFYECAWVNKPQWMKRSLLVMMSKSVEPIELKPFGLYVLDLNCMANILKATYTYFNIANE
FLK
>SfurOR25
MVRAGKLSTEEESLLEIFSDKRISRKMTVLFFALTWSVQINLIFCLVYEWANLLNRILVM
KEILLSLFAINVYFIRNDMLVLENKLIMRYIKNMNQHNTSKERDRLITEQSQFMKWFGGV
ASVRVFKFIFIFGACLPMLQVSIALARAYFTGKLEKVPSVIFVYVPQVFRTIPGYLIIHL
LCFTWSSFITAICAVIYKVYLTSMKCVETEMDLLCQSMFEIGPMLANDYELNTKEEQRSE
KKELRHLFSVIIQHHQHILRSIKLLSKHLKLMIITLLNVYGLQTCLYIILTLKMEQVGLR
IKNASLYMMSMLIIYTCTKTGQELKDKGDKLRMALYDCSWIDKPVWFKRSLLVMMSRAIQ
ATEFRPFGLYSFDMNCMANVLKATYTYFNAANEFLK

>SfurOR26
MDDKQGIKEEKFENIVSGLERAGIRLYKFSLLVFYPSGFWKYYTIIGFILAEILGAYFLI
VGAVAIIQQMDDIFILFESVHAYLIMIAILTNLNDFYIVRPRVRNCWYKLLVLCKEDFFE
YECEYVKREAARLRMLSKMYMKWFFNIAINGIFVTLIMVMVVIPLVRLYLNEEQKTDNTS
YNVYLPLSVWSPFNSRTVAGYAFNYSLLFITACHLLIIMLVYISTIFYFFMELIIQLRIL
CFALEKHDQRLKILRENATKQQTFNSTSNKMISRAGDQFENYCITKSLEDPILHYLKIRE
FFNVFQEACSFFFGLMLSANMLVIVAMSLLLTRIDSILTVDGLKFCMILSIEIVNIFIYT
YSGSMLVEESHQVQKYLYSIEWYGFTSNQKKTLNIFQNQSGSKFLLMAAGLYNIDLDTFI
QVMQTAYSVFNVFNNMKS
>SfurOR27
MESNAFDVDIHFRNKIRLLRYVSSWWTNPSKLWLNLLEKSLSAIRILLMMLASVCQLIQL
TKMSNLKGASEIVEVMTLTISACYKSTFFLYYQKDYAALLNLMRGCFCENPLFFRKTQKK
YTNMSKFLCYTDAITYLYFTTATIFILFGIIATIFVPETQNSKVLPYCKPNPTLIGCDSL
QHNLNSQQLSKSKPNLTLIDCSNVQHNLNPQQLSKRKPNLTSIDCSSKVQSHHNSSLILK
VWLPVDYSWTPFYQIVHLVTSFILFDCCLIYVAQDAFCFTFLYAVCGQFQILEESLMNLD
HLARGRLNVAAKRTYSEQGKDVQESIPHHIDGNVDEKLLHKMFGECIQQHILLLKLLRDY
EKLMGSMIIIDFLHAICSLSFALLELSVAHTLVHAIKLFCFIAICIVHQYMNNYFGELVI
QKQLSISRAVYSLPWEEFPKKIKSSILFMLLRTERPIVINGFKMYLLCYRTFVEFLKAIT
SYYTVLRSVHLEK
>SfurOR29
MELVKMSDVVATFMKHLNSTGLYSPKKPSLKGKITYFILIYVVFEVLFVMIHSWKTWDFN
SRVTALENLNLAIGIFNIATEHMLLHDRKDNQIQLMSSEVYLYDVTKDGVGISMYQEKEI
FVESMEKDSIKLTNIIVTSLRWYFVGYLMSSLCGLLFYLINDDFSSLSLPIVFLNPLTPS
PSISFINFKQYFIILLFELWYTHLSFTLVITMGGFYFLSIDNVTREMKLFHINLKELNLN
LESIEAGVGESGPDRSNFEVLKPICKRIFTHHQLIYKKVRMLNQGLDFIVMYYNPFICFQ
LCLAIFCIVKVDILFKIKYGFAFFTVLVMSFIYCEKGQSLENEGEELRNALYNCNWVGKP
TWFCRSLNILMTQNNQIPKIETYGIVTLNRKNLRGIMQAVYSYFNLLMRFSR
>SfurOR31
MLDLIYTILHGEKIDRNQYNSFLKIMKLLLIVSGLRKQSDPEQLNTILIIINVILLVSGC
ITCLLSCLRFINDLNTFTEALMFFILFARVIGNYYSLGTNLRKINRFLWACETNFDEFEK
KNLKEISNLKQISDHSVLTFVVVMVSLFVSMFVTYTSSFRFFPITSPWDNQIGSSVFLSN
IALLYQIVVCFISSVVSLTDATMGLGSVNIICGETEILGRTLLATDGNPRLKLCVKQHCF
LISLMYNIQSIFSLNIFLIFISCEFMLCLCSFQIYQMHATMTIASLGKNICMLCALLFHE
FVYFFYGQQLVNKTADLHHDIYFSKWYKKPAQFKSSTSIIQYRTRKPFVIMAGKIIPVTL
ASYVACLQAAFSYFNLLKALSHNQE
>SfurOR32
MNDQEMWDPLTLVSKSLAFMGRPIPGKIEIRYVLMTILAIMMFLQSFLFLVMRFSDLKCR
LLAFEDMVVIFYTCGMLIDFLLKPKKGLTMMATIKRKHFNVKEVISVEQKAMLDKLERKA
QSTTKILVITVINFTIGNVILPITIGVITLLTKNKGDSIVLDSGNDTSKWQESLKNEIDN
EFGDYPLPFEGWYPFSIAQLPIYLTLSVIQATYQGVESTIFICWVVLIMCAYLTVEMELK
LLCQSFREIDLRLEKYMMNQEGIIPDGNVDELKNKYLKRYLRNLVEHHQSIIEVVNELNR
ASSFLVFMFNQIITCQVCMGLFASQSQIDDKVFKVKYRILILPVLFSFGMFCYYGQGITN
EDEEMKKAVVHCSWQEKPLWFKKYILLILTRLNKPLEIKPLGLYVLNMRNFSVILNASYS
YYNLLLTAESKNRAA

>SfurOR33
MNEITRFIFCLTILLAVPLVTIRFSLLPVMVLHAVGIFETVSADIAKLKYSCGDNTEEII
IEIIKLHQKALIISKNLVEFFRPIILLKAVFSFWMFAVLMVVITEEPIGSSIFINCAFCM
LCSGYELLLDCWSGEQLARKSEKVGVAAYQCQWDRMLNGARRNLVLLTLRAQRPIQLDVR
PYLIPMSLKTYVQIMKASFSCYTLLLNIKAEKH
>SfurOR35
MYQPFNILKILMSFMCIHPPKFCTTSELKLSMYNGLTGLAVAIEFLSYIFYFPSTFFMYK
SGSELTPFQAFIALYGISMMMQIFMMITTRSELMDIIKSIHDKYAADGAHAEVENEQSRK
RSLWLFSAITVLQVPTFLGGIINSEYIGNNEDSIKANIHFGRKNPERLIAYLPWLPYDYT
ISPYFEVTKTLMYIALTMGCISMMVRYSLLPMLTFYINGQFDMIAKRFESLKATDEKPGS
NGRKLIEEEILNEVIRSHQQALGLSKRVIVYFRPLIVTKTIFYFGILADLLYAVTEQTAG
TAQQINCCFCLMSCGYELLLDCWCGEYIARKSQLVGLAAYKSEWHNMSFNVRKSLAIVIN
RAQKPEQLNGRTGFIPMSLETYIQIFKASYSYYTVLQNLNKADQQVQI
>SfurOR36
MLWWQISKADWASVKRGLEKSGMTFVLTSGLLVSPEQPLLAFNIFIIVLSAFTMTFFVVI
GLVSSYLARNDLLAFVELAHANTFSLALWILVITHYSMKIPLAYEAMKMIDRGIFEYDTE
LNQNEVDKIKNTAKVYTNLFKKWYRMSLVILFIILAFVAPLLISIYGGEERKKIKQLNYN
LPVPIWFPFDTENLLGFSCAYFLFLVQLGLVFSYICAAMPLLFFGIMEVVAQLKILKLSI
NSLEMRVLEKCQKIGKDLSKAGVETLEYDPNYERCVQECLKENIRHHAEILRFFNLYQAL
ASTIYGVIIGVSMLILASLTLVLTKTKLLSFETMKFSTFFVVELVVVFGYCLMGTFITEI
SKEIPRALYDYEWFNAHDKNKKILKTFHFISSVNIVLKGNGIFVIDLKLFVQIVQTTYSI
FNIFTSMEK
>SfurOR39
MEKIISKIFMYLQKLGCLPPGRSSTKGRMIFLISIYFILQTNVIIVQKWSIFEQRITAME
NINIFYAFFFAITDYYLFPEHLKTMMEVINFPHFKYDKRLHQPELAAQCSALMMETRKTW
SKTGIILEIAMKCFFFSSNILPVIGFIQYIIGYETEKDLTLPFVSYITVDKENLISYIII
LIIEMIYIYYAFILAILLYLMTIASVHNVILEMRLFCMCLEGFNEHYLKKINGIQCENDV
NSKRSGGDERKFDEILILKLISRELSEHHQLIYRKIYMLKKGFKFQLCYGNGVICFQLCF
ATFCFLKDDLMFKLKYGAVTLLVTLLLLIFSESGQEIQRQDEKLHIALYECAWYNKPLWF
QNCFKIMMTRNSIPIRMEVYRIFTLNRNNVTVVLKSVYSYFNFLRQFSGDL
>SfurOR41
MIGLEDDSQHFVRTFVVVSFCYTCLPVLNAVYARLTMNSSADFKDLPQVSYLHYPLSAGQ
PVTLTHYLSGALFLYAGNLSAALFSFCSLYASYLALRCVADAFQQLAALIGAWNSSAATL
PDNAFREIVVFHQQICREARLLNKGIETATINLLQGCVVQLCLGLFSILEGGDKIKYGGF
TCFVLLMLVGFSSFGQILEDRIERVRLCIWESDWINRPPFIRKALFTMMIGATARLEIRP
YGLQQLNMRSFAGVLKTVYSLFNVINQSR
>SfurOR42
MRGFSNPEDCFRWVCGFLGRPYPAGKIHFRFYIIVILYIMNFLQLLLGMRPRWNNFQARY
QSIEDLIVLAFMTIIVCEMGWSFNKIVVMVEIAKSNLYPASQPLSDRQKSIIEKMNDEIL
GIIKTFAFIVAGFSLINGFIPFLHGIFFVSRKDINNVSQEQIPMPVECWIPYNIEDVITY
IIFVFPQVIMFAMCSFVGFIWFTPFIISLFHLMKEIDILCHSLNDMDYWLEEKTGSDYDF
HLQKYFKGVIEHHQNICRTVQQLNEALSVFLFLFNCVCCAQICVSLYSCFETSDSVSQMK
YILLLGPVFSCFYFFCWSGQELINRNDQLEKVIANCSWMNKPKWFRSSLRIMILRSSKPL
QIKPFGLYTLNFNNIMMVCRAAYSFFNFMHKVQMKSVY

>SfurOR45
MENDIEIKLKSKLKKISEICKNYIGFQRDGISFILFYGAAINIFAFCALSGVKAVQLFSS
GNDETLISNECLNAVVVFFSLLSAEIKLILYFKNRRKAYKLYSVICKTLPLNSNDMEREG
EETIHIAVMILIWSSAAATLFALLNSNVLSFNRGETILLFQIYLPKSIERNAFTFYAVYG
LQLLMCTHVVAINGSTLASFAHASNVLCVQNKVLGIAICNLTLDDGGRITDVKLTFGHRR
QEKMREYVEFHLHLINIVSQLESVFGGLIFWEFVGSQCLSCLLTYLSIVEANKGRIFSSI
RTIFMIPALLITSFIYFFLGSKISEASEELRKLLYSSYWYDQPLWFQKCLLIMMKRMQKV
LTFNSVHIIPVTLNSFLRALQAGFSYFNFLNAMTGRYK
>SfurOR47
MDLYEDTDENWESTYRGLIKTGYLSFIKVAILIAPKGRDYHLSGLILLGYSTLIQWILLY
CGFISLFKASNDLILFIEIAHYYSILVAIHILFVEHYIFKMSRNRKIFQVMDIDIYDYGT
DFDRNEMENSRLKTGYYMSYIKVGFIVCLAMTGCALSILPFVQIARGVEKMRYDILVILP
VPFWFPFNTETSVVGFGFAYFLELNMIIFTIFYLGTYIPFIIHMMVEIVSHFMILNHSFL
TIEERTSAMYCTLKPLDKNSPSTSQVMELAEDSLYDRCYTECLRRNILHHKAILGFVELF
QDVHSTLFGVLLGANMLVIAAITIVITKVEFSMNTMKFYLMMLIELTNVFMYCFLGTLIT
EEAKSVGIAIYSTPWISSSGKNKKIVQFVQMRISKQVVLKGNGVFSLSLQLYIQIMQTSY
SIFNLFNSVDS
>SfurOR48
MDTLNSVRRTLTLAGHRSDGSVAFRTHLFTTFLVSYAMDLTYSMFLSDTGDIKKILAVKD
LVSIVMALVLIPASPGVYKLCKVAEAQMQRNILQPIVPEVADIRNQLDLEHNKTGKKIYK
TLVLIIIVYVSLPVLQSVIVIALHKIPQLSTLGMMILGNSENQTGIKTLNVEEDLALVFY
FYYPPGLTTIWFYFILSLSHAVWCIWICLFLYSIFNSAMMSIKNTISEMKVLGVAFDRLD
ELTGNGSSVYNDDNHNRTDKQLNETLLKDYFRLVVIQHQLVCRDLRSIDQGLKTVIVALD
VAFGLHMCLAIYCIIKMESFSLKIDYTVLLFWLMTIVYNYSAIGQQITNEGDDLTMKIYD
WAWVDKPQCIRKALLLVLTRASRKMELKPYGIYVLNLTSFKNVMNATYSLFNLLKKIA
>SfurOR50
MSMANKKIFSKIIQVAEWLRCDELKLAAPITLVMINSFIVVSGMLAAIISNWDVLEIRFL
AIKDIVIVISLTLVALEYAFSNKKIMSLIQIIDGKFLYQRLHGSISSHEFKFGENNGNIE
IRQVIQKAWHSIEELDRGIMNSVRIVTLFLDLTPFLINILIILNTGFDKITIKDLRLPFF
FLYPKNYDKISVYLLMYAIDSLFIYFIYIFSYCAFATVTIGAQKTATDLEIFRMCLKCFT
NDNSDLIWTHKDEKQIRRGKIFGREMDETVNNDDIDYLVIVIKYHQMICRQVRVLGDNGG
LVTTLANFCIGLQTSVCLFLFIETNNVIMKTACASMLFFNGLLLFFYCSSGQKIIDENEL
LRKQLAEIVWWNRPRKFQTSLLLIMTRANHDLYVKPYGIYVLNYNVFRNVLNASYSYLNM
IRALK
>SfurOR51
MRQVAPEVFEESKDYAFNYPDSIKLGSHFCKGALALLGRPRLAGRMTLLFLAYSAAVIIC
CLQMIVFLIVEWNSYELRFKALENLNLTILCFSILVEFGIYPQKTQILMKIIDAPIFEYQ
MPLSENMKTVKLEACKRDKSVQKIFGGLMLIYYSMTFVFPVIYAIFALLFFSNIKVDELP
FIYVFWLPVPMDNFILYFVQLLLQSLHGIVLCSGYTGWFVLFISATVHTQHEVKILGLYL
REMDERLLSKNFELKSLTERKEKLENDQNRRLKLYLNQLCKHHSAIIRTIKTFNENGSSM
QYIYNQMVVFEICIHMFLYLRNEGVERHKNLFSSLLGVLLISNVGYFGQGLIDEGDRLIR
ELSHCSWVDKPTWFKKSLKLMMMTAIHRPLQIKPVGLFVISLENVSRVLQAAYSYFNVMR
RLKDK
>SfurOR52

MSKVVNTFDVRCFRVIGLWQLISSTKGYSNWSKSNSDHQNNTSKNITDNSYRSKNSCDKI
FGNNNSKNISDNIFKSNSPGYFYLLNVTVMTVFVPYVLMEFFGLMTSHYDFVTLTEKLSV
NITLLESAVKFLYCYMRRRQLADLLRVFEENFLLCMQQNRDTNLKILENYAKNLTGNINL
FIIIIYATVGLWNSLPILSCLQGTGCSTLQIMPSWYPISIDQAPIKQLVYLLEFIIMCYC
ASLLYSVNCLILAFARMISSQVEILKLSMTNLAENAARAVYGELVKNGNFTGSEILERRY
QEKLDDLQKDCLIDYQRILSQAALMEETFSPLIIFQLAVSTITLCLVLISLTTKTAADST
ISLTLGCKFSMYLIFGLMELFVYSWCGHIIQDQTGSLHGSLYEMAWLSNSLNFKKNVLQA
MTRALRPCRLTAGKFRPVDFKTFAGVLKMSYSLFTFLYGSGFKK
>SfurOR54
MLADNQSENHKSPNETSKHRFITLQLKMLRFLCIWPLHSKNQIYVLLHKIFAFFRITIMI
LVSIGQFIQLFFMPNLGLLSSVIDVATLTTSAAFKWVFTMLHVKEFDKLNNVIFDKFLDA
PLVESKKSKMTMNSLLKYKDLLCVGYCVSGTLIMTLWAFTPLLNSFIQHDKILDRHDNDS
DRFLSPYHDLKMPINSWLPFSPTWSPLYQIVFFVEYCTFIESAMVYGFQDSYFFTMLYCV
SCQMDILCETMRNIKINLERMSVKGKRSGNFENLDNSQRRIEIEEPDDHRGTSEYATIDK
IQYDEVELHNILKQCIEHHTVLLKFLRDYEKLSQTMIIVDFLHAIVSLSFALLEITVSQN
IIEYTKMLLFLVICIWHQFLNNHFGELIIQKQMSIADAAYDLPWVERSPQFKTSMKIMIM
RSQQPVILNGLKMYFLCYKTFMEFTKALISYFMVLRTLQEGK
>SfurOR55
MYDSLRRYQVVLLLVGLWPNDFNSTWKKILYEIYTWFFMLHFVMVAIMRLLQAYYSQFDL
ELFTKASFECGFATLLMLEIVTMLILRYKIRDVVIAMRKGFVLESDYAKKIMEKCDSHED
LLFKALFVVELPAVLAEIVHCLLLPARPADYRFNTYWNKTHPEKRMPFPLVMPCDDTISP
CLEIEAFFVGIEIFQGISLMFFSYTLVPSFITQVCGQFKILVGRIESISSVDGNNNAQGN
LENEIHFQATQSQKDREVKEVIKMHDSLLKISQTVVALMRPFIFLKTFFSFGMLSIGLFT
TLTVGIGSPSFFTAGGFLVVATLELFLVCIYSENLSTSSLAVYNAAYNVSWPDMKESTKK
SLVLLMLRAQRPLQLNVKSNFVPLDLNTFIQVIQTCYSFFTVLRETLGKQNNSDLKTE
>SfurOR57
VPFWFPFNTYTLTGFSVAFLLITTEICLVLFYVGIGLSFVVFIALEVAAQLRILHDSIVN
IEPRAIERYRCWRELGKHNDMLGDDPLFQRCVRECLKENIQHHIEIIRFFKIFQSVISNM
LGVSIVDAMSLLACLSLVLTKVEPFSIEAVKFTFFFILELVFAFGFCFMGTSITEASKTI
PEAIYRSPWFRLLRGEEKRTLLIFQMNAARNFVLKGSGMFAIDLQLFVQ
>SfurOR59
MIVLIYSYLTYLTFIFTSFYTVFSGDSNLTMFEVIDVFTGAVPGFIAFAMLCCFCWRVDD
VRCLKQLFDKRLLSFGTNDDKEIVRENIKRSNMFIFIYMGPVYSNLIFWLIRPLFSNDKS
IFNTINNGTEIIYKIVDCHYPFDYTVTPNYYYVCLYEIVGVYQLITLMLVFDTLFGSMIL
VFCGQLKVIKFNIDKLKMDKLQISQQNGEQRVFNVTMEENLKCIIKNHQKLISIFQVMKH
VFHPMIVVKMGGCCFAVCLMLFKVSKARSFNASQIIRDTAYLITHITEMFMFPWFGHKLK
QT
>SfurOR61
MVLWQIPKEDWLRIKRGLEKSGIRFVIASGLLVSPEQPWLTINLLMMLLAIIFMSFFVYV
GGVSMYYARNDLTTFVEIAHAYTFVLAFWVLLTVHFVFKLPVLHELLQTVDGEIFEYKNK
CCEEKEAKVKSQNRFYHLLFQTVLDSAVLVAVFILGFLAPAMIKLFEDENRRKVKEINYN
FPVPLWFPFRTDNLLGFCFAYFLLMVEILLIGIYLLAAIPFLVFAALEVNTQYRILKNSI
LNLEPRALEKYNGMCRVSNNQLEILRQDSFFKKCVQECIKENILHHIEILRFFSLYQDLS
STIFGVIIGVSMIILASLSLVLTKTELLSFDTLKFAFFFIVELVVVFGYCLMGTFLTNS

>SfurOR62
MEIIEEMRSTNLNYTRTGLLLLQGLSMAFLLVPLHGFISYMIGAIEFQELKFAIMVYEPF
TGSSSPANFAQFIAVTIVQALYIYLFYMLSSDSFLIESLSNCTLKTEIKLFLNNLEEFDA
IFCARKWDDKDGILSARLRKLKIFEKFGRIAAHHQIIFRETRIYQKTMAYLTVYFNLGVC
GGMCISLFLVEQGDFFFKIKYAVAACCLGLALALYSQEGQDIEDGFEEMKDALYRISWHN
LSVSLRKSLAILLTRHCLPPKVTGSYGIFVINRKNLSMI

Supplementary Table 6.

Primer name	Sequence (5'-3')
GAPDH-F	GACAGGATGCAGAAGGAA
GAPDH-R	GTTGGAAGGTGGAGAGAG
β-actin-F	ATGGCTTACCTCCTGAAG
β-actin-R	TCTCAATAGTGGTGAAGACA
AlucOrco-sF	GGTCAACTCCCATCCGCTT
AlucOrco-sR	GTGCCTCCATTCACCACTC
AlucOR1-sF	GGCGTTAATCTCAATCTACC
AlucOR1-sR	ATGAAGAGCAGCGTGATA
AlucOR2-sF	ATTGTTCACTTCGCTCGCC
AlucOR2-sR	TTGACCTCCTTCGGTTGCT
AlucOR3-sF	TTTGAGTGGAACAGCACACC
AlucOR3-sR	CCATCTATCATTGAAGACAACATC
AlucOR4-sF	GAAAATTAGAAACGAAGAAACCA
AlucOR4-sR	AGCACCACCGAAAATGAAG
AlucOR5-sF	AAGTACTAGGAGACTCAGAGGG
AlucOR5-sR	CGAAAAGAAAGGCAAAAACGAA
AlucOR6-sF	ATGATATGTCTGAGCATTGG
AlucOR6-sR	AACCGATGTCTGACTCTT
AlucOR7-sF	ATCACGCAATAATCATCAAGT
AlucOR7-sR	CGACATTCTCACAATAGGATT
AlucOR8-sF	CATACAGTTGCGATTGGTA
AlucOR8-sR	TGTGAGTGTTGTGAGATTG
AlucOR9-sF	ATCATCAAGAACTCGTCCA
AlucOR9-sR	TTCGCAAGCCATAATCATC
AlucOR10-sF	AAAACTTCTCCTGCCACCA
AlucOR10-sR	TTGCACCTATAAGACCCACATA
AlucOR11-sF	CCGCTTGGTATTCTTGTG
AlucOR11-sR	GTTGTTGTGGACTTGTCAA
AlucOR12-sF	TTCACCATCAGAAGGCGTC
AlucOR12-sR	TCCATGTTCATAGGGGTAACTC
AlucOR13-sF	CATGTTTCTGCCGATTCCC
AlucOR13-sR	CCCTTTTGTAGAGCCCTCACT
AlucOR14-sF	TGTTTATCACGGCATTAAGGA
AlucOR14-sR	CAGCACTGGAAGCAAAGTTT

AlucOR15-sF	TGATGCCCACATCACTTGC
AlucOR15-sR	GATCGGCCTTGAACACAGT
AlucOR16-sF	AAGGAATGGAGAGACGAAGC
AlucOR16-sR	GGGAGGTCAAAGTGAGCGA
AlucOR17-sF	TCCAGCAAAAATTCCCAGG
AlucOR17-sR	ACCAAATCAGCAAACAGCG
AlucOR18-sF	ACTGGTAGCGGAGGTTTTATC
AlucOR18-sR	GCAACGGCTTTTTGATGTT
AlucOR19-sF	TGGATTCTTCACTCTTGGAT
AlucOR19-sR	GTATAATTTGGGTAACGCAGA
AlucOR20-sF	TGGTCACCGACATCCTCCT
AlucOR20-sR	CCTTCGTGCCTAACTGTTCATA
AlucOR21-sF	TGGTTACTGTGGGCATCTTG
AlucOR21-sR	CTGTGTGGCTCTCGTATCTATTT
AlucOR22-sF	GGGATTTGCGGAGGTTGT
AlucOR22-sR	CGAGTGCTTTATTCTTGTCTTGTT
AlucOR23-sF	ACGGCAGAGGGAAAAAAGA
AlucOR23-sR	TCGCCATAGTAACAAAACATGA
AlucOR24-sF	GACGAGCAACTGGAAAAAGA
AlucOR24-sR	AGACCTGACTGGACTGGAGAG
AlucOR25-sF	ATGTCGTTTTTGTTCAAGTTTTT
AlucOR25-sR	GGATTCCTTCGTGGGTAGG
AlucOR26-sF	TGTCGTTGTGTGCCCTCTAA
AlucOR26-sR	GAACCGATGTCTGGCTCTTT
AlucOR27-sF	GAGATACCATACAACAACACGC
AlucOR27-sR	TATAGTTCAAAATGATCAGCCG
AlucOR28-sF	GGAAATGGAGATGTGGTCG
AlucOR28-sR	GGTGAGAGTTATGAGGTTGAGTG
AlucOR29-sF	CTGGCATGAAGTACAAGAAG
AlucOR29-sR	TCACGAACGAGTAGAATGT
AlucOR30-sF	TATCGGAAAAGAGGAGCACA
AlucOR30-sR	AAACAGCCATTACCAAATCG
AlucOR31-sF	GATAGGAATGAGAAAAAGGTAGAAA
AlucOR31-sR	AGAAAAACTCGGACGAAGATG
AlucOR32-sF	CCTTTTACTCTTCTACTTGATTGG
AlucOR32-sR	TTACTTCTGTTTGCTGGCCT
AlucOR33-sF	ATGAGAGGGATTTGGTTTTTC
AlucOR33-sR	CAGCATGTTCAGCACGTTG
AlucOR34-sF	CGCCCACAACGACATACCC
AlucOR34-sR	ATGACCTTCCCAAAGCCTC
AlucOR35-sF	AACAGCCGTCCTAGAGGAAA
AlucOR35-sR	CGCAAGCAATGATGGTAAAT
AlucOR36-sF	TCTTGTTCGTGGTCATCGTAG
AlucOR36-sR	TGATCATCATGTTGAGGTCTTTC

AlucOR37-sF	GGCACCGTAATGAGCAAGA
AlucOR37-sR	CAGAAGATGAAACAAAGAAATAGAAG
AlucOR38-sF	AACATTTAGTCCGCTTGC
AlucOR38-sR	AACGATAGAGCCTAGTTCAA
AlucOR39-sF	TACAGCGGGTTTTTTAGTTTTG
AlucOR39-sR	CGGTGTGCTTATTGTTGTTATTG
AlucOR40-sF	TCCTTACCCCACAGACACAC
AlucOR40-sR	ATGGTCAACGCTGATCCAG
AlucOR41-sF	GTGGAGGAGAGGAGGAAAAA
AlucOR41-sR	ATCCCGCAGTGCCATTAG
AlucOR42-sF	AGGTATGCTTTGCTTCGCC
AlucOR42-sR	CATTTGTGCTGCCTCTGTCA
AlucOR43-sF	CGACGGAAAGGTTGATGTG
AlucOR43-sR	TGATGATTGCTATGAATGTTGTAA
AlucOR44-sF	ATCGACGTGGGACAAGAGTA
AlucOR44-sR	GAAGGTGGGCAGGTAGAAAG
AlucOR45-sF	CAGCAGGTTGAGAGTATTGTGA
AlucOR45-sR	GAGGCTTTTTCTGGCGTT
AlucOR46-sF	CGCTTTCATCTGTTTTTTCAC
AlucOR46-sR	CAATGCCCCTGGTTTCCA
AlucOR47-sF	CTGGACCATCTTGGGAGG
AlucOR47-sR	GGTGTAGGAGGTATTGAGCATAC
AlucOR48-sF	TCTGAATCGGTTGCTGTA
AlucOR48-sR	AATATGATGATGAAGTTCCAAGT
AlucOR49-sF	AGCAGGAGGCAATGGAGC
AlucOR49-sR	CCGTAGTAAAATGAAGACAGGTAGTA
AlucOR50-sF	GCGGAATAGTAAGAGAGAAACAC
AlucOR50-sR	ACCAACCCAGCTCCAAGA
AlucOR51-sF	CGCTCGTTATCTTTTGGGT
AlucOR51-sR	TGTGAAATAGTTCGTTCGCC
AlucOR52-sF	CAGCCTTGTAAACTCCTGTTACC
AlucOR52-sR	CGCTTCTTGATTCTCTCCTCC
AlucOR53-sF	CATGAACGACAAGGTCTACGA
AlucOR53-sR	CTCCGCCACAATAAGCAAC
AlucOR54-sF	GAGCAGTAGACAGTGGATT
AlucOR54-sR	TGGAATAGAAGTGACCGAAT
AlucOR55-sF	GGGCTACAGTTCGCAAAGG
AlucOR55-sR	TGATGGCTGACGCACTCATA
AlucOR56-sF	CTCGGAGACGCTTCTGACTT
AlucOR56-sR	GATTTGTGCTTCTTCTTTTTGTT
AlucOR57-sF	TGTTCGGCTTACTGCTGTATT
AlucOR57-sR	ACCGTTCGCCACTGTCTC
AlucOR58-sF	GCCGAAGAAGAAGATGATG
AlucOR58-sR	CAACATTGCGTGGTGTAA

AlucOR59-sF	AGTCCGTCATCATAGCAAT
AlucOR59-sR	CCAGAAGCATATTCAGGTAAG
AlucOR60-sF	TCCTCTATTTCGCCGTGTC
AlucOR60-sR	TAGTTTGGTGCTTTCGTATCC
AlucOR61-sF	ACCGCTCTTCTGTATGTTGG
AlucOR61-sR	TCTGTGGTACGCAGGCTTC
AlucOR62-sF	GGCGTTCAATTCCACTAC
AlucOR62-sR	TCTCTTCGGCAAGTATCTC
AlucOR63-sF	GTTGCCGTTTTTGTCGTG
AlucOR63-sR	TGTGTCGTAGATGCTCCTGTG
AlucOR64-sF	GACCCATCAGACGATTACTCAC
AlucOR64-sR	CTGCTCATCAAACAAACCATAC
AlucOR65-sF	TTTCGGCTTCTTACTGGCT
AlucOR65-sR	GCTCATTTCGGGTGCTTATC
AlucOR66-sF	TTGACCTCGTGTCTCCTCG
AlucOR66-sR	TGCGTCTGTTCACTCCATTTA
AlucOR67-sF	CTGTGGAGAGAACCGCTGA
AlucOR67-sR	CCCCATGAAGTTGTGGAAG
AlucOR68-sF	CACCGTTCTTTTTCCCTTTA
AlucOR68-sR	TCAGTCGCTTCCCGTCAC
AlucOR69-sF	CACTTTACCGCAATGGCA
AlucOR69-sR	GAGGTTGAAGAACGAGTAGGAC
AlucOR70-sF	GAGTTCCAACAAAACACCAGA
AlucOR70-sR	AGAAACGAAGGACCAAGAGG
AlucOR71-sF	AGTCGCCGTGTCAGGAGT
AlucOR71-sR	CTATGCACAAGATGATGTTTTTATT
AlucOR72-sF	TTGGTGGTGGATGATTGA
AlucOR72-sR	CCGAGTCCTCAGTTACAG
AlucOR73-sF	TTCGTTTTCGTCCACTATCC
AlucOR73-sR	ACAATTCAATGTTTCCAGGC
AlucOR74-sF	CGGGTAAAGATGTACAGGGC
AlucOR74-sR	ACGGAACACGAAGAGCGA
AlucOR75-sF	CACATTGCGAGGACACACA
AlucOR75-sR	CCCCCATCCAGCAGAGAA
AlucOR76-sF	ATGAGTTTCGCAACGCTTT
AlucOR76-sR	TCCGTCACGTCTTGACCG
AlucOR77-sF	AGGGAGCAATAAATGGGGA
AlucOR77-sR	TGTTGAAGAATGAATAAGCCG
AlucOR78-sF	ATAATGCTACGGCGACAA
AlucOR78-sR	TATGACCTGATGTTGGATTCT
AlucOR79-sF	GATCTTTTACCAGCTACCGCA
AlucOR79-sR	CGCCACAGGAACACATTACG
AlucOR80-sF	AGATACCAAGAAAGCCACCAT
AlucOR80-sR	TCCTCCCCAACTATAAACGA

AlucOR81-sF	TAACAGTAACCAAGTTGAGCACC
AlucOR81-sR	AATGTAGGAATACGCAGTAGAGAG
AlucOR82-sF	GTGCTATTGGACTGGGGTTA
AlucOR82-sR	AGGACTAGAGGTTCGGTGATG
AlucOR83-sF	ACATCAGTCAGGCTTCAT
AlucOR83-sR	TCGTATTGAGTTCCAGTGTA
AlucOR84-sF	GTTGGGCTGTTGTTTGTATCTT
AlucOR84-sR	TGGCTCTCTTGTCCGTGTC
AlucOR85-sF	GACCCGTGCTTTTCTATGC
AlucOR85-sR	CCACTTTTCTGCTCCCCA
AlucOR86-sF	TCGTGAGAGTCTATGAGTTG
AlucOR86-sR	AGTTGTGGAATGTAGTCGTA
AlucOR87-sF	AACTGGTTTGGATGGTGGG
AlucOR87-sR	TTGAGACTTTTGCGATGACG
AlucOR88-sF	CCCCTACTTGCAGAAGATGG
AlucOR88-sR	TGTTTACGAGTGATAACCCGAT
AlucOR89-sF	CAATGGGACCCGTCTGTC
AlucOR89-sR	GCAATGGTTTCCTGCATCTAG
AlucOR90-sF	TATATGGGCGTTATTTGCTCC
AlucOR90-sR	CCTATTCTCAAGTCGTCGGTG
AlucOR91-sF	AGGAGGGTTTTCCGATTTT
AlucOR91-sR	ATTGCTGCGGGTTTGTTC
AlucOR92-sF	TCATCAGTCTCTGGTGCTCTTT
AlucOR92-sR	GTTCGGCTCACGTTGGGT
AlucOR93-sF	AAGACATTGGACGACAGAA
AlucOR93-sR	CAGCGAAGAGCATAACATC
AlucOR94-sF	ACGGTCAAGAATGAGAAGGAA
AlucOR94-sR	GTGTCGGACGGAGGGAAA
AlucOR95-sF	GGTTTTGTACTTAGCATTAGTCTGTG
AlucOR95-sR	CCCTTGTGAACGCCTCCC
AlucOR96-sF	GCTGTCCTGCCTCAAAGAG
AlucOR96-sR	TCAAACCAAAAAATCCCATT
AlucOR97-sF	TTTATGTATTGTCCGTGAGTGTG
AlucOR97-sR	CTCCTGTATCTTGTAATGGTGCT
AlucOR98-sF	GCAGATAGCAATGGAACTC
AlucOR98-sR	ATGACGACAACTACCAGAA
AlucOR99-sF	CAAAGAACTCCCGCCTTC
AlucOR99-sR	TGCTTATACCATTCAGTATCATAAAT
AlucOR100-sF	ACAGAGCGACCATCAGAACC
AlucOR100-sR	AACTCACCACGAACGAATAAA
AlucOR101-sF	CGACCAGAACCAACAAACC
AlucOR101-sR	GAAAAGCATATCGTAAAAGTAAACA
AlucOR102-sF	CATCTATGTATGTTTCCCCCG
AlucOR102-sR	GTCAGACTACCCTGTCCCAAT

AlucOR103-sF	GACCGCATCAACGCTCTG
AlucOR103-sR	TGCTTCGTGTAGTTTGTCTCTTC
AlucOR104-sF	GGGGCTTTGTGGCTTGAA
AlucOR104-sR	CGATACCGCCGATAGATTGA
AlucOR105-sF	GGCGGATTACTACCACCATT
AlucOR105-sR	CCTACAGCCCTCATCACACTT
AlucOR106-sF	TTGATGATGGTTTGGCTGAT
AlucOR106-sR	GTTGTGGGATGGAGGAATG
AlucOR107-sF	GTGGAGGGTTGGGACAGG
AlucOR107-sR	GGGTAGAAGATGGGTATGAGAGAT
AlucOR108-sF	GATGAGCGGTATTTCTTGTTT
AlucOR108-sR	ATTACTGCAGTGAGGTTGGAG
AlucOR109-sF	TCGCTCTCGTTCTCGTAATG
AlucOR109-sR	TCAGATAGGTGATGGTGTTGG

Supplementary Table 7.

Primer name	Sequence (5'-3')
β-actin-F	ATGGCTTACCTCCTGAAG
β-actin-R	TCTCAATAGTGGTGAAGACA
AlucOrco-dF	CGAATACCACGAGGAGAA
AlucOrco-dR	GGAAGCAGACTAGGAAGG
AlucOR1-dF	GGCGTTAATCTCAATCTACC
AlucOR1-dR	ATGAAGAGCAGCGTGATA
AlucOR2-dF	ACACCATCTCGCTATTCTT
AlucOR2-dR	TCCACCATAACTGTCACAT
AlucOR3-dF	CCTACATCGTCCTTCTTCA
AlucOR3-dR	AGATACTCAGCCGTTGAC
AlucOR4-dF	TGGTAGATTGCTTGAAGGA
AlucOR4-dR	GTATGGTGGAGTGCTGAT
AlucOR5-dF	ACGAACGAGACAAGAACT
AlucOR5-dR	CAGTATGGTGTAGAAGGAATAAG
AlucOR6-dF	ATGATATGTCTGAGCATTGG
AlucOR6-dR	AACCGATGTCTGACTCTT
AlucOR7-dF	ATCACGCAATAATCATCAAGT
AlucOR7-dR	CGACATTCTCACAATAGGATT
AlucOR8-dF	CATACAGTTGCGATTGGTA
AlucOR8-dR	TGTGAGTGTTGTGAGATTG
AlucOR9-dF	ATCATCAAGAACTCGTCCA
AlucOR9-dR	TTCGCAAGCCATAATCATC
AlucOR10-dF	TGGTCGGAGTGTATAACG
AlucOR10-dR	CAGTGAAGATAAGGATGTAAGAG
AlucOR11-dF	CCGCTTGGTATTCTTGTG
AlucOR11-dR	GTTGTTGTGGACTTGTCAA
AlucOR12-dF	TCGGCATCTACTCAATCTATT

AlucOR12-dR	ATGTCGGTCGTGAATGTT
AlucOR13-dF	CTTCTATTCTCGCTCTTGATG
AlucOR13-dR	ACTAACGGTTCGGTGATAA
AlucOR14-dF	TCCTGTCTCAACTTCATCTT
AlucOR14-dR	ATCTCCTTAATGCCGTGATA
AlucOR15-dF	AGCAAGAATACAATCCAACTC
AlucOR15-dR	GCCGCATAGACTCTACAA
AlucOR16-dF	CTTCCTCAACAGCATTACTC
AlucOR16-dR	ATTCACACGACCAACAATC
AlucOR17-dF	AAGGAATACGATGACTGCTA
AlucOR17-dR	CGATAGGTAGACTTGCCATA
AlucOR18-dF	CTACGATTACGGAGACAGTT
AlucOR18-dR	GCCAAGAGCAGAGTCAATA
AlucOR19-dF	TGGATTCTTCACTCTTGGAT
AlucOR19-dR	GTATAATTTGGGTAACGCAGA
AlucOR20-dF	AACCAAGCCTCTTCAGAA
AlucOR20-dR	GATAATCCTTCGTGCCTAAC
AlucOR21-dF	TTAGGATTGTTACCGTTAGTTG
AlucOR21-dR	GTGTGGCTCTCGTATCTAT
AlucOR22-dF	CGCAGCAATGTTACAAGA
AlucOR22-dR	TGAGTGATTCTAAGATACCGATA
AlucOR23-dF	TTGTTACTATGGCGAGGAA
AlucOR23-dR	AGGCGATTGACTTTGGTA
AlucOR24-dF	TCTATTCGTGGTACAGCAA
AlucOR24-dR	ATCGTCATCATTGTTCTTCTC
AlucOR25-dF	AAGGTGAAGACGATGAGTT
AlucOR25-dR	CCAAGTGTCAACAACCATAA
AlucOR26-dF	TTCACGACTATCAAGAACCT
AlucOR26-dR	CAACGACAACCAGAATCC
AlucOR27-dF	CAAGACAGCGAAGCAATT
AlucOR27-dR	TACTAATAGCGTGGCACAT
AlucOR28-dF	ATCTTGGAATGCCTTGTG
AlucOR28-dR	TTACTAACCTCATCAACTTCTG
AlucOR29-dF	CTGGCATGAAGTACAAGAAG
AlucOR29-dR	TCACGAACGAGTAGAATGT
AlucOR30-dF	GGAACCTCTGGAACTCAA
AlucOR30-dR	ACAAGTGCCAACATATTCAA
AlucOR31-dF	GAAGGAATCCGAATCAACTC
AlucOR31-dR	ATAACCATTGTGCTGCTTAG
AlucOR32-dF	GCTGAATCTGTGGTTGTG
AlucOR32-dR	AATTCTGCTTATTAGTTGATGGT
AlucOR33-dF	AAACCAAATCCCTCTCATAAC
AlucOR33-dR	GCAAGTTACCTTCATCTCTAC
AlucOR34-dF	TTATACGGTCGGACATTCA

AlucOR34-dR	CCAGAAGTTGACAGTAGGA
AlucOR35-dF	GGTCTTATACGGTATGATATTCG
AlucOR35-dR	CGCAAGCAATGATGGTAA
AlucOR36-dF	TACTTTCACCATCCCAATCT
AlucOR36-dR	GTAATTCGCAACCTTCCAA
AlucOR37-dF	TACCGTGTCATCGTAACC
AlucOR37-dR	CCACCAATCTACCTCGTAA
AlucOR38-dF	AACATTTAGTCCGCTTGC
AlucOR38-dR	AACGATAGAGCCTAGTTCAA
AlucOR39-dF	GACATATTCACATTGACTGGAT
AlucOR39-dR	CCGCTGTATAGTGGATTCT
AlucOR40-dF	AATCCATCAAGCATCACATC
AlucOR40-dR	ATGAACATAGGCGAACTAATG
AlucOR41-dF	TTATGGTGTTGGAGTGGAA
AlucOR41-dR	ACAGAAGTCATCATCGTATATTC
AlucOR42-dF	TTAGGACTCGCAGGAATC
AlucOR42-dR	GTGGCATTGAACAACAGT
AlucOR43-dF	TCCTGCTCAAGTCTGATG
AlucOR43-dR	TGTCCATTCACCTTCTTCA
AlucOR44-dF	GTCCTCACTACTACCTCATC
AlucOR44-dR	TCAGCATCAACTTCTTCCT
AlucOR45-dF	ACTTCTAACGACGATTGGA
AlucOR45-dR	GTGCTTGTCTCATCATCATAT
AlucOR46-dF	TTCTCAGGTGGATTGTTGTA
AlucOR46-dR	CGTGGTAGTGAATGATAAGTG
AlucOR47-dF	GGATACCGATAGGAATGGAT
AlucOR47-dR	AGATAATGAATAGCAAGACACAA
AlucOR48-dF	TCTGAATCGGTTGCTGTA
AlucOR48-dR	AATATGATGATGAAGTTCCAAGT
AlucOR49-dF	ATTCGTAACTGGCAGGAA
AlucOR49-dR	GGTTGTCGTAGTTGAGGA
AlucOR50-dF	CGTCGTATTCCTTCAACAC
AlucOR50-dR	TGAGCGTATCCAGTTCTAC
AlucOR51-dF	TTGCTTCTATTCGTTGTTCTT
AlucOR51-dR	TTCAATGCTCCTGTCCAT
AlucOR52-dF	GCCATTACACCTGATACAAG
AlucOR52-dR	CTCCACATTCCTCATCGTA
AlucOR53-dF	TCACTATCCACAACGAACT
AlucOR53-dR	ATTGATTGATTGACAGAGAAGAA
AlucOR54-dF	GAGCAGTAGACAGTGGATT
AlucOR54-dR	TGGAATAGAAGTGACCGAAT
AlucOR55-dF	CGTCAGCCATCATCTCAT
AlucOR55-dR	GTAGTTGTCAAGTAACACAGAAT
AlucOR56-dF	GACTACGATGAGACACCTT

AlucOR56-dR	AGTATCACGAGATGTATTACCA
AlucOR57-dF	TTCGGCTTACTGCTGTAT
AlucOR57-dR	TTCTTCAACATCATTCCTCAG
AlucOR58-dF	GCCGAAGAAGAAGATGATG
AlucOR58-dR	CAACATTGCGTGGTGTAA
AlucOR59-dF	AGTCCGTCATCATAGCAAT
AlucOR59-dR	CCAGAAGCATATTCAGGTAAG
AlucOR60-dF	AGAACGACCTCACTCAAC
AlucOR60-dR	GCAAGTCATTCAATAACCTCAA
AlucOR61-dF	GGTGACAGAGTTGGAGAA
AlucOR61-dR	TACAGAAGAGCGGTATGC
AlucOR62-dF	GGCGTTCAATTCCACTAC
AlucOR62-dR	TCTCTTCGGCAAGTATCTC
AlucOR63-dF	GGAAGTCTCAACGGAATAATG
AlucOR63-dR	CCTTGCTCTTGGTTAGTGTA
AlucOR64-dF	GATGAGCAGAACTATTGATGAA
AlucOR64-dR	ATCGCTTGAGAAGACACA
AlucOR65-dF	GAGCCAAGGAGTGAAGAA
AlucOR65-dR	GTAGAATACATCAAGACACAAGT
AlucOR66-dF	GTCCATCATAGGCGAGTTA
AlucOR66-dR	TCCATACACAAAGCGACTA
AlucOR67-dF	CAGGTTGTTCAGGTCCAT
AlucOR67-dR	GGTCTATCAAGTTCTGTCCAA
AlucOR68-dF	CGCATTCCTGTCATTATTCA
AlucOR68-dR	CCAGTAGTTGTCCGAGAG
AlucOR69-dF	TTGGAATCGGAGTGGAAT
AlucOR69-dR	TGCTTCGTTGGTTATTGAC
AlucOR70-dF	TACCAACAAGGCTCAGATAT
AlucOR70-dR	CCGTAGAGGATGTCAACA
AlucOR71-dF	CTCCACTCAGTTCACAATG
AlucOR71-dR	AGCAATTCGTATAGCATCAC
AlucOR72-dF	TTGGTGGTGGATGATTGA
AlucOR72-dR	CCGAGTCCTCAGTTACAG
AlucOR73-dF	CACTATCCGATATTCATCACAAT
AlucOR73-dR	GAGACGCAGAGACCTAAC
AlucOR74-dF	CGTTATTGCGACTGCTATT
AlucOR74-dR	CGATATGCTGGTTGATATGATTA
AlucOR75-dF	GGAGTTGATGAAGGATTGC
AlucOR75-dR	ACGAAGTGGACGATGAATA
AlucOR76-dF	CGCTCACACCATACTTAAC
AlucOR76-dR	GTAACTCCATTCCGTCAC
AlucOR77-dF	GTTGGTTGTGTCGTATCTG
AlucOR77-dR	GTTATGCTGTGCGTTCTC
AlucOR78-dF	ATAATGCTACGGCGACAA

AlucOR78-dR	TATGACCTGATGTTGGATTCT
AlucOR79-dF	AAGTGACATCATTGACAGAAT
AlucOR79-dR	TTCCGAACGCTCTCATAA
AlucOR80-dF	ACAACAAGAGCGAAGAGA
AlucOR80-dR	TGAGAATGAAGCGAGGTT
AlucOR81-dF	ATTCACATCACTCTCCTTATTG
AlucOR81-dR	TCCTCATCTGTCTCTTCATC
AlucOR82-dF	GCTGTTAGGACTAGAGGTT
AlucOR82-dR	AGAATCCGTTACTGAAGAGA
AlucOR83-dF	ACATCAGTCAGGCTTCAT
AlucOR83-dR	TCGTATTGAGTTCCAGTGTA
AlucOR84-dF	GCCAGGAAGAAGGACTAC
AlucOR84-dR	ACAGCGTTGATGACCATA
AlucOR85-dF	TCCGCTAATCTACAATACTACA
AlucOR85-dR	TTCGCTCTCATATCCATCAT
AlucOR86-dF	TCGTGAGAGTCTATGAGTTG
AlucOR86-dR	AGTTGTGGAATGTAGTCGTA
AlucOR87-dF	ACCTCCTCTCAACTCTCA
AlucOR87-dR	TCCATCCTTGTTATAGTCATCA
AlucOR88-dF	ACTGGCATCTACTGAATACA
AlucOR88-dR	CAGGTTGAAGAATGAATACGA
AlucOR89-dF	ATATGGAAGTGTTGGAGTCA
AlucOR89-dR	GGCAAGTATGTCGTGTAGA
AlucOR90-dF	ATATTCAACATTCTGCCGATT
AlucOR90-dR	GCCATTGTGACTTGTGATAA
AlucOR91-dF	TTCGTAATGTTATTGACTCAGAT
AlucOR91-dR	CTATTGCTATGGCGTACAC
AlucOR92-dF	TGATGAGGAAGTTATAGTGTTG
AlucOR92-dR	TTGATGTCCGAGTTGATTC
AlucOR93-dF	AAGACATTGGACGACAGAA
AlucOR93-dR	CAGCGAAGAGCATAACATC
AlucOR94-dF	ATCAAGCGTCGTCTAACC
AlucOR94-dR	TACACCTATGAACTGGAACAA
AlucOR95-dF	TTCTTGGCTGGAGTATCTAA
AlucOR95-dR	TCGGAATCTGTTCAGGAAT
AlucOR96-dF	AACGCTATCGGATGTCTT
AlucOR96-dR	GTGATGCTCTTGATTCTATTGA
AlucOR97-dF	ACCATTACAAGATACAGGAGTT
AlucOR97-dR	CGATGAGCAGCGAGAATA
AlucOR98-dF	GCAGATAGCAATGGAACTC
AlucOR98-dR	ATGACGACAACTACCAGAA
AlucOR99-dF	ACATTCTCCATCTCGTCTT
AlucOR99-dR	GCTCCGTTGAACATAAGG
AlucOR100-dF	CTTGGTAACTTGGTGACTATG

AlucOR100-dR	TCCTCTCGTGCCTTATTC
AlucOR101-dF	GTATGGCTACTGCTCCTT
AlucOR101-dR	GGTCGTCACAATGTTGATT
AlucOR102-dF	GCCGTGATGTGTTATATCG
AlucOR102-dR	TACCACTCCGTATTATACAAGG
AlucOR103-dF	TCTCGTCAAGGTTGGATT
AlucOR103-dR	GGTAGACTGCTTCGTGTA
AlucOR104-dF	AGAATATCAAGCATCATCACAA
AlucOR104-dR	GCACCAAGACAGAGACTA
AlucOR105-dF	CGGACGGAACTGTGATAT
AlucOR105-dR	TAAGCAGAGCAGCATAGAG
AlucOR106-dF	TCATTCACCACCAGAGAC
AlucOR106-dR	CGCCATAATCATTGAGATAGTAA
AlucOR107-dF	CCGTTGTTCATCGTCTTAG
AlucOR107-dR	TCTGTAGGTGATTCCATTCT
AlucOR108-dF	ACCTCATAGTATTGCTCCAA
AlucOR108-dR	TTGCCTGTAGAGTTGATTATTC
AlucOR109-dF	CGTGGTTGAGTCTCTTCT
AlucOR109-dR	CATCGTAGTTGCTGGATTC
